# Tumor biomarkers for diagnosis, prognosis and targeted therapy

**DOI:** 10.1038/s41392-024-01823-2

**Published:** 2024-05-20

**Authors:** Yue Zhou, Lei Tao, Jiahao Qiu, Jing Xu, Xinyu Yang, Yu Zhang, Xinyu Tian, Xinqi Guan, Xiaobo Cen, Yinglan Zhao

**Affiliations:** 1 Department of Biotherapy, Cancer Center and State Key Laboratory of Biotherapy, West China Hospital, Sichuan University, Chengdu, 610041 China; 2 West China School of Pharmacy, Sichuan University, Chengdu, 610041 China; 3 School of Medicine, Tibet University, Lhasa, 850000 China; 4 National Chengdu Center for Safety Evaluation of Drugs, State Key Laboratory of Biotherapy, West China Hospital, Sichuan University, Chengdu, 610041 China

**Keywords:** Cancer therapy, Tumour biomarkers

## Abstract

Tumor biomarkers, the substances which are produced by tumors or the body’s responses to tumors during tumorigenesis and progression, have been demonstrated to possess critical and encouraging value in screening and early diagnosis, prognosis prediction, recurrence detection, and therapeutic efficacy monitoring of cancers. Over the past decades, continuous progress has been made in exploring and discovering novel, sensitive, specific, and accurate tumor biomarkers, which has significantly promoted personalized medicine and improved the outcomes of cancer patients, especially advances in molecular biology technologies developed for the detection of tumor biomarkers. Herein, we summarize the discovery and development of tumor biomarkers, including the history of tumor biomarkers, the conventional and innovative technologies used for biomarker discovery and detection, the classification of tumor biomarkers based on tissue origins, and the application of tumor biomarkers in clinical cancer management. In particular, we highlight the recent advancements in biomarker-based anticancer-targeted therapies which are emerging as breakthroughs and promising cancer therapeutic strategies. We also discuss limitations and challenges that need to be addressed and provide insights and perspectives to turn challenges into opportunities in this field. Collectively, the discovery and application of multiple tumor biomarkers emphasized in this review may provide guidance on improved precision medicine, broaden horizons in future research directions, and expedite the clinical classification of cancer patients according to their molecular biomarkers rather than organs of origin.

## Introduction

### Brief history of tumor biomarkers development

Biomarkers are designated as “a biological molecule found in blood, other body fluids, or tissues that is a sign of a normal or abnormal process, or a condition or disease. A biomarker may be used to see how well the body responds to a treatment for a disease or condition” according to the National Cancer Institute (http://www.cancer.gov/dictionary). Tumor biomarkers exist in tumor tissues or body fluids such as blood, urine, stool, saliva, and are produced by the tumor or the body’s response to the tumor.^1^ The goal of the tumor biomarker field is to develop sensitive, specific, reliable, cost-effective, reproducible, and powerful detection and monitoring strategies for tumor risk indication, tumor monitoring, and tumor classification so that patients can receive the most appropriate treatment and doctors can monitor the progress, regression, and recurrence of the tumors.^2^ Since the discovery of Bence-Jones protein (BJP), the first tumor biomarker, in 1846, this field has been through many stages and has made significant and substantial progress with the joint efforts of researchers, clinical staff, and patients.

#### Discovery and exploration stage (1847–1962)

**Fig. 1:**
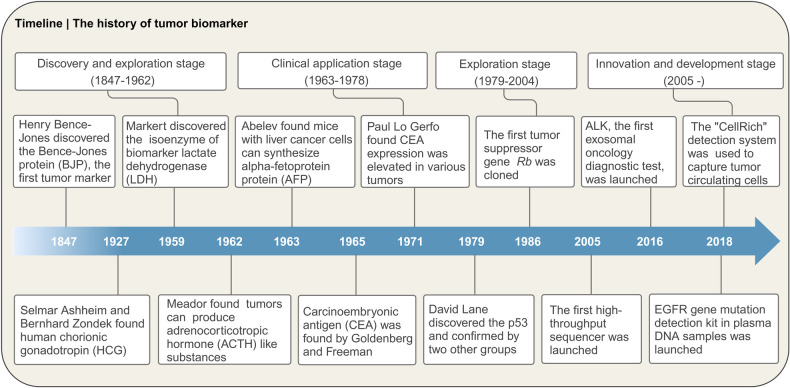
Timeline of the history of tumor biomarker

In 1847, Henry Bence-Jones described the findings of a large number of immunoglobulin light chains from the urine of a patient with multiple myeloma, and named it BJP, the first biochemical tumor biomarker described in diagnostic laboratory medicine^3^ (Fig. 1). The monitoring of BJP in urine has become one of the parameters related to the diagnosis and prognosis of multiple myeloma.^4^ The discovery of BJP marks the beginning of research on tumor biomarkers. Subsequently, hormones, isozymes, and other tumor biomarkers that displayed abnormalities during the occurrence and development of tumors were discovered. In 1927, Selmar Ashheim and Bernhard Zondek found a gonadal stimulating substance—human chorionic gonadotropin (HCG) from the blood and urine of pregnant women.^5^ Later on, HCG was identified as a tumor biomarker, which is frequently associated with gestational trophoblastic disease and testicular germ cell tumor.^6^ In 1959, lactate dehydrogenase (LDH), the first “isoenzyme”, was discovered in the bovine heart by Clement L Markert at Johns Hopkins University,^7^ and numerous clinical evidence subsequently demonstrated that LDH was an essential prognostic factor for different tumors.^8^ In 1962, Meador found that some tumors spontaneously produced adrenocorticotropic hormone-like substances, which hindered the normal secretion mechanism of adrenocorticotropic hormone and induced metabolic abnormalities dominated by hypokalemia.^9^ Despite the aforementioned breakthroughs in knowledge about tumor biomarkers, these biomarkers did not translate from bench to bedside for cancer diagnosis or monitoring.

#### Clinical application stage (1963–1978)

The next significant advances came from GI Abelev who is well known for his 1963 discovery that mice inoculated with liver cancer cells can synthesize alpha-fetoprotein (AFP)^10^ (Fig. 1). AFP has been used as a biomarker in clinical screening, diagnosis, prediction, and treatment evaluation of hepatocellular carcinoma (HCC).^11^ At around the same time (in 1965), Goldenberg and Freeman found that carcinoembryonic antigen (CEA) in fetal colon mucosa^12^ contributed a crucial part in tumor diagnosis and prognosis evaluation of lung cancer,^13^ breast cancer,^14^ ovarian cancer,^15^ colorectal cancer (CRC),^16^ etc. The discovery of AFP and CEA has promoted the clinical application of tumor biomarkers. However, the application of CEA as a tumor biomarker was later challenged by Paul Lo Gerfo and his colleagues in 1971. They measured the level of CEA in the serum or plasma of 674 hospitalized patients by radioimmunoassay (RIA). It was found that CEA expression level was elevated in the serum of patients with multitudinous diseases, but not cancer-specific, which hampered the potential absolute benefit of CEA assessment independent of other surveillance tools.

#### Exploration stage (1979–2004)

James Watson and Francis Crick’s discovery of the DNA double helix structure in 1953 ushered in a new era in tumor biomarker research.^17^ After this discovery, modern molecular biology significantly promoted the research on tumor biomarkers, with a large number of genes involved in tumor occurrence and progression being discovered. In 1979, p53 was found by David Lane and confirmed by other independent groups.^18,19^ Although being considered as a cell tumor antigen at the beginning, p53 was defined as a cancer suppressor gene in 1989^20^ and more than 50% of p53 was mutant in cancer patients.^21^ In 1981, Robert Weinberg and Geoffrey Cooper discovered the small fragments of DNA in transgenic experiments in which the transformation of mouse NIH/3T3 fibroblast cells transfected with DNA extracted from human tumors cell lines was successfully induced and soon followed the isolation of homologous oncogenes HRAS and KRAS from human tumors.^22,23^ This discovery paved the way for the development of the first KRAS^G12C^ inhibitor, sotorasib, which was approved by the United States Food and Drug Administration (FDA) in 2021 for non-small cell lung cancer (NSCLC) treatment.^24^ The first human oncogene, retinoblastoma (*Rb*) gene, was successfully cloned in 1986^25^ (Fig. 1). After that, large numbers of proto-oncogenes, oncogenes, tumor suppressors, receptors, and kinases were discovered, and some of them were successfully used as tumor diagnostic, prognostic, and therapeutic biomarkers.

#### Innovation and development stage (2005-)

The rapid development of science and technology is driving the field of tumor biomarkers to an innovation and development stage. An increasing number of methods and technologies have been developed and applied in tumor biomarker discovery and detection. The molecular biological technologies, such as genomics, transcriptomics, proteomics,^26^ metabolomics,^27,28^ the clustered regularly interspaced short palindromic repeats (CRISPR)/CRISPR-associated protein 9 (Cas9) gene-editing technology,^29,30^ and high-throughput sequencing,^31^ make it possible to obtain large-scale information on the diversity of tumor biomarkers. In 2005, the first high-throughput sequencer was launched, which contributed to the sequencing technology. In recent years, nucleic acid-based liquid biopsy for monitoring cancer has attracted much attention.^32^ For example, cell-free DNA (cfDNA) in the plasma of cancer patients contains tumor-derived DNA sequences, which can be used as biomarkers for the early detection of cancer, guiding treatment, and monitoring drug resistance.^33^ In 2016, ExoDx Lung (ALK), the world’s first exosomal oncology diagnostic test, was launched. It can be used for the diagnosis of NSCLC and the screening of NSCLC patients for targeted therapy with anaplastic lymphoma kinase (ALK) inhibitors. Subsequently, the epidermal growth factor receptor (EGFR) gene mutation detection kit in plasma DNA samples was launched in 2018 and used to screen patients for EGFR-targeted drug therapy. China’s self-developed “CellRich” circulating tumor cell detection system was approved by the National Medical Products Administration in 2018 and used to capture tumor circulating cells. Furthermore, the emergence of “precision medicine” has pushed the field of tumor biomarkers to a new stage, which requires the discovery of more effective tumor biomarkers and the integration of multiple tumor biomarkers to support personalized medicine.^34^

### Clinical application of tumor biomarkers

#### Early screening of tumors

Early screening of tumors is the most powerful public health tool that enables early detection, thus reducing annual cancer incidence, providing a higher chance of treatment, improving patient response to medical interventions, and prolonging patient survival, especially for those cancers with high mortality such as CRC.^35^ In addition to invasive or expensive screening methods such as endoscopy, low-dose computed tomography, and tissue biopsy, noninvasive and cost-effective screening based on biomarkers from body fluids including blood, stool, saliva, and urine has been gaining extensive attention. To date, thousands of these biomarkers including proteins, cytokines, metabolites, hormones, microRNA, and circulating DNA have been explored, and several of them have been successfully developed and used in the early screening of cancers.^2,34^ For example, AFP was the first blood biomarker used for the screening of HCC in the populations since 1964.^36^ After that, other biomarkers called “classical” tumor markers, such as CEA, carbohydrate antigen 19-9 (CA19-9),^37^ carbohydrate antigen 125 (CA125),^38^ prostate-specific antigen (PSA),^39^ and LDH,^40^ have been used in the clinical screening of various kinds of cancers. In addition to the “classical” tumor marker, a broad range of novel biomarkers have been explored in recent years, which include microRNA^41^ and other RNAs,^42^ microbial proteins,^43^ circulating nucleosomes,^44^ circulating tumor DNA,^45^ and circulating tumor cells.^46^ Albeit currently undergoing clinical trials or preclinical studies and unavailable in the market, they have great potential for clinical screening. As some biomarkers from body fluids may be difficult to detect because of fundamental biological barriers such as short circulation times and very low density, synthetic biomarkers including small-molecule, DNA-based, mammalian cell-based, and bacterial cell-based sensors have been developed to amplify tumor signals, thus enhancing the sensitivity and efficiency of early-stage tumor detection.^47^ New screening tests based on these novel techniques can be used in the clinic in the near future. Collectively, for the detection of early-stage cancer, the noninvasive or minimally invasive test is ideal, and developing such techniques is desirable in clinical applications.

#### Tumor auxiliary diagnosis

Due to the risk of false positive or negative results, relying solely on one biomarker level is not an accurate and reliable strategy for tumor diagnosis. Instead, the combination of tumor biomarkers with other methods such as tissue biopsy and endoscopy is a promising alternative to improve the effectiveness of screening.^48,49^ For example, the combined detection of AFP with cfDNA can improve the specificity of HCC diagnosis to 94.4%, which was superior to that of AFP alone in terms of higher sensitivity and better clinical correlation.^50^ The advantages of biomarker panels have been confirmed as compared with a single biomarker, especially a panel of biomarkers reflecting changes in independent pathways. The combination of periodin (POSTN) with CA15-3 and CEA for the diagnosis of breast cancer can improve the diagnostic performance of CA15-3 and CEA.^14^ For CRC, the detection of hemoglobin using fecal immunochemical testing in combination with transferrin in stool improves the diagnostic accuracy for CRC.^51^ The combination of various diagnosis strategies with biomarkers could result in an easier, faster, more accurate, and more specific diagnosis of cancer.

#### Prediction of tumor prognosis and curative effect

Precision stratification of cancer patients based on prognosis and therapeutic decision biomarkers has enabled the selection of treatment strategies and more effective treatments for individual cancer patients. One successful example is to distinguish the type of breast cancer by the expression of human epidermal growth factor receptor 2 (HER2), estrogen receptor (ER), and progesterone receptor (PR) in breast cancer tissues. These biomarkers help to identify the triple-negative breast cancer (TNBC) lacking the expression of ER, PR, and HER2 which is the most aggressive type of breast cancer associated with poor prognosis and limited treatment options, thus improving the management and treatment options with the ultimate goal of improving clinical outcomes.^52^ Moreover, identifying curative predictive biomarkers to distinguish patients who are most likely to respond to anticancer therapy from all cancer patients enhances therapeutic efficiency, decreases treatment costs, and avoids adverse events. For example, the implementation of patient selection prior to programmed cell death 1 (PD-1)/programmed cell death ligand 1 (PD-L1) inhibitors therapy by the combination of biomarkers reflecting tumor immune microenvironment and tumor cell-intrinsic features, such as PD-L1, tumor-infiltrating lymphocyte, tumor mutational burden, mismatch-repair deficiency, and gut microbiota, could enhance the treatment effect of anti-PD-1/anti-PD-L1 therapy in clinical practice.^53^

#### Tumor recurrence monitoring

The level of tumor biomarkers is valuable for indicating the disease recurrence of tumor patients. Some classic biomarkers for tumor diagnosis and prognosis, such as PSA, CEA, CA19-9, and CA72-4, are used for indicating the recurrence of cancers including prostate cancer, gastric cancer, breast cancer, and liver cancer.^54,55^ The CEA is increased in most liver recurrence cases of gastric cancer (90%), while the increase of CA19-9 after surgery in patients with gastric cancer could predict peritoneal recurrence more accurately (78.9%).^56^ In recent years, extensive molecular and genetic characterization of disseminated tumor cells and blood-based biomarkers have contributed significantly to monitoring cancer recurrence. Postoperative methylated septin 9 in plasma may represent a potential noninvasive biomarker for CRC recurrence monitoring in addition to CRC diagnosis and prognosis compared with CEA and CA19-9.^57^ The circulating tumor DNA (ctDNA) minimal residual disease (MRD) following treatment in solid tumors predicts relapse and highlights the application of this potentially transformative biomarker.^58^

Collectively, tumor biomarkers play an active role in all aspects of clinical application, such as early screening, diagnosis, prognosis, and relapse monitoring, and are of great value in helping patients prolong their survival and improve their quality of life. To date, excellent progress has been made in the discovery and application of biomarkers. Besides classical biomarkers used in clinical practice, recent advances in molecular biology technologies have significantly improved the discovery of new candidates for cancer management, but most of them are still in the early stage of development and validation. Great effort could be made to find new biomarkers with the right degree of specificity, sensitivity, and reliability, so as to provide evidence for individualized decision-making during the overall management of cancer patients. In this review, we summarize the current progress that has been made in cancer biomarker development and discuss the promise, limitations, and further challenges in biomarker development.

## Technologies used in the detection of tumor biomarkers

**Fig. 2:**
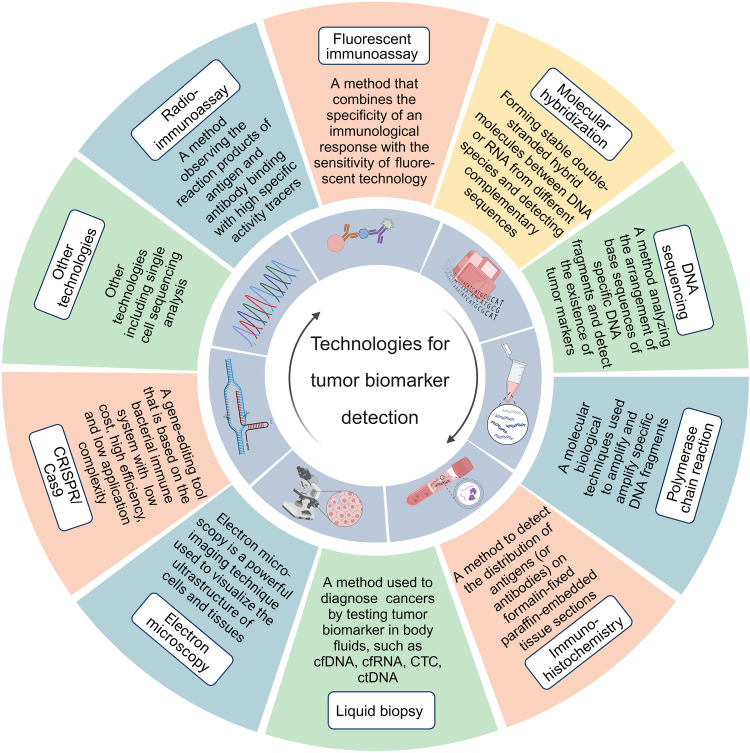
Technologies for the detection of tumor biomarkers

Multiple technologies have been developed for the detection of tumor biomarkers as follows (Fig. 2). In the past decades, various immunoassay methods have played crucial roles in the discovery of tumor biomarkers. Meanwhile, molecular hybridization technology and gene amplification detection technology further broaden the horizon of the application of tumor biomarkers in clinical practice. Immunohistochemistry (IHC) brings about the original distribution of biomarkers in fixed tissue. Furthermore, rapidly developed DNA sequencing and gene-editing technologies accelerate the speed and numbers of digging out prognostic and predictive tumor biomarkers. Other technologies, such as liquid biopsy and different microscopy technologies, as well as single-cell sequencing analysis,^59^ also provide tremendous convenience in cancer therapy.

### RIA technology

RIA technology is an analytical method proposed in the late 1950s by the United States chemist Solomon A. Berson and Rosalyn S. Yalow.^60^ It integrates immunologic and radiolabeling techniques to quantitate minute amounts of biological substances based upon the competition between labeled and unlabeled antigens for specific antibody sites, forming antigen–antibody complexes.^60,61^ RIA usually uses radionuclide ^125^I as a tracer, which has been widely used for its advantages of highly radioactive.^62–64^ In addition, RIA is advantageous in measuring a variety of immunoreactive substances for its high sensitivity, and specificity.^65,66^ For example, RIA is utilized in the detection of early-stage tumors, and is an effective method in combination with clinical pathological assay to provide comprehensive evaluations of tumors.^67^ However, the shortcomings of RIA are also prominent, such as isotope contamination due to the radioactive wastes, the requirement of specific safety equipment, and the excessive radiation exposure of workers induced by the long incubation time. which limits its wide use.^65,68^ RIA tends to be eliminated with the rapid update of other immunoassay methods, such as enzyme-linked immunosorbent assay and fluorescent immunoassay (FIA), which use other substances such as fluorescent dye instead of radioactive isotopes to label antigens.

### FIA technology

FIA, combining the specificity of the immunological response with the sensitivity of fluorescent technology, is a popular and fast-growing nonisotopic immunoassay technology. As a new immunoassay technology using fluorescein-labeled antibodies or antigens as tracers, the principle of FIA is similar to enzyme-linked immunosorbent assay. The fluorescein is chemically bound to the antibody (or antigen) molecule, and after that, the latter is combined with the matching antigen (or antibody). The fluorescence is observed, or the fluorescence intensity is measured by a fluorescence detector which determines the presence, distribution, and content of antigens (or antibodies) in samples. FIA has the advantages of high specificity, high sensitivity, and good practicability with cheap, stable, and safe reagents.^69^ Moreover, FIA avoids the risk of handling radioactive materials. Thus, FIA is widely used in the biomedical field in the measurement of drugs, hormones, and proteins; the identification of antibodies, and the quantification of antigens.^70^

The development of various fluorescent probes and instruments also contributes to the continuing evolution of FIA.^2,71,72^ Multiple FIA-related technologies with high detection sensitivities and various measurable properties have been developed, including fluorescent excitation transfer immunoassay, fluorescence polarization immunoassay, and time-resolved fluorescence immunoassays.^73–77^ For example, the multicolor quantum dots based on fluorescence polarization immunoassay have been applied in the detection of tumor biomarkers such as α-AFP and CEA.^78^

### Molecular hybridization technology

Molecular hybridization technology is an important method for the investigation of gene expression and genome function by assessing chromosomal aberration using a fluorescent probe.^79^ The principle of molecular hybridization technology is to form stable double-stranded hybrid molecules between DNA or RNA from different species, thereby detecting complementary sequences or recognizing binding sites of transcription factors. Common molecular hybridization techniques include fluorescence in situ hybridization (FISH) and in situ hybridization.^79,80^ In situ hybridization uses labeled complementary DNA or RNA strands to localize specific DNA or RNA sequences on chromosomes or tissue sections fixed on slides (in situ), and the FISH technique helps to localize genes to different chromosomal locations. They are all molecular tools in cancer diagnosis, treatment, and prognosis.^79,81^ With the advantages of easy manipulation, fast hybridization process, possible automation of process and scoring, the FISH technique is wildly utilized to detect the tumor biomarkers in diagnosis and metastasis prognosis, such as the analysis of circulating tumor cells (CTCs) obtained from the patient blood sample^82^ in various of cancers, including lung cancer, glioma, breast cancer, ovarian cancer, and soft tissue sarcomas. Extensive prognostic and predictive biomarkers, such as ALK, mesenchymal-epithelial transition factor (c-MET), and ROS1, are identified by FISH.^82^ Significantly, the timeless and costless FISH remains a gold standard in ALK-rearrangements NSCLC.^83^ In 2011, a novel anticancer drug crizotinib and its companion, the ALK FISH probe detection kit, were simultaneously approved by the FDA, which highlights the crucial position of the FISH assay in guiding ALK-targeted therapy.^84^

In short, FISH is an increasingly demanded tool for biomarker research and personalized medicine despite the fact that the process of FISH may be time-consuming and costly when performed with standard chemicals and the retention of the fluorescence is limited.^82^

### Gene amplification detection technology

Polymerase chain reaction (PCR), a molecular biological technology used to amplify specific DNA fragments, is an invaluable tool for the assessment of nucleic acids in tissues and body fluids. It can synthesize and amplify specific DNA into billions of copies in a few hours by separating the DNA into two strands and incubating it with oligonucleotide primers and DNA polymerase in vitro.^85^ PCR technology has developed to the third generation since the invention of Kary Mullis in 1985,^86^ and holds a pivotal position in biological research. The PCR technology includes three major steps: denaturation of double-stranded (ds) DNA template, annealing of primers (forward and reverse primers), and extension/elongation of dsDNA molecules.^85^ The quantitative real-time PCR (qPCR)-based assay is considered to be the gold standard for prognostic and predictive biomarker analysis for the quantitative advantage.^85^

The application of PCR in diagnostic gene mutation analysis, such as the B-raf proto-oncogene (BRAF), EGFR, Kirsten rat sarcoma viral oncogene homolog (KRAS), neuroblastoma RAS viral oncogene homolog (NRAS), and phosphatidylinositol-4,5-bisphosphate 3-kinase catalytic subunit alpha (PIK3CA) genes from the blood, is meaningful in initial cancer stratification and the monitoring of cancer progression. Moreover, several PCR assays approved by the FDA are used for the diagnosis of KRAS mutation status in formalin-fixed paraffin-embedded tissue, thereby guiding anti-EGFR antibody treatment for metastatic CRC.^87^ Similarly, qPCR assays are effective in the detection of MRD in leukemia, such as the quantification of BCR-ABL-positive cells post-induction chemotherapy/transplantation in acute lymphoblastic leukemia (ALL).^85^ PCR technology is also widely used to detect abnormal genes and abnormal mRNA amplification in tumors, such as MYCN amplification in neuroblastoma.^88^ Ligand-targeted PCR is essential for the detection of folate receptor-positive circulating tumor cells as a potential diagnostic biomarker in pancreatic cancer.^89^

PCR methods are of great advantages in the detection of nucleic acid biomarkers, including relatively simple manipulation, providing rapid inexpensive diagnosis with good sensitivity, valuable for clinical molecular pathology.^87^ Nevertheless, several intrinsic drawbacks of PCR that restrain its application have room for improvement, such as the requirement for instruments, experienced operators, laboratory setting, and sophisticated operations.^90^ Collectively, PCR is a valuable tool in tumor biomarker detection, while novel PCR-based methods remain to be explored to meet the needs of patient monitoring in clinic.^87^

### DNA sequencing technology

DNA sequencing technology is a commonly used technology in molecular biology research, which is used to analyze the arrangement of the base sequence of specific DNA fragments. The world’s first method of DNA sequencing was invented by British biochemist Frederick Sanger, who performed the first complete DNA genome sequencing, bacteriophage ϕX174 in 1977.^91,92^ Since that, DNA sequencing technology has witnessed rapid development, which is now in its fourth generation of DNA sequencing technology.^93^ It not only opens up new perspectives in traditional biology, medical research, and other fields, but also promotes the further development of bioinformatics, molecular genetics,^94^ genomics,^95^ precision medicine,^96^ and other disciplines, which advances the progress of life science research.

DNA sequencing technology is not only the gold standard for microbial identification but can also be used to detect the existence of tumor biomarkers which indicate the occurrence and development of tumors. At present, next-generation sequencing technology (NGS) is the most widely used among four DNA sequencing technologies in clinical practice, which can detect multiple genomic alterations including nucleotide substitutions, small insertions, deletions, copy number variations, and chromosomal rearrangements. NGS promotes the identification of somatic mutations associated with acute myeloid leukemia (AML), melanoma, mesothelioma, small cell lung cancer (SCLC), breast cancer, and prostate cancer.^97^ For example, NGS was applied to detect mutations of many cancer-related genes, such as TP53,^98^ phosphatase and tensin homolog (PTEN),^99^ KRAS,^100^ and breast cancer type 1/2 susceptibility protein (BRCA1/2).^101^ These detections are valuable for assessing the family history and the risk of tumorigenesis, and improving clinical diagnostics.^97^ Except for providing a high sensitivity in gene mutations, NGS is dramatically cost-effective and less time cost compared with current PCR-based tests. For example, the cost of using PCR to detect RAS mutations is as high as several thousand dollars, while NGS only costs one-third for detecting the same mutations. Notably, NGS simultaneously sequences the remaining gene samples in the same pathway or multiple samples in a single sequencing run with high speed and accuracy, which avoids incurring additional operating costs.^97^

Moreover, RNA sequencing has been utilized in multifarious aspects of cancer management, including prognostic and predictive biomarker identification, the characterization of cancer heterogeneity, and the monitoring of drug resistance. Some special genomic biomarkers, including miRNA, lncRNA, and circRNA, have been discovered by RNA sequencing.^102^ For example, isocitrate dehydrogenase (IDH) mutation which is a good prognostic biomarker for glioma, and nuclear cyclooxygenase 2 combined with HER2 which serve as potential biomarkers for the diagnosis and prognosis of CRC, are identified by RNA sequencing.^102^

In conclusion, the advent of sequencing technology sequences individual cancer genomes, which opens a new chapter in precision cancer therapy. Novel sequencing technologies have the potential to decode massive amounts of cancer genomes rapidly and cheaply to benefit cancer precision therapy.

### IHC technology

IHC, a technology used to detect the distribution of antigens (or antibodies) on formalin-fixed paraffin-embedded tissue sections,^103^ identifies targets through antigen–antibody interactions, and the antibody binding site is identified by direct labeling or secondary labeling method.

IHC is a gold standard and ubiquitously applied technology in cancer identification and diagnosis, especially in assessing biomarkers used for characterizing tumor subtypes, confirming tissue origin, distinguishing metastasis from primary tumor, providing prognosis information, stratifying patients for treatment selection, and predicting therapy response in various cancers.^104–109^ The American Society of Clinical Oncologists and College of American Pathologists (ASCO/CAP) provides the HER2 scoring guidelines to determine breast cancer pathological classification and clinical stage by using IHC-based staining intensity and the percentage of HER2+ cells in cancer tissues.^110^ IHC is used to detect p53 in cancer tissues.^111,112^ The detection of the excision repair cross-complementation group 1 protein by IHC has been approved for predicting the response of NSCLC patients to chemotherapy.^105^

As an indispensable technology, IHC holds the unique advantage of correlating the presence of protein with its location in tissues or cells compared with other protein detection methods, which is essential for illustrating protein function in normal and pathological tissues.^113^ Moreover, IHC can be operated by easy preparation and automated manipulation.^82^ However, several limitations still exist in IHC, especially a lack of reproducibility. Conflicting results often occur when different antibodies are used. The variables of the protocol affect the reliability of IHC, including the fixation time of tissues, the absolute level of the antigen, the affinity and concentration of antibody, and the sensitivity of the detection system.^111^ Thus, high-quality control of regents, standardized protocols, automated IHC, or combined IHC with transcriptomics will improve the accuracy, reproducibility, and reliability of IHC and accelerate its application in the discovery and validation of biomarkers.^114^

### Liquid biopsy technology

Liquid biopsy, a minimally invasive methodology, is used to obtain tumor-derived information from body fluids so as to facilitate cancer diagnosis.^115^ Currently, liquid biopsy is used to detect cfDNA, cell-free RNA, CTCs, extracellular vesicles,^116,117^ ctDNA,^118^ circulating RNA, and exosomes^119,120^ in blood or other body fluids.^116,121^ Liquid biopsy can enhance patient overall survival (OS) by improving early cancer detection and monitoring treatment response continuously. Thus, liquid biopsies are widely used in the clinical biomarker screening of tumors, such as endometrial cancer,^122^ lung cancer,^123^ pancreatic cancer,^124^ CRC,^125^ melanoma,^126^ renal cell carcinoma (RCC),^127^ breast cancer, ovarian cancer, cervical cancer, and bladder cancer.^128^ In addition, liquid biopsy technology is also utilized to detect and monitor KRAS, BRAF, and EGFR mutations in patients with lung cancer, CRC, and breast cancer.^128^

Liquid biopsy can reduce the risk of biopsy by noninvasive sampling,^128^ and it has the advantages of convenient sampling and easy operation. Moreover, liquid biopsies have the potential to better detect heterogeneity across regions of the tumor.^115^ Although there are still some challenges to overcome in terms of assay sensitivity and specificity, liquid biopsy technology provides new opportunities for personalized cancer treatment and has the potential to revolutionize the field of oncology.

### Electron microscopy technology

Electron microscopy (EM), a powerful imaging technique used to visualize the ultrastructure of cells and tissues with high resolution, is applied based on a special type of microscope, electron microscope.^129,130^ The first electron microscope was built in 1931 by a German engineer and academic professor Ernst Ruska.^131^ The electron microscope uses signals obtained from the interaction between an electron beam and the sample to achieve information about sample structure, morphology, and composition.^132^

EM has been widely used in investigating tumorigenesis-related cellular and subcellular change^133^ and observing the ultrastructure changes in cancer cells,^134^ as well as in clinical applications for cancer diagnosis and treatment.^134–136^ The ultrastructural features of tumor cells by EM can provide vital clues such as evidence or biomarkers of cytodifferentiation for correct diagnosis, which is difficult for diagnosis of light microscopy.^137,138^ Especially, the ultrastructural examination provided by EM is necessary for the precise categorization of biomarkers in apparently undifferentiated carcinoma.^138^ Thus, EM is useful in the differential diagnosis of tumors, particularly in small-cell “undifferentiated” tumors, such as neuroblastoma, rhabdomyosarcoma, Ewing’s sarcoma, undifferentiated squamous cell carcinoma (SCC) of the lymphoepithelioma type, and malignant lymphoma, amelanotic melanoma, and spindle-cell carcinoma.^137^ Scanning electron microscopy has been used as an alternative to examine the morphology of exosomes which is a diagnostic biomarker usually detected by liquid biopsy.^139^ In conclusion, EM is a valuable complementary tool for tumor diagnosis, especially providing valuable information on tumor differentiation which is difficult to define by light microscopies.^134,140^

### CRISPR/Cas9 technology

The CRISPR/Cas9 technology is a gene-editing tool that is based on the bacterial immune system. The basic principle of CRISPR/Cas9 is to use a guide RNA molecule to direct a nuclease, Cas9, to a specific target gene. The nuclease then cleaves the DNA at the target site, allowing for precise modifications of genome.^141,142^

By using CRISPR/Cas9 technology to precisely edit cancer-related genes, researchers have created highly specific molecular probes for the detection of cancer biomarkers in body fluids, such as blood, urine, and saliva. CRISPR/Cas9 system is extensively used for different kinds of cancer biomarkers including virus nucleic acids, ctDNAs (i.e., EGFR mutation), miRNAs (i.e., miR-17, miR-31), proteins (i.e., TGF-β1, CEA, PSA, AFP), and extracellular vesicles.^90^ CRISPR/Cas9 can combine with other assays for tumor biomarker identification, such as qPCR, FISH, and nanotechnology, providing an efficient way for tumor biomarker discovery. Moreover, CRISPR/Cas9 has exerted significant effects in the treatment of cancers, such as pancreatic cancer, prostate cancer, breast cancer, ovarian cancer, liver cancer, and CRC.^143^

The CRISPR/Cas9 system enjoys some advantages, including low cost, high efficiency, low application complexity, easy-to-operate, and time-saving.^30^ The exquisite specificity is also a character of the CRISPR/Cas9 system which could distinguish single base mismatch in target nucleic acid.^90^ Moreover, CRISPR/Cas9-based nucleic acid amplification strategies exhibit high detection sensitivity comparable with PCR. However, several aspects of CRISPR/Cas9-based diagnosis still need to be improved. CRISPR/Cas9-based analysis requires the fluorescence spectrophotometer and electrochemical workstation which is inconvenient for detection. Thus, the portable and quantitative detection strategy should be further explored to monitor cancer biomarkers. Cancer progression is influenced by the level of multiple biomarkers such as various miRNAs, ctDNAs, and proteins, which makes the design of the high-throughput CRISPR/Cas9-based strategy for cancer biomarkers detection promising and significant.^90^ In conclusion, CRISPR/Cas9 technology is a powerful gene-editing tool that holds great promise and opportunities for the development of personalized cancer management.^144^

## Classification of tumor biomarkers

**Fig. 3:**
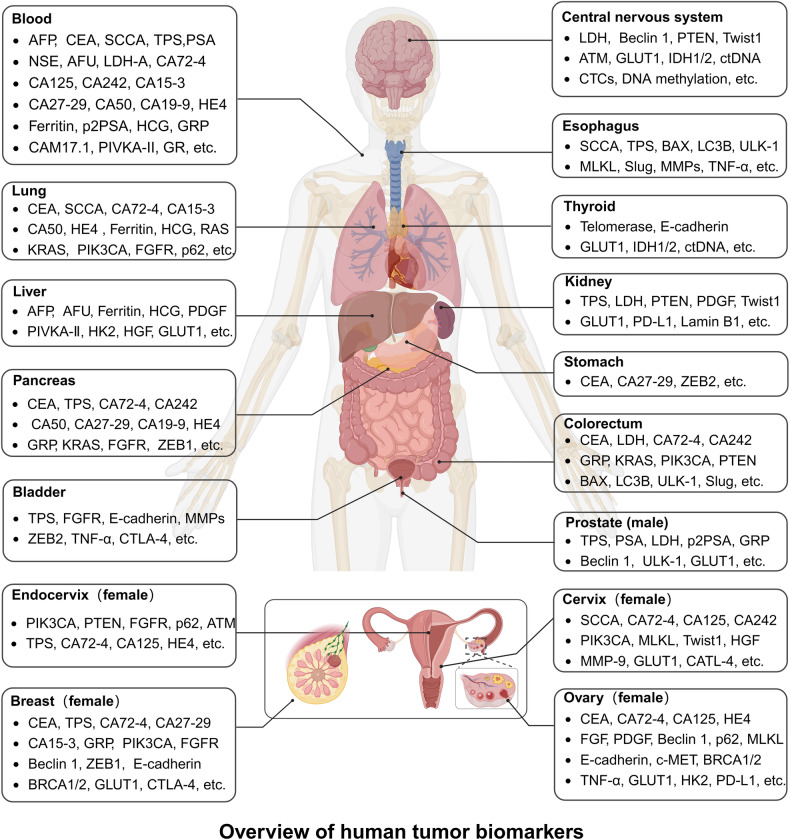
Overview of human tumor biomarkers

Tumor biomarkers are diverse and can be classified by different standards. Here, we divide tumor biomarkers by tissue origin: tumor biomarkers derived from blood, tumor tissues, and other biofluids such as feces, urine, and saliva (Fig. 3).

### Tumor biomarkers derived from blood

Tumor biomarkers in the blood are highly significant for tumor diagnosis and treatment. They have vital reference values for early tumor diagnosis, tumor stage assessment, anticancer strategy selection, treatment response monitoring, and prognosis.^2,145^ Here, we summarize the common tumor markers in blood and their roles in cancers.

#### Embryonic antigen tumor biomarkers

The 1960s saw the discovery of AFP and CEA, two tumor biomarkers that are still widely employed as tumor biomarkers. AFP and CEA are embryonic antigen substances which are proteins that only appear in the fetal period and gradually decline and disappear in adulthood.^146–149^ The reemergence of these embryonic antigens in cancer patients may be related to the activation of certain genes that have been turned off in adulthood when malignant cells transform, and these genes make embryonic antigens. While there aren’t many embryonic antigen tumor biomarkers, the ones that exist are crucial biomarkers for cancer care in clinical practice.

#### AFP

AFP, first discovered in 1956 by Bergstrand Czar,^146^ is a 3–5% carbohydrate-containing single-chain glycoprotein.^150^ Encoded by the AFP gene located in the q arm of chromosome 4 (4q25), AFP is a member of the albuminoid gene superfamily.^151^ As the amino acid sequences of AFP and albumin are very similar and highly homologous, AFP is considered as an analog of serum albumin in the fetal period and is the main protein in fetal circulation. At 18 months after birth, albumin synthesis gradually increases, and AFP concentration gradually decreases. The concentration of AFP in healthy adult serum is less than 10 μg/L.^147^ AFP is currently the most widely used tumor biomarker for HCC and has been used for more than 60 years. Elevated AFP can be seen in ~80% of HCC patients.^152,153^ Thus, AFP is currently applied for HCC screening, especially in China, Japan, Africa, and Alaska. The international academic community recommends limiting the reference value of AFP to 20 μg/L. Moreover, early-stage HCC is frequently detected by AFP detection combined with ultrasound.^154^ Tumor prognosis and treatment monitoring are additional applications for AFP. In patients with HCC, a sharp increase in AFP indicates tumor recurrence or metastasis. AFP >200 μg/L after surgery indicates incomplete removal or metastasis of HCC.^155^ Nonetheless, AFP levels are not the perfect diagnostic criteria for HCC. Approximately 40% of patients with early-stage HCC express normal or acceptable AFP levels. The elevation of AFP levels is observed in patients with chronic liver diseases, including ~20% of patients with hepatitis and 40% of patients with cirrhosis.^156^

#### CEA

CEA was first extracted from human CRC tissues and embryonic tissues in 1965, hence it was named for CEA.^148^ CEA belongs to a family of glycoproteins on the cell surface, and its gene is located on chromosome 19q.^157^ The production of CEA in the digestive tract starts at the early fetal stage (week 9–13). In addition to normal adult tissues such as the colon, stomach, cervix, sweat glands, and prostate, CEA is highly expressed in various tumors.^149^

As a broad-spectrum tumor biomarker, CEA is elevated in 70% of CRC, 55% of pancreatic cancer, 50% of gastric cancer, 45% of lung cancer, 40% of breast cancer, 40% of urethral carcinoma, and 25% of ovarian cancer patients.^149,158,159^ Serum CEA levels are proportional to tumor burden. Accordingly, CEA is applied to aid the diagnosis, determine the prognosis, monitor recurrence, and evaluate the therapeutic efficacy in cancer patients.^160^ In patients with breast cancer, CEA is one of the most frequently used biomarkers in the diagnosis, prognosis, and prediction of survival for different breast cancer molecular subtypes.^161^ In CRC patients, CEA level is a meaningful prognostic and diagnostic biomarker. The levels of CEA predict the 5-year survival rate of patients: 69% of patients have a CEA level below 5 ng/mL, 44% have a level of 5–200 ng/mL, and only 7% have a level equal or greater than 200 ng/mL.^162^ The elevated CEA level also has a bearing on poor prognosis and progression of lung adenocarcinoma patients with mutant EGFRs, and gastric cancer patients with lymph node metastasis.^163^ Additionally, CEA is also used for efficacy evaluation and recurrence detection after tumor treatment.^164^

Nevertheless, CEA lacks good sensitivity and specificity, which renders CEA inappropriate for tumor screening. A combination of CEA with other biomarkers could improve its actual significance in clinical practice.^149,164^

#### SCCA

Squamous cell carcinoma antigen (SCCA), a tumor-specific antigen, was first isolated from cervical SCC tissue by Kato and Torigoe in the 1970s.^165^ Initially, SCCA was used as a tumor biomarker for cervical cancer, and it has a high independent diagnostic value in cervical cancer.^166^ The serum level of SCCA correlates with the stage, the degree of invasion, recurrence, and the progression of cervical SCC.^159^ Cervical cancer patients with a high-level of pretreatment serum SCCA exhibit a higher risk for death than patients with low serum SCCA. Pretreatment SCCA cutoff ranging from 1.1 to 40.0 ng/mL is related to recurrence and death.^166,167^ Subsequent research has revealed that SCCA exists in tumors in the mouth, pharynx, esophagus, lung, and other tissues. In particular, high levels of SCCA have been found in multiple SCCs including lung cancer, esophageal cancer, and genitourinary system cancer in addition to cervical cancer, suggesting its essential role in the diagnosis and prognosis of the above cancers.^168,169^ Furthermore, elevated serum SCCA is associated with the therapeutic effect of postoperative chemotherapy in esophageal squamous cell carcinoma (ESCC),^170^ and with tumor-node-metastasis stage in head and neck squamous cell carcinoma (HNSCC).^171^ Peripheral SCCA has also been extensively utilized as one of the tumor biomarkers for monitoring NSCLC and predicting patients’ response to platinum combination chemotherapy, and serum SCCA level accurately reflects the survival status of patients.^169^ Despite its limited sensitivity in routine tests, SCCA is still a valuable diagnostic and prognostic biomarker in cancers.

#### TPS

Tissue polypeptide-specific antigen (TPS) is an M3 antigen determinant on the 18 fragments of cell keratin.^172^ TPS is synthesized in the S and G2 phases of the cell cycle, and the level of TPS in serum specifically indicates the proliferative activity of cells.^173^ The levels of TPS mostly depend on the number of cells in the proliferative phase instead of the total number of tumor cells, which is different from other tumor biomarkers.^173^ The serum levels of TPS are noticeably increased in multiple tumors, such as endometrial cancer, bladder cancer, NSCLC, skin cancer, carcinoma of male urethra, prostate cancer, pancreatic cancer, CRC, gastric cancer, esophageal cancer, neuroblastoma, and nephroblastoma.^174–177^ Thus, TPS has been employed as a serum tumor biomarker. Due to its lack of sensitivity and organ specificity, the prime application of TPS is monitoring treatment efficiency, and predicting tumor progression and recurrence, rather than diagnostic utility. In breast cancer patients, elevated serum levels of TPS could predict distant metastasis after treatment,^178^ and are recognized as an independent prognostic factor for disease-free survival (DFS) and OS of patients.^179^ In gastric cancer, TPS is applied in monitoring the palliative treatment response of patients with a 75% detection rate.^180^ The potential clinical role of TPS in RCC prognosis has also been demonstrated.^181^

Additionally, it is worth noting that TPS levels can alter in response to some pathological and physiological conditions, such as chronic pancreatitis, liver cirrhosis, ovulation, and menopausal status. Thereby, TPS in combination with other prognostic factors is necessary to improve the clinical use of serum TPS levels in predicting patient prognosis and facilitating the individualization of therapy for cancer patients.^182^ Further clinical studies are required to fully determine the utility of TPS alone or in combination.^182,183^ In conclusion, TPS has a unique value in the prediction of recurrence and metastasis, treatment monitoring, and prognostic evaluation in cancer patients.^177^

#### PSA

PSA, a serine protein kinase-releasing enzyme specifically secreted by the epithelial cells of prostate,^184^ is encoded by the prostate-specific gene kallikrein 3 which is a member of the tissue kallikrein family.^185^ PSA was first identified in the late 1970s.^186^ The elevated serum PSA levels represent prostate pathologies including prostatitis, benign prostatic hyperplasia, and prostate cancer.^187,188^ For the early diagnosis of prostate cancer, the positive cut-off value of serum PSA is greater than 10 ng/mL. In 1986, PSA was approved by the FDA as an adjunctive test for the detection of prostate cancer in men over the age of 50.^185,189^ Subsequently, in 1994, PSA was approved by the FDA as a diagnostic biomarker.^189^ Later on, PSA became popular in prostate cancer detection and patient management including screening, risk stratification for recurrence, surveillance following diagnosis, and monitoring therapy.^186,188^ Total PSA essentially consists of free PSA and bound PSA, and the higher percentage of the free PSA is connected to the lower the cancer risk. Studies have shown that a free PSA percentage >25% indicates the cancer risk is <10%, but a free PSA percentage <10% means the cancer risk is ~50%.^187^

However, PSA holds a poor specificity of 20–40% in prostate cancer diagnosis. Some noncancerous pathologies such as inflammation, trauma, or benign prostatic hyperplasia may also elevate the PSA level, which leads to a high rate of false positives. Besides, PSA is unable to differentiate between indolent and aggressive forms of prostate cancer, which may ignore aggressive prostate cancer with low initial serum PSA levels.^187,190^ All the aforementioned factors make prostate cancer now an “overdiagnosed” and “overtreated” cancer.^185^ To sum up, PSA level is a promising biomarker in prostate cancer diagnosis and prediction.

#### NSE

Neuron-specific enolase (NSE), a member of the enolase gene superfamily in glycolysis, was originally identified by Moore and McGregor in 1965 as an enzyme enriched in neurons and peripheral neuroendocrine cells.^191,192^ NSE consists of five dimeric isoenzymes with three different subunits, α, β, and γ, and is a sign of mature neural differentiation.^193^ Cell proliferation accelerates in response to oncogenic transformation in either central or peripheral neurons, accompanied by enhanced glycolysis and elevated NSE expression. Consequently, NSE plays pivotal roles in diagnosis, prognosis, and treatment efficacy evaluation in cancers originating from neural and neuroendocrine.^194,195^ Moreover, elevated NSE is also observed in SCLC which is with neuroendocrine properties. Serum NSE is currently believed to be a clinically potential biomarker for staging, monitoring treatment, and predicting relapse of SCLC.^196,197^ Interestingly, NSE also exerts a significant function in NSCLC. An analysis of 363 patients with advanced and metastatic NSCLC showed that patients with high NSE level (≥26.1 ng/mL) have significantly shorter progression-free survival (PFS) (5.69 vs 8.09 months) and OS than patients with low NSE level (11.41 vs 24.31 months).^191^ Besides, increased serum NSE levels are found in 30–69% of patients with NSCLC,^198,199^ which is in accordance with a study of 621 NSCLC patients which shows high NSE level (>12.5 ng/mL) is a prognosticate of poor outcome.^200^ Thus, serum NSE level is a predictive biomarker of cancer treatment response and an independent prognostic factor.^191^

#### AFU

α-l-Fucosidase (AFU), consisting of two isoforms, AFU1 and AFU2, which are encoded by *FUCA1* and *FUCA2* genes, respectively, is a lysosomal enzyme that clears the terminal α-l-fucose residues from glycoproteins.^201^ AFU is involved in the metabolism of glycoproteins, glycolipids, and oligosaccharides, and is widely distributed in human tissues and blood. The serum AFU level remains low under normal circumstances. While the serum AFU level increases rapidly as long as tumors attack the body, its level is closely related to the tumor stage and size.^202^ Multiple studies have shown that AFU is one of the most valuable biomarkers for HCC detection, with 85% sensitivity and 91% specificity.^203,204^ 85% of patients with HCC can be diagnosed with AFU detection six months prior to the ultrasonography detection.^205^ Patients with a preoperative AFU >35 U/L have a lower recurrence-free survival (RFS) rate and OS rate than those with AFU ≤35 U/L, and they tend to form macrovascular invasion. Therefore, serum AFU is of great significance for judging the treatment effect, prognosis, and recurrence of HCC.^205,206^ Besides, the low AFU levels are significantly associated with longer OS in ESCC, which indicates that AFU is a potential prognostic biomarker for long-term survival in patients with early-stage ESCC.^207^ However, the serum levels of AFU are also mildly elevated in certain nonneoplastic conditions such as cirrhosis, chronic hepatitis, and gastrointestinal bleeding.^203,208^ Presently, the combination of AFU and AFP biomarkers is used in the diagnosis of HCC, which enhances the diagnostic specificity, and makes the diagnosis more stable and reliable for high-risk groups such as hepatitis and cirrhosis.^155^

#### LDH

LDH, an enzyme that catalyzes the reversible transfer of pyruvate to lactate and NADH to NAD + , consists of two different isoforms, lactate dehydrogenase A (LDHA) and LDHB.^209,210^ The two isoforms can form five homotetramers or heterotetramers with different functions.^210^ In the reverse reaction, LDHB is more effective at converting lactic acid back to pyruvate than LDHA is at converting pyruvate to lactic acid.^211,212^ Multiple factors, such as the oncogene c-Myc and hypoxia-inducible factor (HIF-1α), stimulate the transcription of LDHA,^213,214^ which results in the overexpression of LDHA in most tumor tissues.^215^

High expression of LDHA provides cancer cells with many benefits, and multiple studies have proved that high levels of serum LDH are associated with the proliferation of cancer-initiating cells, enhanced aggressiveness and metastasis, the poor prognosis of cancers, as well as radiation and chemotherapy resistance.^216–218^ The serum LDH level is considered to be a primary predictor of prognosis in patients with adverse prognosis and distant metastases in melanoma, RCC, and CRC.^216^ Accordingly, an analysis of 76 studies comprising 22,882 patients with solid tumors reveals that high serum LDH levels are linked to poor survival in patients with solid tumors, in particular in melanoma, prostate cancer, and RCC, and is a valuable and affordable prognostic biomarker in metastatic cancers.^40^ Serum LDH levels are closely correlated with OS in an analysis of 2507 cancer patients with brain metastasis^216^ and are a poor prognosticator for OS and DFS in nasopharyngeal carcinoma (NPC) patients. Furthermore, the elevated serum LDH levels could be used to develop individualized treatment strategies.^219^ A study of a total of 68 studies including 31,857 patients illustrates that LDH overexpression is a predictor to guide individual therapy in solid tumors,^220^ such as testicular cancer,^221^ SCLC,^219^ and gastrointestinal cancer.^222,223^ In conclusion, LDH is a valuable indicator of cancer diagnosis, efficacy evaluation, and recurrence and metastasis.

#### CA72-4

Carbohydrate antigen 72-4 (CA72-4) is a mucin carcinoid embryonic antigen found in liver metastases of breast cancer in 1981 and is highly expressed in human adenocarcinoma.^224^ Enhanced serum CA72-4 levels are effective indicators for the diagnosis of cancers, including gastric cancer, pancreatic cancer, CRC, breast cancer, ovarian cancer, lung cancer, cervical cancer, and endometrial cancer.^225,226^ Notably, CA72-4 exerts diagnostic value in patients with digestive system tumors, especially gastric cancer, with superior sensitivity and specificity.^227^ Studies have demonstrated that the sensitivity and specificity of CA72-4 applied in the diagnosis of gastric cancer alone are 49 and 96%, respectively, which outperforms other tumor biomarkers such as CEA (sensitivity 41%, specificity 93%), CA19-9 (sensitivity 44%, specificity 92%), and CA242 (sensitivity 38%, specificity 97%).^228^ The serum level of CA72-4 is also correlated with the malignant grade of gastric cancer. Thus, CA72-4 is used as the best serum marker for gastric cancer diagnosis in China.^229^ However, CA72-4 also has limitations. It has been uncovered that CA72-4 is highly expressed in normal tissues in addition to tumor tissues such as the endometrium and the colonic transitional mucosa, which results in false positives in patients with atrophic gastritis.^230^ The sensitivity of CA72-4 in the diagnosis of gastric cancer is far from satisfactory.^231^ The combination with other biomarkers may gain increased sensitivity and specificity of CA72-4 in tumor applications. In conclusion, serum CA72-4 is a unique biomarker of gastric cancer for screening, diagnosis, the prediction of metastasis and recurrence, and the evaluation of treatment efficiency.^229^

#### CA125

CA125, a highly glycosylated mucin, is originally discovered in a monoclonal antibody OC125 screening against the ovarian cancer cell line OVCA433.^232,233^ Thus, CA125 has become one of the most important biomarkers for monitoring epithelial ovarian cancer, and its sensitivity in the diagnosis of epithelial ovarian cancer reaches ~70%.^234^ The key role of CA125 in the prognosis of ovarian cancer patients has also been recognized. The Gynecologic Cancer Group (GCIG) has shown that the serum level of CA125 is associated with the progression and recurrence of ovarian cancer. According to the criteria of GCIG, patients with serum CA125 levels within the reference range (<35 U/mL) after surgery or chemotherapy are considered fully effective. While the CA125 level increased to twice of the minimum value (≥70 U/mL), the progression or recurrence is considered.^235^ Moreover, CA125 is also a diagnostic and prognostic biomarker for other nonovarian tumors, such as cervical cancer, endometrial carcinoma,^236^ and gastric cancer.^237^ Of note, ~1% of healthy people and 5% of patients with menstrual or benign diseases such as endometriosis and coronary artery disease have varying degrees of elevated serum CA125 levels.^238–240^

#### CA242

Carbohydrate antigen 242 (CA242), a sugar chain antigen containing sialic acid, is obtained after the immunization of mice with a human CRC cell line COLO 205.^241^ An analysis of serum CA242 levels from 34,680 patients with 27 clinically defined diseases suggests that patients with pancreatic cancer, cervical cancer, and lymphoma have the highest level of serum CA242, which are followed by esophagus cancer, CRC, ovarian cancer, and breast cancer.^242^ Hence, the primary application of CA242 is as a biomarker for CRC and pancreatic cancer.^243^ Serum CA242 has a normal reference value of less than 17 U/mL. The sensitivity for diagnosing pancreatic cancer and CRC is ~70 and 45%, respectively, and the specificity is ~95 and 83%, respectively.^244–246^ As CA242 exhibits a lower sensitivity for diagnosing pancreatic cancer, the combination of CA242 with CEA is a promising strategy for improving diagnosis sensitivity in pancreatic cancer.^247^ In addition, CA242 is also used as a clinical indicator of progression or recurrence during chemotherapy for pancreatic cancer.^241,242^

#### CA15-3

Carbohydrate antigen 15-3 (CA15-3, also known as mucin 1) is a large transmembrane glycoprotein derived from the *MUC1* gene.^248^ It is expressed in normal tissues including the breast, esophagus, stomach, duodenum, pancreas, uterus, prostate, and lung.^248,249^ Notably, CA15-3 is overexpressed in the majority of human cancers, and is thought to be a key biomarker for cancers, especially for indicating cancer metastasis.^250^ The reference value for normal serum CA15-3 levels is less than 28 U/mL.^243^ In breast cancer, serum CA15-3 is used as an auxiliary diagnostic index with a diagnosis sensitivity of 61.5–70% which is higher than that of CEA.^243^ Thus, CA15-3 in combination with CEA is the most popular method for breast cancer diagnosis.^251^ Meanwhile, CA15-3 is also a crucial indicator for the evaluation of postoperative recovery, recurrence, and metastasis of breast cancer.^248^ It is noteworthy that serum CA15-3 is also elevated to varying degrees in benign diseases of the breast, liver, gastrointestinal tract, lung, and other organs, but the positive rate is low.^250^

#### CA27-29

Similar to CA15-3, carbohydrate antigen 27-29 (CA27-29) is a critical epitope for the MUC1 protein.^243^ With a sensitivity of 84% for breast cancer detection, CA27-29 is primarily utilized in breast cancer patients for diagnosis, and efficacy evaluation.^243^ Additionally, it also be used in combination with other markers to increase the specificity of breast cancer diagnosis.^252^ The elevated CA27-29 is also observed in other cancers including CRC, stomach cancer, pancreatic cancer, ovarian cancer, and benign diseases of the breast and liver.^253^

#### CA50

Carbohydrate antigen 50 (CA50) was initially identified as a cancer-specific antigen screened from monoclonal antibodies against CRC cell line COLO 205 in 1983.^254^ It is generally absent in normal tissues, but elevated in multifarious cancers. Patients suffering from pancreatic cancer, lung cancer, and colon cancer exhibit the highest levels of serum CA50. Serum CA50 is quite effective in the diagnosis of pancreatic cancer, with a sensitivity of more than 84%.^255^ Meanwhile, patients suffering from gastric cancer and rectum cancer reveal comparable serum CA50 levels.^256,257^ Similar to other carbohydrate antigens, serum CA50 is also increased in patients with non-neoplasm diseases such as chronic pancreatitis, colitis, cholecystitis, and pneumonia.^256^

#### CA19-9

CA19-9 is initially found in human CRC cell line SW1116 and belongs to the mucin glycoprotein antigen.^258^ It is extensively distributed on the cell membrane of Lewis antigen-positive epithelial cells such as the pancreatic duct, gallbladder, and gastrointestinal tract. CA19-9 is currently the most commonly used and gold-standard biomarker for pancreatic cancer,^259,260^ and holds a median sensitivity of 79% for diagnosis of pancreatic cancer.^261,262^ In addition, CA19-9 has also been used as a biomarker for other cancers, particularly digestive tract cancers.^263^ Other diseases such as liver damage, bile duct obstruction and inflammation, pancreatitis, acute diarrhea, stomach ulcer, and pulmonary fibrosis have also been linked to increased CA19-9 levels.^264–266^ Notably, CA 19-9 is not expressed in cells from patients with Lewis allele deficiencies, and it is necessary to ascertain the patient’s Lewis gene type information when applying CA19-9 as a diagnostic biomarker.^267,268^

#### HE4

Human epididymal protein 4 (HE4), an orotic acid protein, is first identified in distal epididymal epithelial cells.^269^ HE4 is widely expressed in the trachea, salivary gland, lung tissue, etc., and is highly expressed in ovarian cancer, endometrial cancer, and lung cancer. Meanwhile, age and menopausal status are also momentous factors affecting HE4 levels.^270,271^ At present, serum HE4 is primarily used for the diagnosis and recurrence monitoring of ovarian cancer with a sensitivity of 67%. HE4 is also used to evaluate the treatment effect of ovarian cancer.^270,272^ In addition, HE4 is also overexpressed in other non-gynecologic malignancies, including NSCLC, pancreatic cancer, and transitional cell carcinoma.^273^

#### Ferritin

Ferritin is the leading protein that is essential for iron storage and detoxification.^274^ Ferritin is present in numerous normal tissues such as liver, spleen, bone marrow, and body fluids.^274^ Serum ferritin levels are linked to a broad range of conditions. The low serum ferritin concentration indicates iron deficiency, e.g., anemia and diarrhea,^275^ and the high serum ferritin concentration indicates iron overload, e.g., hemochromatosis and hemolytic anemia, or infection and liver disease.^276^ Moreover, ferritin is overexpressed in various cancers, such as HCC, lung cancer, lymphoma, melanoma, and CRC.^277,278^ As indicated by its potential to promote tumor proliferation, angiogenesis, immunosuppression, and tumor drug resistance,^276^ ferritin is valuable in evaluating the progression and prognosis of cancer patients. Nevertheless, a number of factors influence ferritin levels, and ferritin’s limited specificity for tumor detection means that it is not an ideal diagnostic marker for cancers.^279^

#### p2PSA

Prostate-specific antigen precursor (p2PSA) is a precursor that is first secreted in the prostate gland ducts during the production of PSA.^280^ p2PSA is a relatively stable pro-PSA and has certain clinical value in the diagnosis of early prostate cancer. The prostate health index, which forecasts the diagnosis of prostate cancer, is calculated by PSA and p2PSA. Currently, the prostate health index is the strongest predictor of diagnosis at initial biopsy when total PSA levels are between 2.0 and 10 ng/mL in prostate cancer patients, and the prostate health index has been approved by the FDA for early diagnosis and risk grading of prostate cancer.^280,281^

#### HCG

HCG is a polypeptide hormone composed of two noncovalently linked subunits (α and β). The smaller α subunit is the part of follicle-stimulating hormone and luteinizing hormone, while the larger β subunit is unique to HCG.^282,283^ Serum levels of HCG in non-pregnant and menopause women maintain at a low level of 5–10 U/L, and increase dramatically during pregnancy.^284^ Increased serum HCG levels are observed in trophoblastic tumors, ovarian cancer, testicular cancer, breast cancer, lung cancer, HCC, CRC, and kidney cancer. Although HCG level could be employed for monitoring the disease progression, it is too low to be regarded as a diagnostic marker.^282,285^

#### CAM17.1

CAM17.1 is a mucin with high specificity for the digestive system, such as the pancreas, colon, small intestine, and biliary tract.^286^ Several studies revealed that CAM17.1 is particularly overexpressed in pancreatic cancer with a serum cut-off value is 39 U/L.^287^ CAM17.1 has a sensitivity of 86% for the diagnosis of pancreatic cancer, and a higher sensitivity of 89% in patients without jaundice.^286^ These findings suggested that CAM17.1 is a potential biomarker for pancreatic cancer diagnosis, which triggers the need for further study.

#### PIVKA-II

Protein induced by vitamin K absence or antagonist-II (PIVKA-II) is an abnormal prothrombin elevated in the conditions of vitamin K reduction or the presence of vitamin K antagonists.^288^ PIVKA-II is primarily used for the early detection of HCC, with a sensitivity and specificity of 97.5 and 90%, respectively.^289,290^ In other tumors such as gastric cancer and pancreatic cancer, PIVKA-II is also elevated at varying degrees.^291^ In addition to being able to differentiate between other non-malignant conditions such cirrhosis or chronic hepatitis, serum PIVKA-II is more accurate than AFP in the diagnosis of early-stage HCC.^292,293^ It is noteworthy that certain patients with vitamin K deficiency also exhibit elevated PIVKA-II levels.^288^

#### GRP

Gastrin-releasing peptide (GRP), first isolated from gastric nerve fibers by McDonald in 1978, is a gastrointestinal hormone that exits in the normal bronchial epithelial cells, pulmonary fibroblast, central nervous system cells, and neuroendocrine cells.^294^ Significantly, GRP is overexpressed in multiple cancers, including 62% of CRC patients, 59% of pancreatic cancer patients, 60% of prostate cancer patients, 39% of breast cancer patients, and 74% of SCLC patients.^294^ Since GRP has a short half-life and is unstable, it is more appropriate to detect its precursor, pro-GRP.^294^ With a sensitivity of 47 to 86%, serum pro-GRP detection is mainly utilized for the diagnosis, efficacy, and prognosis analysis of SCLC, outperforming NSE.^294,295^ The combined application of pro-GRP and NSE increases the sensitivity of SCLC detection.^296^ In addition, pro-GRP is also elevated in a few other diseases, such as gastritis and acute hepatitis, but the positive rate is generally low.^297^

### Tumor biomarkers derived from tumor tissues

**Fig. 4:**
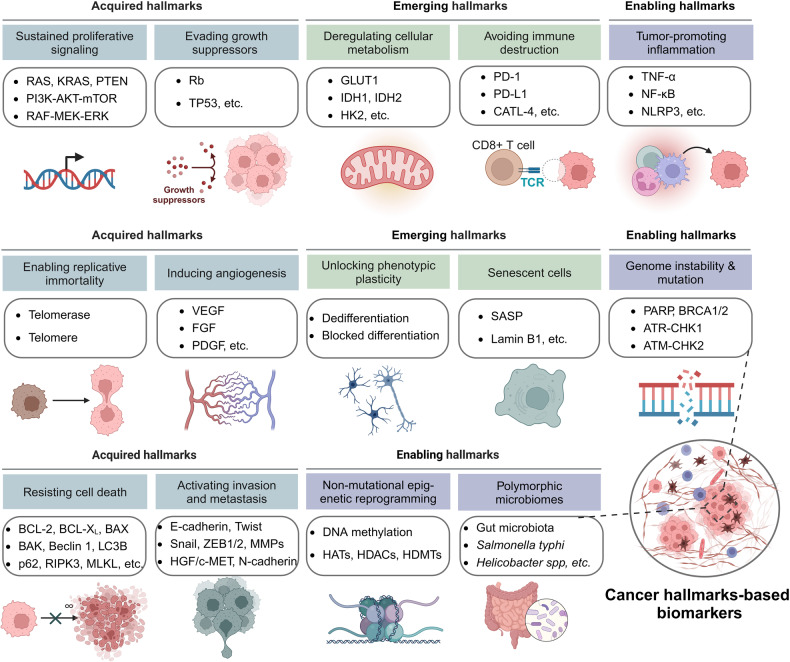
The 14 cancer hallmarks-based biomarkers. Fourteen major characteristics of tumor cells have been proven so far, which have been divided into acquired hallmarks including sustaining proliferative signaling, evading growth suppressors, resisting cell death, enabling replicative immortality, inducing angiogenesis, activating invasion and metastasis, enabling hallmarks including genome instability and mutation, tumor-promoting inflammation, nonmutational epigenetic reprogramming, and polymorphic microbiomes, and emerging hallmarks including deregulating cellular metabolism, avoiding immune destruction, unlocking phenotypic plasticity, and senescent cells. Each of the cancer hallmarks is involved in numerous essential biomarkers that play vital roles in tumor progression

Since the six hallmarks of cancer were proposed in 2000, tumor characteristics are considered to be a set of functional capabilities acquired by human cells during the transition from a normal to a tumor growth state.^298^ To date, tumors have possessed fourteen major characteristics, including sustaining proliferative signaling, evading growth suppressors, enabling replicative immortality, inducing angiogenesis, resisting cell death, activating invasion and metastasis, genome instability, and mutation, tumor-promoting inflammation, deregulating cellular metabolism, avoiding immune destruction, unlocking phenotypic plasticity, nonmutational epigenetic reprogramming, and polymorphic microbiomes, and senescent cells.^298–300^ Herein, we summarize the tumor biomarkers from tumor tissues divided by cancer hallmarks (Fig. 4).

#### Sustaining proliferative signaling

Cancer cells are capable of multiple approaches to acquire the ability to sustain proliferation. Stimulated by growth factors and other proliferative signals, proliferation-related signaling pathways, such as the RAS, the phosphoinositide 3-kinase (PI3K)-protein kinase B (AKT)-mammalian target of rapamycin (mTOR) pathway, and the RAF-mitogen-activated protein kinase (MAPK) kinase (MEK)-extracellular signal-related kinase (ERK) pathway, are activated in tumor cells, which subsequently regulate tumor cell proliferation, migration and invasion, gene transcription, cellular metabolic reprogramming, and tumor microenvironment (TME) remodeling.^301–303^

##### RAS

*RAS* genes, named after the rat sarcoma,^304^ were identified as the transformative factor in the Harvey and Kirsten strains of rat sarcoma viruses^305^ and were identified in the human genome in 1982.^304,305^ RAS proteins belong to a superfamily of GTPases, and three *RAS* genes (*HRAS*, *NRAS*, and *KRAS*) encode four highly homologous RAS proteins: HRAS, NRAS, KRAS4A, and KRAS4B, with the latter two KRAS isoforms arising from alternative splicing.^306,307^

RAS proteins couple cell surface receptors to intracellular effector pathways through binding to GTP or GDP, followed by a cycle between the GDP-bound inactive state (RAS-GDP) and the GTP-bound active state (RAS-GTP). Under physiological conditions, RAS proteins retain an inactive state, and are incapable of interacting with downstream effectors. When activated by upstream receptors, RAS is activated by guanine nucleotide exchange factors (GEFs) which promote GDP to GTP exchange, thereby recruiting diverse downstream effectors such as the RAF-MEK-ERK pathway and the PI3K-AKT-mTOR pathway.^301,308^ RAS activation has been linked to multiple tumor phenotypes, including cell cycle progression, proliferation, metastasis, and apoptosis resistance.^301^ Furthermore, RAS is involved in diverse metabolic processes such as aerobic glycolysis, glutaminolysis, redox homeostasis, and lipid metabolism in tumor cells to support tumor growth.^309^ Importantly, RAS activation remodels the TME,^301^ including the initiation and maintenance of proangiogenesis,^310^ the production of proinflammatory factors,^311^ and immune escape.^301^

RAS mutation is a prominent factor that plays a vital role in tumorigenesis and progression.^312,313^ Approximately 21% of all malignancies have RAS mutations,^308^ which include CRC,^314^ pancreatic ductal adenocarcinoma (PDAC),^315^ lung adenocarcinoma,^316^ and melanoma.^317^

Although the function of RAS in the physiological or pathological states has been thoroughly elucidated in the past decades, numerous unresolved concerns still need to be investigated. For instance, the regulatory relationship between RAS and downstream effectors other than PI3K and MAPK.^318^ To sum up, RAS is a crucial biomarker for tumor diagnosis, prognosis, and treatment.

##### KRAS

KRAS is by far the most frequently amplified and mutated RAS isoform among the three RAS genes, accounting for 85% of all RAS mutations.^319^ KRAS mutations were first identified in 1984 in patients with squamous cell lung cancer.^320^ Notably, KRAS mutations are present in 88% of pancreatic cancer, 50% of CRC, and 32% of lung cancer.^319,321^ The most common mutations in KRAS are G12D, G12V, G12C, G13D, and Q61R, which account for 70% of RAS mutations in cancer patients.^321^ KRAS^G12C^ mutation is the most frequent,^321^ and the G12C mutation alters KRAS conformation and shape by forming binding pockets, leading to increased affinity for GTP and sustained activation of KRAS, ultimately triggering the transduction of downstream oncogenic signaling.^319,321^

KRAS mutations have emerged as biomarkers for the prognosis, diagnosis, and treatment of some tumors, including PDAC,^322^ CRC,^323^ and lung cancer.^324^ A study in a pooled analysis has found that KRAS mutations are independently associated with shorter time to recurrence, survival after relapse, and OS in patients with microsatellite-stable resected stage III CRC.^313^ Patients with the KRAS^G12C^ mutation are related to inferior PFS and OS compared with patients with non-mutated tumors, according to a prognosis analysis in 1239 patients with metastatic CRC.^323^ Moreover, KRAS mutations link to the poor prognosis of patients with PDAC, and KRAS mutation assays provide significant predictive information on tumor progression and recurrence, which are of great value in the diagnosis, prognosis, and treatment of PDAC.^325^ Consistently, PDAC patients with KRAS^G12D^ mutation have shorter survival than all other PDAC patients.^326^ In lung adenocarcinoma patients, the patients with KRAS^G12C^ mutation have worse DFS than patients with nonG12C mutation KRAS or wild-type KRAS.^324^

Mechanistically, KRAS drives tumor development and progression through various signaling pathways. For example, the extensive metabolic reprogramming induced by KRAS mutations, such as glycolysis, glutamine metabolism, lipid metabolism, and nucleotide biosynthesis to facilitate tumorigenesis, has attracted much attention in recent years.^327,328^ KRAS-mutant cells exhibit the upregulation of glucose transporters^329^ and metabolic enzymes involved in the glycolysis,^330,331^ resulting in increased glucose flux in the glycolytic pathway.^329^ KRAS^G12D^ stimulates hexosamine biosynthesis and the pentose phosphate pathway to regulate glucose metabolism in PDAC.^332^ KRAS-mutant cells produce nicotinamide adenine dinucleotide phosphate (NADPH) by promoting glutamine catabolism,^329^ and intracellular fatty acid uptake and oxidation.^333^ Furthermore, KRAS leads to the transcriptional upregulation of MYC and the nonoxidative pentose phosphate pathway gene RPIA through activating MAPK, thereby enhancing nucleotide biosynthesis in PDAC cells.^327^

In summary, KRAS mutations are among the most prevalent drivers of tumorigenesis, and their activation is correlated with tumor progression and poor prognosis.^334,335^ The evidence presented above strongly suggests that KRAS is a crucial tumor biomarker.

##### PI3K-AKT-mTOR

The PI3K-AKT-mTOR pathway plays valuable roles in various cellular processes, such as cell proliferation, angiogenesis, protein translation, and metabolic reprogramming.^302^

In normal cells, growth factor-stimulated PI3K activation leads to the conversion of phosphatidylinositol-3,4-bisphosphate (PIP2) to phosphatidylinositol-3,4,5-trisphosphate (PIP3), followed by the recruitment of AKT and 3-phosphoinositide-dependent kinase 1 to the plasma membrane. Following that, 3-phosphoinositide-dependent kinase 1 phosphorylates and activates AKT, thus phosphorylating the downstream mTOR complex, contributing to cell survival and proliferation.^302,336,337^ The atypical serine/ threonine kinase mTOR consists of rapamycin-sensitive mTOR complex 1 (mTORC1) and rapamycin-insensitive mTORC2.^338^ AKT drives mTORC1 activation either directly by phosphorylating mTORC1 at Ser2448 or indirectly by inhibiting TSC1/TSC2.^302^ mTOR supports cell growth and proliferation by promoting cell cycle,^302^ sensing nutrient signaling^339,340^ by phosphorylating its downstream effectors such as S6K and 4EBP1.^302,341^ The tumor suppressor PTEN is a critical negative regulator of the PI3K signaling pathway.^302^ PETN rapidly metabolizes PIP3 by removing the 3’-phosphate of PIP3, which in turn terminates PI3K signaling.^342^

In cancer cells, the PI3K-AKT-mTOR signaling pathway is abnormally activated via the stimulation of tyrosine kinase growth factor receptors,^343^ the loss of PTEN functions, and the mutations of *PIK3CA*, thereby promoting tumorigenesis in a wide variety of human cancers.^302,342^ The PI3K-AKT-mTOR pathway exerts significant impacts on multiple cancers including lung cancer,^344,345^ ovarian cancer,^302^ breast cancer,^346^ and NPC.^347,348^ The PI3K-AKT-mTOR has been proven to be crucial in ovarian tumorigenesis and drug resistance.^302^ The level of pAKT is a diagnostic biomarker for the treatment of SCLC involving the combination of clinically approved inhibitors against isoform-specific PI3K and mTOR.^345^ In addition, the class I isoform of PI3K, the most well-known PI3K protein, contains four distinct isoforms of catalytic structural domain: p110α (PIK3CA), p110β (PIK3CB), p110γ (PIK3CG), and p110δ (PIK3CD).^343^ pIK3CA and PTEN aberrations lead to the activation of the PI3K-AKT-mTOR pathway.^349,350^ The TCGA database has shown that *PIK3CA* gene mutations occur in a variety of cancers, including 53% of endometrial cancer, 35% of breast cancer, 23% of cervical cancer, 21% of gastric cancer, 20% of head and neck cancer, 20% of CRC, 15% of lung cancer, and 10% of glioblastoma.^343^ PIK3CA mutation, PTEN loss, and pAKT activation are predictive biomarkers for the efficacy of tumor treatment.^350,351^ Moreover, PIK3CA mutations act as diagnostic biomarkers for HR+ and HER2- metastatic breast cancer.^352^ In summary, the PI3K-AKT-mTOR pathway is an essential biomarker pathway for tumor diagnosis, prognosis, and treatment.

##### RAF-MEK-ERK

The RAS-RAF-MEK-ERK pathway participates in the regulation of key processes such as cell proliferation, differentiation, migration, and apoptosis,^303^ which can be activated by growth factors, cytokines, integrins, and chemokine receptors.^303,353^ Active RAS binds to RAF kinase, which results in RAF dimerization and activation.^354,355^ RAF proteins possess three isoforms including BRAF, CRAF, and ARAF which share conserved regions of the regulatory domain and kinase domain,^356^ and among them, ARAF exhibits the lowest kinase activities.^357^ The active RAF dimer phosphorylates and activates MEK1/2 which subsequently phosphorylates and activates ERK1/2, followed by the phosphorylation and activation of downstream effectors and the proliferation of cells.^336^ Various proteins, such as Hsp90,^358^ p50^CDC37^,^359^ and KSR,^360^ are engaged in the regulation of RAF activation.

Abnormalities epically mutations in RAF-MEK-ERK signaling lead to the aberrations of cell proliferation.^361^ A mutation analysis of more than 3000 samples from 12 tumor types has shown that the mutations of RAF-MERK-ERK signaling occur in ~50% of cancers.^362^ In particular, BRAF mutations are widely investigated in cancers.^362^ Studies have revealed that the hyperactivity of the BRAF-MEK-ERK pathway is correlated with worse survival in patients with ER-negative or progesterone receptor-negative breast cancers,^363^ suggesting that the alterations of the RAS-RAF-MEK-ERK pathway could serve as predictive and prognostic biomarkers for breast cancer.^303^ Meanwhile, the aberrations of the RAS-RAF-MEK-ERK pathway can be predictive biomarkers of drug sensitivity in cancer therapies.^303^ In conclusion, the RAF-MEK-ERK signaling cascade functions as a significant biomarker in tumor progression.

##### PTEN

PTEN was discovered in 1997 as a tumor suppressor,^364^ and it was the first phosphatase proven to have tumor suppressive effects.^365,366^ As a phosphoinositide 3-phosphatase, PTEN negatively regulates the PI3K-AKT-mTOR pathway by converting PIP3 to PIP2, thereby hindering the proliferation and survival of tumor cells.^365,367^ Furthermore, PTEN exerts both enzymatic and nonenzymatic effects in cellular epithelial-mesenchymal transition (EMT), migration and invasion, glucose and lipid metabolism, cell cycle, DNA repair, genomic stability, and gene transcription.^365,368^

PTEN function and expression are frequently altered in a variety of cancers.^369^ Accordingly, PTEN acts as a prognostic and predictive biomarker in various cancers including prostate cancer, RCC, PDAC, CRC, breast cancer, endometrial cancer, brain cancers, skin cancers, and hematological malignancies.^370^ Aberration of PTEN is associated with the mutations, downregulation or deletion of the *PTEN* gene, and the abnormal subcellular localization of PTEN protein.^371,372^ PTEN deletion modulates the downstream effector of mTORC1 by regulating 4EBP1 and p70S6 kinase to increase protein synthesis.^372^ Significantly, PTEN deletion is strongly linked to a shorter OS and DFS of cancer patients.^370^ Taken together, PTEN is a significant biomarker for tumor prognosis. The mechanism studies of PTEN activation will be beneficial for the development of antitumor strategies based on the recovery of PTEN function.

#### Evading growth suppressors

In addition to inducing and maintaining growth stimulus signals, tumor cells also eschew powerful programs to evade growth restriction and blockade, which mainly rely on the action of tumor suppressor genes.

##### Rb

*Rb*, the first tumor suppressor gene to be identified, was originally discovered in retinoblastoma children.^373,374^ The alteration of the *Rb* gene or inactivation of the Rb protein is one of the most common events in cancers.^375^ Rb primarily restricts the transcription of cell cycle genes by regulating E2F transcription factors.^376^ Rb proteins are phosphorylated by cyclin-dependent kinases (CDKs),^377^ which lead to Rb functional inactivation, followed by E2F transcriptional activation and cell cycle progression.^378^ Inactivation of Rb causes abnormalities in cell division, defects in cell cycle withdrawal, impaired induction of cellular senescence, and impaired cell cycle checkpoint control.^379^ The function of Rb in tumor cells is disrupted in various ways including Rb gene mutation, Rb protein hydrolysis, Rb-E2F interaction elimination, and the overactivation of CDK.^375^ Consequently, Rb dysregulation acts as a prognostic biomarker in cancers.^380^

##### TP53

*TP53*, often referred to as the “guardian of the genome”, is a gene encoding the p53 tumor suppressor protein.^381^ Numerous studies have shown that p53 plays an integral role in biological processes such as cell cycle arrest, aging, DNA repair, and apoptosis.^382–384^

In human tumors, *TP53* is the most commonly mutated gene with ~50% of tumors carrying the mutations or deletion of *TP53*.^385,386^ In addition to mutations or deletion, tumors may lose the function of p53 due to other mechanisms. For example, overexpression of viral oncoproteins or MDM2 leads to the degradation of p53 protein.^387^ The expression and function loss or gain function of *TP53* are associated with poor prognosis, immune escape, and anticancer drug resistance. Thus, *TP53* can serve as an effective predictive biomarker to evaluate prognosis and monitor therapeutic responses in various cancers.^388^ An analysis of over 29,000 cases from the International Agency for Research on Cancer database revealed that *TP53* mutations are potential prognostic biomarkers, and can be used to bolster the predictive accuracy of the OS and DFS of cancer patients.^389^

#### Enabling replicative immortality

Unlimited proliferation is a critical characteristic of tumor cells.^299^ Normal cells undergo senescence due to their recurrent division cycle, whereas tumor cells are capable of unlimited replication, a phenomenon known as immortalization. The protection of chromosome ends by telomeres is crucial for tumor immortalization.^299^

##### Telomerase

Telomere is a repetitive DNA–protein complex located at the end of chromosome.^390^ Telomeres in normal cells gradually shorten with continuous cell division and eventually fail to protect the end of chromosomal DNA, thus triggering DNA damage, cellular senescence, and apoptosis. Therefore, the length of telomeres is closely related to the cellular lifespan.^299,391^

Telomerase is a DNA polymerase that maintains telomere length by adding telomeric repeat fragments to telomeric DNA ends, thus compensating for the attrition of chromosomal ends in continuous cell division.^390,392^ Telomerase is encoded by the human telomerase reverse transcriptase (hTERT) gene which is the catalytic subunit of telomerase holoenzyme.^390,393^ hTERT is silenced in almost all somatic cells and is significantly re-expressed in ~90% of human cancers by various approaches.^390^ Thus, the large majority of normal human somatic cells lack the telomerase-maintenance mechanism, while a tremendous proportion of cancer cells have a highly active telomerase-maintenance mechanism.^392^ The activation of the telomerase-maintenance mechanism is observed in numerous human cancers, such as breast cancer, CRC, kidney cancer, cervical cancer, liver cancer, lung cancer, pancreatic cancer, prostate cancer, thyroid cancer, and bladder cancer,^394^ which ensure the replicative immortality of cancer cells. In clinical practice, cancer patients with high hTERT levels are along with worse survival than those with low hTERT levels. Moreover, cancer patients with high hTERT levels have a higher risk of disease recurrence and death.^395^ Therefore, telomerase is an independent prognostic biomarker of OS in cancer patients.^395^ Besides, TERT promoter mutations increase the expression of telomerase directly, which contributes to tumorigenesis and is associated with poor OS of cancer patients, suggesting that TERT promoter mutations are prognostic biomarkers for cancers.^396^ Moreover, the nonenzymatic functions of telomeres promote cancer cell proliferation and the resistance of apoptosis,^299^ regulate chromatin structure,^397^ impair DNA damage repair,^397^ and increase antioxidant protein expression,^393^ although the detailed mechanism remains to be elucidated.^299^

#### Inducing angiogenesis

In tumor initiation and progression, the new vascular system can transport nutrients and oxygen, and excrete metabolic waste, which is critical for tumor growth.^299,398^ The transition from prevascular hyperplasia to highly vascularized and progressively outgrowing tumors is known as the “angiogenic switch”. In the early stage of tumor development, the angiogenic switch is highly activated, which in turn sustains the continuous generation of new blood vessels, and causes the transition from dormant hyperplasia to outgrowing vascularized tumor, ultimately promoting rapid proliferation of cancer cells.^299,398,399^ The angiogenic switch, which favors a proangiogenic outcome during tumor angiogenesis, is controlled by the balance between proangiogenic and antiangiogenic factors secreted by tumor cells or TME cells.^398^ Studies have ascertained that angiogenesis significantly contributes to the development of various cancers, including CRC, breast cancer, bladder cancer, RCC, and NSCLC.^400,401^ A large number of angiogenic factors such as vascular endothelial growth factors (VEGFs) have been found to induce the proliferation and differentiation of endothelial cells directly or indirectly.

##### VEGF

VEGF, originally known as vascular permeability factor, was discovered as a tumor secretory factor in 1983 by Senger et al.^402^ In 1989, Ferrara isolated VEGF and renamed it vascular endothelial growth factor.^403^ VEGFs are heparin-binding homodimeric glycoproteins whose family includes VEGF-A (commonly referred to as VEGF), VEGF-B, VEGF-C, VEGF-D, VEGF-E, VEGF-F, and placental growth factor (PlGF).^401,404^ VEGF has been demonstrated to be a potent inducer of angiogenesis,^399^ and is widely expressed in normal adult organs along with two other related endothelial growth factors VEGF-B and VEGF-C, suggesting their necessary roles in tissue angiogenesis and homeostasis.^399^ VEGFs in tumor tissues are extracted not only from tumor cells but also from host cells,^405^ and high levels of VEGFs are found in diverse tumor cells. Interestingly, tumor cells are able to produce VEGFs, but are unable to respond to them due to the absence of VEGF receptors (VEGFRs) on the cell surface.^406^ Multiple factors such as genetic and epigenetic regulation influence the VEGF levels in tumor cells. Among them, epigenetic factors include hypoxia-inducible transcription factors 1α and 2α, low pH, inflammatory cytokines, growth factors, androgens, estrogens, and chemokines.^406,407^ Genetic factors include the activation of oncogenes such as RAS, EGFR, HER2, and the deletion and mutational inactivation of oncogenes such as p53, PTEN, and VHL.^406,408^

VEGFs bind respectively to the three tyrosine kinase receptors (RTKs) VEGFR1–3 with different specificities and affinities and VEGFR2 mediates the main VEGFR signals.^409^ VEGFR-1, the first RTK to be identified as a VEGF receptor,^410^ binds VEGF with a binding affinity ten times higher than that of VEGFR-2, although its ability of signal transduction is quite weak.^406^ VEGFR-1 serves as a decoy receptor to chelate or trap VEGF under some circumstances, thus negatively regulating VEGF activity by preventing VEGF binding to VEGFR-2.^411–414^ The specific mechanism of VEGFR-1 in VEGF-mediated angiogenesis needs to be explored in further detail.^406,415^ In contrast, VEGFR-2 is expressed in almost all endothelial cells, and exerts function through the activation of VEGF, VEGF-B, C, or D.^416^ The binding of VEGF to VEGFR-2 causes receptor dimerization and subsequently activates the intracellular signaling cascades, such as the PI3K-AKT and the RAF-MEK-ERK pathways, which generates neovascular branches required for tumor growth, and ultimately promotes rapid tumor cell proliferation and migration.^406^ VEGFR-3 has similar functions to VEGFR-2, but its action site is mainly in the lymphatic blood vessels.^401,415^ VEGFR-3 is expressed in the lymphatic endothelial cells^415^ and mainly binds to VEGF-C and VEGF-D to induce lymphangiogenesis.^417,418^ In addition, VEGFs interact with the neuropilin receptor family.^415^

VEGF levels are associated with the aggressiveness of tumors.^419^ The plasma VEGF levels in various cancer patients are elevated and negatively correlated with tumor prognosis.^420^ Moreover, VEGF levels are used to predict the efficacy of oral tyrosine kinase inhibitors (TKIs) in cancer patients.^401^ For example, VEGFR inhibitor sorafenib has displayed better therapeutic efficacy against advanced clear cell renal cell carcinoma (ccRCC) patients with high levels of VEGF.^401^ In summary, circulating VEGF and VEGFR-2 have been used as crucial biomarkers for the prediction of prognosis and antiangiogenic drug efficacy.^405,421^

##### FGF

Fibroblast growth factor (FGF) is a secreted glycoprotein^422^ that engages in the regulation of organogenesis,^423^ angiogenesis, and wound repair.^422,424^ FGF binds to the transmembrane FGF receptor (FGFR) on the surface of target cells with high affinity.^422,425^ The mammalian FGFR family consists of four highly conserved transmembrane RTK FGFR1-4, and FGFR5 which has no intracellular tyrosine kinase structural domain but has FGF binding capacity.^422^ FGFR is widely expressed in a broad range of cells, especially endothelial cells.^426^

In tumors, FGF is essential for vascular endothelial integrity, angiogenesis, tumor proliferation, survival, and metastasis.^425,426^ Notably, abnormal FGF signaling accelerates tumor proliferation by promoting tumor angiogenesis.^422^ For example, the elevated level of FGF2 in prostate cancer induces neovascularization to boost tumor growth.^427^ The increased angiogenesis induced by FGF1 amplification in high-grade serous ovarian cancer leads to reduced OS in patients, suggesting that FGF1 is a prognostic biomarker for ovarian cancer.^428^

Furthermore, FGFR is strongly associated with the development of various tumors,^422,429^ such as prostate cancer,^430,431^ lung cancer,^432^ breast cancer,^433^ and pancreatic cancer.^434^ In particular, studies have revealed that FGFR with mutations or amplification functions as driving oncogene to aberrantly activate downstream pathways, resulting in mitogenic, mesenchymal, and antiapoptotic responses in cells.^422^ Somatic mutations of FGFR3 have been observed in more than 30% of bladder cancers.^435,436^ The somatic mutations of FGFR2 have been discovered in 12% of endometrial cancers, and mutant endometrial cancer cell lines are highly sensitive to FGFR TKIs.^437^ Besides, FGFR amplification is also tightly linked to the progression of numerous cancers.^438,439^ Approximately 10% of gastric cancers have shown FGFR2 amplification, which is associated with the poor prognosis of gastric cancer patients.^440^ Amplification of FGFR1 occurs in approximately 10% of breast cancers, especially ER+ type.^441,442^ In brief, FGF and FGFR are vital biomarkers of tumor prognosis and treatment.

##### PDGF

Platelet-derived growth factors (PDGFs), an α-granule component secreted in an autocrine manner during platelet activation,^443^ are critical proangiogenic factors for tumor angiogenesis.^443,444^ The PDGF family contains four different monomeric polypeptide chains: PDGF-A, -B, -C, and -D, which form four homodimers through disulfide bonds (PDGF-AA, -BB, -CC, -DD) and a PDGF-AB heterodimer.^443,445^ The PDGF receptor (PDGFR) consists of RTKs PDGFRα and PDGFRβ. PDGF isoforms trigger different receptor dimerization and phosphorylation by binding to the corresponding PDGFRs, thus activating multiple downstream growth signaling pathways, such as PI3K, MAPK, and JAK/STAT pathways, to promote cancer cell proliferation, migration and invasion, angiogenesis, and drug resistance.^443,446,447^

PDGFs and their receptors are extensively expressed in a number of cancers, such as oral squamous cell carcinoma (OSCC),^448^ skin SCC,^449^ soft tissue sarcomas,^450^ ccRCC,^451^ dermatofibrosarcoma protuberans, gastrointestinal stromal tumors (GIST),^452^ CRC,^453^ breast cancer,^447^ pancreatic cancer,^454^ gastric cancer,^455^ neuroendocrine tumors,^456^ NSCLC, ovarian cancer, and HCC.^443^ High PDGF-A levels correlated independently and inversely with the risk of metastatic relapse in cancer patients.^450^ The level of PDGF-D is associated with advanced tumor stages and the development of bone metastasis.^457,458^ High expression of PDGFR-β is independently linked to prostate cancer recurrence.^445^ In conclusion, PDGFs and PDGFRs are meaningful diagnostic biomarkers.

#### Resisting cell death

Resisting cell death is a significant tumor hallmark that contributes to tumor progression and therapeutic resistance.^299^ Apoptosis that leads to programmed cell death hinders tumorigenesis, and the apoptotic program is considerably reduced in highly aggressive and therapy-resistant tumor cells.^299^ Increasing autophagy activation might inhibit tumorigenesis in parallel with or in concert with apoptosis.^459,460^ Moreover, necrosis also significantly contributes to tumor cell death.^461^ The identification of biomarkers in these processes is useful for tumor diagnosis or prognosis.

##### Apoptosis

Sydney Brenner, Robert Horvitz, and John Sulston shared the 2002 Nobel Prize in Physiology or Medicine for their contributions to the discovery of apoptosis procedure.^462^ Cellular stress, DNA damage, and immune surveillance systems frequently cause apoptosis, a type of cell death that is initiated by the proteolytic cleavage of numerous proteins and the regulation of caspase protease activity.^463^ Apoptosis can be triggered through the intrinsic or mitochondrial pathway and the extrinsic pathway.^464^ The intrinsic pathway is controlled by the B-cell leukemia or lymphoma gene number 2 (BCL-2) family. BCL-2 induces mitochondrial outer membrane permeabilization and the release of multiple proapoptotic factors, followed by the release of cytochrome c from mitochondria to the cytoplasm. Subsequently, the apoptotic peptidase activating factor 1 interacts with cytochrome c, and form the apoptosome that induces the activation of the initiator caspase pro-caspase 9. Later on, the caspase 9 binds to the apoptosome and is cleaved and activated, which subsequently stimulates the activation of initiator caspase 3.^465,466^ This process in which cytochrome c is released from the mitochondria is negatively regulated by antiapoptotic BCL-2 family members such as BCL-2, B-cell lymphoma-extra large (BCL-X_L_), BCL-W, BCL-2-A1, and MCL1.^463^ The membrane permeabilization and the release of cytochrome c into cytoplasm are key processes for triggering apoptosis.^463,467^

The extrinsic apoptotic pathway is initiated through the proapoptotic death receptors which include Fas, the tumor necrosis factor receptor (TNFR) family such as TNFR1, TNFR2, and theTRAIL receptors DR4 and DR5. The proapoptotic death receptors bind to ligands and then trimerize and aggregate within the cell membrane, subsequently recruiting adapter proteins such as FADD, caspase 8 and/or caspase 10 to form the death-inducing signaling complex, which activates the initiator caspase 8, which in turn induces the activation of the effector caspases such as caspase 3, 6, and 7, and apoptosis.^463,467^ Consequently, the potential strategy for cancer therapy is targeting the proapoptotic and antiapoptotic proteins to induce apoptosis.^468^

##### BCL-2/BCL-X_L_

The BCL-2 family proteins have four conserved BCL-2 homology (BH) structural domains (BH1, 2, 3, and 4) which can be divided into three subfamilies based on the homology and function of proteins: the antiapoptotic BCL-2 family members (such as BCL-2 and BCL-X_L_), the multi-BH-domain proapoptotic members, such as the BCL-2-associated X protein (BAX) and the BCL-2 antagonist/killer (BAK), and the proapoptotic “BH3-only” proteins, such as the BCL-2 interacting mediator of cell death (BIM), and PUMA.^469^

BCL-2 was the first identified apoptosis regulator, which was activated by chromosome translocation in human follicular lymphoma oncoprotein.^470^ The BCL-X gene was cloned in 1993,^471^ and the BCL-X_L_ protein, which is localized in the mitochondrion, is the first protein whose three-dimensional structure has been identified in the BCL-2 protein family.^472,473^ The “BH3-only” proteins can be divided into activators and sensitizers.^474,475^ Activators of BH3 proteins, such as BIM, BID, initiate apoptosis by directly inducing BAX and BAK oligomerization and cytochrome c release. However, sensitizer BH3 proteins, such as BAD, and BIK, exert proapoptotic functions by binding to antiapoptotic BCL-2 family members, rather than directly activating BAX or BAK.^475–477^ The interaction of one protein’s BH3 α-helix with a sizable hydrophobic pocket on binding partners regulates the activity of BH3-only proteins,^475^ which initiates apoptosis by activating proapoptotic proteins or by inhibiting antiapoptotic proteins.^478^

BCL-2 can drive oncogenic transformation, hinder apoptosis, and increase tumor cell survival.^479,480^ The high expression of BCL-X_L_ is involved in tumor cell invasion, the maintenance of tumor stem cell phenotype, angiogenesis, and metastasis through inducing apoptosis resistance.^480^ The overexpression of BCL-2 and/or BCL-X_L_ may contribute to tumor progression and the resistance of chemotherapeutic agents in various tumors,^467,475^ including pancreatic cancer,^481^ ovarian cancer,^481^ lung cancer,^481^ prostate cancer,^481^ breast cancer,^482^ neuroblastoma,^483^ CRC,^484^ gastric cancer,^485^ HCC,^469^ chronic lymphocytic leukemia (CLL),^469^ lymphoma,^481^ and multiple myeloma.^481^ Furthermore, BCL-X_L_ can be used as an independent biomarker for the prognosis prediction of CRC patients.^484^ BCL-2 is a prognostic biomarker in TNBC patients. Lower BCL-2 expression level is associated with better outcomes of TNBC patients treated with both adjuvant and neoadjuvant chemotherapy.^486^ In summary, BCL-2 and BCL-X_L_ are essential biomarkers in tumor prognosis and treatment.

##### BAX/BAK

BAX, a cytosolic membrane protein that works as a critical regulator of the apoptotic process, was identified by immunoprecipitation and yeast two-hybrid screening.^480,487^ BAX protein has BH1, BH2, and BH3 structural domains,^480,488^ which is highly homologous with BCL-2.^480^ BAX stimulates apoptosis either by inhibiting BCL-2 and BCL-X_L_ or by directly triggering the apoptotic pathway.^480^ BAX moves from cytoplasm to mitochondria during apoptosis, followed by oligomerization and the formation of pores in the outer mitochondrial membrane, thus facilitating the release of cytochrome c which activates the downstream effector caspases and leads to cell death.^469,489,490^ Downregulation and mutations of BAX are essential for apoptosis resistance,^491^ and BAX acts as a potential prognostic and predictive biomarker in various cancers including gastric cancer, esophageal cancer, and CRC.^492^ The somatic frameshift mutations of the BAX gene highly occur in CRC with the microsatellite mutator phenotype.^493^ BAX mutations are found in ~21% of human hematopoietic malignancies such as ALL.^494^ Reduced BAX expression is a major factor in cisplatin resistance of ovarian cancer cells,^495^ 5-FU resistance of CRC cells,^496^ and zoledronate resistance of lung cancer cells.^497^ The decreased BAX/BCL-2 ratio can be induced by BAX abnormalities, which affects the temozolomide-induced resistance in U87MG cells and paclitaxel-resistant breast cancer cells. Thus, the activation of BAX could be used to promote apoptotic cell death and overcome resistance.^498^

Furthermore, the high BAK expression is correlated with improved OS and PFS in patients with advanced gastric cancer. BAK is a predictive and prognostic biomarker for the therapeutic effect of docetaxel in patients with advanced gastric cancer.^499^ BAX-BAK heterodimer is also used as a pharmacodynamic biomarker of on-target drug action of MCL1 inhibitors.^500^

##### Autophagy

In 1955, Christian de Duve discovered the lysosome,^501^ a key organelle for intracellular degradation, and subsequently introduced the term “autophagy” at the CIBA Foundation Symposium on Lysosomes in 1963.^502^ In 2016, Yoshinori Ohsumi was awarded the 2016 Nobel Prize for Medicine or Physiology for elucidating the mechanism of autophagy, which led to increasing attention to autophagy in health and disease.^460,503^ To date, there are three main types of autophagy: macroautophagy, microautophagy, and chaperone-mediated autophagy. The general term “autophagy” usually means macroautophagy.^504^

Autophagy is a multistep, highly conserved degradation process: the initiation and nucleation of the autophagosome, the expansion, and elongation of the autophagosome membrane, the closure and fusion with the lysosome, and the degradation of products.^460,505^ Briefly, autophagy is triggered by a variety of factors, including nutrient or growth factor deprivation, energy status, hypoxia, ROS, and other stress inducers.^506^ Subsequently, a flat membrane named the phagophore or isolation membrane sequesters cytoplasmic constituents. The elongating phagophore results in complete sequestration and the formation of a double-membraned organelle autophagosome. Then, the autophagosome fuses with the lysosome, and the inner membrane of the autophagosome and the cytoplasm-derived materials it contains are subsequently degraded by the lysosome, resulting in the production of amino acids and lipids which are exported to the cytoplasm for recycling.^504,507,508^

Mechanistically, autophagy-associated (ATG) proteins, a group of evolutionarily conserved proteins, are responsible for this process.^509^ Autophagy begins with the activation of unc-51 like autophagy activating kinase 1 (ULK1) (also known as ATG1) complex which includes ULK1, ULK2, ATG13, FIP200, and ATG101. The ULK1 complex subsequently activates class III PI3K complex which includes VPS15, VPS34, ATG14, Beclin1, UVRAG, and AMBRA1, which mediates vesicle nucleation.^460^ Then, the ATG5-ATG12 complex binds to ATG16 to extend the autophagosomal membrane, and members of the LC3 and GABARAP protein families conjugate with lipid phosphatidylethanolamine (PE) and recruit PE to the membrane. ATG4B binds to ATG7 and then couples with LC3-I and PE to form LC3-II. Eventually, autophagosomes fuse with lysosomes to degrade macromolecules and reuse them. The adapter protein sequestosome-1 (also known as p62) targets autophagosome-specific substrates and LC3-II which are simultaneously degraded.^460,510^

Autophagy is a double-edged sword in cancer. The enhanced autophagic flow in tumor cells accelerates tumor cell growth, while the induction of autophagy can prevent the development of cancer.^459,460^ Therefore, autophagy inhibition and promotion are both promising strategies for cancer therapy, and their application depends on the actual situation.^511^

##### Beclin 1

Beclin 1 was identified as a BCL-2 interaction factor in the yeast two-hybrid screen in 1988.^512,513^ Human Beclin 1, the mammalian orthologue of yeast Atg6, consists of a BCL-2-homology 3 structural domain,^514^ a flexible helical domain,^515^ a coiled coil domain,^516^ and an evolutionarily conserved domain.^514^ Moreover, Beclin 1 contains a leucine-rich nuclear export signal that is essential for its autophagic and tumor suppressor functions.^517^ Beclin 1 is phosphorylated by ULK1 and acts as an integral component of the PI3K complex to localize autophagy proteins to the phagosome. Furthermore, Beclin 1 interacts with and is inhibited by BCL-2/BCL-X_L_ in the BH3 structural domain, which blocks the formation of the Beclin 1-VPS34 complex and inhibits Beclin 1 interacting with UVRAG, thereby inhibiting autophagy. The bind of AMBRA1 to Beclin 1 stabilizes the Beclin 1-VPS34 complex, thus promoting autophagosome formation.^518,519^

Studies have found that Beclin 1 is a prognosis biomarker for various cancers. Reduced expression of Beclin 1 has been observed in brain tumors and cervical cell carcinomas.^520^ The absence of *BECN1* has been found in 40 to 75% of sporadic breast cancer and ovarian cancer,^521^ and 40% of prostate cancer.^522^ Low expression of Beclin 1 is associated with the malignant phenotype and poor prognosis of gastric cancer.^523^ Beclin 1 inhibits the proliferation of human breast cancer cells MCF7 in vitro and in vivo through regulating autophagy.^524^ On the contrary, the elevated Beclin 1 expression is related to distant metastasis and poor prognosis in CRC patients, and reduced survival in CRC patients with 5-FU treatment.^525^ Taken together, Beclin 1 may serve as a valid prognostic indicator and therapeutic target for cancers although further research is needed to determine its specific mechanism in different cancers.^526,527^

##### LC3B

The microtubule-associated protein 1 light chain 3B (LC3B or MAP1LC3B) is a classical autophagy marker, is cleaved by protease at the C-terminus to form free LC3B-I, and LC3B-I binds to PE to form membrane LC3B-II in autophagy occurrence. The process in which LC3B-I converts to LC3B-II is essential for phagophore expansion and the formation of autophagosomes.^528,529^ Thus, LC3B is a marker for the detection of multiple autophagic fluxes.^530^ Accordingly, LC3B-II is one of the most commonly used biomarkers to detect the number of autophagosomes and autophagosome-related structures.^518^

The high LC3B expression is closely associated with the aggressive progression, and poor prognosis of multiple tumors, including gastric cancer,^531^ CRC,^532^ TNBC,^533^ melanoma,^534^ astrocytoma,^535^ esophageal cancer,^536^ and OSCC.^537^ Studies have found that LC3B has the highest expression in TNBC cells in different molecular subtypes of breast cancer,^538^ and its high expression is related to the progression and poor prognosis of TNBC patients.^533^ Moreover, LC3B is closely connected with the vascular invasion and lymph node metastasis of HCC, and is a potential therapeutic target for HCC.^539^ Collectively, LC3B is a meaningful prognostic biomarker in cancer management.^527,534^

##### ULK-1/2

ULK1, a conserved Ser/Thr kinase, plays a pivotal role in autophagy induction.^540^ High expression of ULK-1 is associated with poor prognosis in various tumors, including esophageal SCC,^541^ HCC,^542^ NPC,^543^ prostate cancer,^544^ and CRC.^545^ Studies have found that HCC patients with ULK1 and LC3B overexpression have larger tumors and a higher frequency of lymph node metastasis. The combination of ULK1 and LC3B is an independent predictor of OS and PFS in HCC patients.^546^ After androgen deprivation therapy, prostate cancer patients with high levels of ULK1 have shorter PFS and OS.^544^ In addition, elevated expression of ULK1 has been connected to lymph node metastasis^547^ and functions as a prognostic biomarker in patients with CRC.^545^ Interestingly, low expression of ULK1 is associated with operable breast cancer progression and is a poor prognostic biomarker for patient survival.^548^ In human NPC, ULK1 is also a promising biomarker for the prediction of poor prognosis and treatment response.^543^ Furthermore, ULK2 has been found to be expressed at higher levels in prostate cancer tissues compared with that in normal tissues.^549^ To better determine the prognostic value of ULK1 and ULK2 in different cancer types, comprehensive studies in prospective cohorts are necessary.^530^

##### p62

p62 (also known as sequestosome-1, SQSTM 1) was originally identified as an atypical protein kinase C (aPKC) interacting protein.^550^ p62 consists of several structural domains, including the N-terminal PB1 domain, the ZZ-type zinc finger (ZZ) domain, the tumor necrosis factor receptor-associated factor 6 (TRAF6) binding (TB) domain, the LIR domain, the Kelchlike ECH-associated protein 1 (Keap1)-interacting region (KIR), and the C-terminal UBA domain.^551,552^ Each structural domain of p62 has a different function. The PB1 domain is essential for the formation of homodimeric aggregates that regulate autophagic degradation. Moreover, p62 can interact with other proteins containing the PB1 domain, such as MAPK.^551^ ZZ structural domain is involved in the activation of NF-kB signaling pathway,^553^ and the TB structural domain can interact with TRAF6 which induces protein polyubiquitination.^554^ The LIR structural domain affects autophagosome formation and autophagic degradation by mediating LC3-p62 interactions,^551,555^ and the KIR structural domain activates Nrf2 by binding with Keap1.^556,557^ UBA structural domain is involved in autophagic lysosomal degradation^558^ and apoptosis signaling pathways.^551^

As a marker for autophagic flow detection, p62 accumulation usually represents the inhibition of autophagy.^460,559^ Upregulation or reduced degradation of p62 is associated with tumor progression and anticancer drug resistance.^552^ p62 expression is increased in 60% of lung adenocarcinomas and 90% of lung SCCs.^550^ Numerous studies have shown that high p62 expression is correlated with the aggressiveness and poor prognosis of cancers, including endometrial cancer,^560^ OSCC,^537^ epithelial ovarian cancer,^561^ and NSCLC.^562^ In addition, elevated p62 expression is also correlated with the high-grade, distant metastasis and reduced 5-year survival of breast cancer patients,^563^ especially in patients with TNBC cancer.^564^ In short, p62 is a meaningful prognostic biomarker and a potential target for cancer therapy.^551,552^

##### Necrosis

Necrosis is derived from the Greek “nekros” for corpse.^461^ Necroptosis is a programmed necrotic cell death type in a caspase-independent f manner, induced by TNFR superfamily and mediated by receptor-interacting protein kinase 1 (RIPK1, also known as RIP1), RIPK3 (also known as RIP3), and mixed lineage kinase domain-like (MLKL).^565,566^ Necrosis is caused by numerous stimuli such as cytokines, viral infection, pathogen-associated molecular, T-cell receptors, interferon receptors, Toll-like receptors, cellular metabolism, genotoxic stress, and various anticancer compounds.^565,567^ Common morphological features of necrotic cells include moderate chromatin condensation, cytoplasmic organelle swelling, and the rupture of plasma membrane.^568^ The biochemical characters of necrotic cells include a drop in ATP level, the activation of RIP1, RIP3, and MLKL, the release of damage-associated molecular pattern molecules (e.g., HMGB1), the hyperactivation of poly(ADP-ribose) polymerase 1 (PARP1).^568^ The basic feature that distinguishes necrosis from apoptosis is the rapid loss of cell membrane potential. Cellular energy depletion, membrane lipid damage, and the impairment of steady-state ion pump function lead to loss of membrane potential which in turn leads to cytoplasmic swelling, plasma membrane rupture, and cell lysis, thus promoting necrotic cell death.^461^

Studies have identified that necrosis is an essential predictor for prognosis and treatment response in various tumors, including pancreatic cancer,^569^ RCC,^570^ breast cancer, lung cancer, CRC,^571^ and soft tissue sarcoma.^571,572^ Tumor necrosis is closely associated with cancer-specific survival, OS, RFS, and PFS in patients with RCC, and it can be a prognostic biomarker of patients in clinical practice.^570^ Therefore, the discovery of biomarkers that identify necrosis and molecular mechanisms of necrosis enables the development of necrosis-based antitumor therapies.^569^

##### RIPK3

The serine/threonine kinase RIPK1 is a key regulator of necrosis, and RIPK3 is a downstream regulator of RIPK1.^573,574^ The RIPK1-RIPK3-MLKL complex, also known as the “necrosome”, mediates upstream cell death receptors and downstream signaling.^565^ Necrosome is a multiprotein complex that contributes to TNF-induced cell death.^575,576^ Necrotic cells trigger caspase 8 inactivation and activate RIPK1 and RIPK3, followed by autophosphorylation and cross-phosphorylation between RIPK1 and RIPK3 to form necrosome. Then, MLKL is phosphorylated, followed by oligomerizing and translocating to the plasma membrane and stimulating the necroptosis.^577,578^

The RIPK3 expression is significantly reduced in AML patients,^579,580^ which is consistent with the high methylation level near the transcriptional start site of RIPK3.^581^ RIPK3 deficiency promotes leukemogenesis by enhancing the accumulation of leukemia-initiating cells, and hinders myeloid differentiation through reducing cell death and IL-1β production.^579,580^ In addition, RIPK3 expression plays an important role in solid tumors. RIPK3 has been discovered to be downregulated in various cancer cells, including breast cancer,^581^ melanoma,^582^ lung cancer,^583^ and CRC.^584^ RIPK3 is downregulated in human CRC tissues compared with normal tissues,^585,586^ and the deletion of RIPK3 accelerates colorectal tumorigenesis in mice through sustained inflammation.^577,585^ Consistent with the above observations, low RIPK3 levels are strongly correlated with poor prognosis in patients with CRC^586^ and breast cancer.^581^ On the contrary, the expression of RIPK3 is elevated in several other tumors, such as serous ovarian cancer,^587^ pancreatic cancer,^588^ and colitis-associated cancer and colon cancer.^589^ RIPK3 promotes colitis-associated CRC through tumor cell proliferation and CXCL1-induced immunosuppression, and RIPK3 deficiency significantly reduces colitis-associated CRC development in mice.^577,589^ In conclusion, RIPK3 is a potential prognostic biomarker for tumors, although its role needs to be analyzed on a case-by-case basis.

##### MLKL

MLKL is a key factor in necroptosis execution,^574,576,590^ and a vital determinant of treatment response and poor prognosis in cancer patients.^579,591^ The low expression level of MLKL is significantly associated with lower OS in gastric cancer,^592^ ovarian cancer,^593^ cervical SCC,^594^ colon cancer,^577,595^ and pancreatic cancer.^591^ Moreover, in resected PADC patients receiving adjuvant chemotherapy, the low expression level of MLKL is related to decreased RFS. Thus, MLKL has become a prognostic biomarker for patients with early-stage resected PDAC.^591^ However, high levels of MLKL are tied to poor prognosis in patients with colon and esophageal cancers.^596^ The mRNA expression level of MLKL in gastric cancer tissues is significantly higher than that in normal tissues.^592^ The possible reason for this difference is that some cancer cells activate necrosis to modulate the immune system, and the exact mechanism needs to be further investigated.^577^ In short, MLKL is a potential prognostic biomarker for cancer patients.

#### Activating invasion and metastasis

**Fig. 5:**
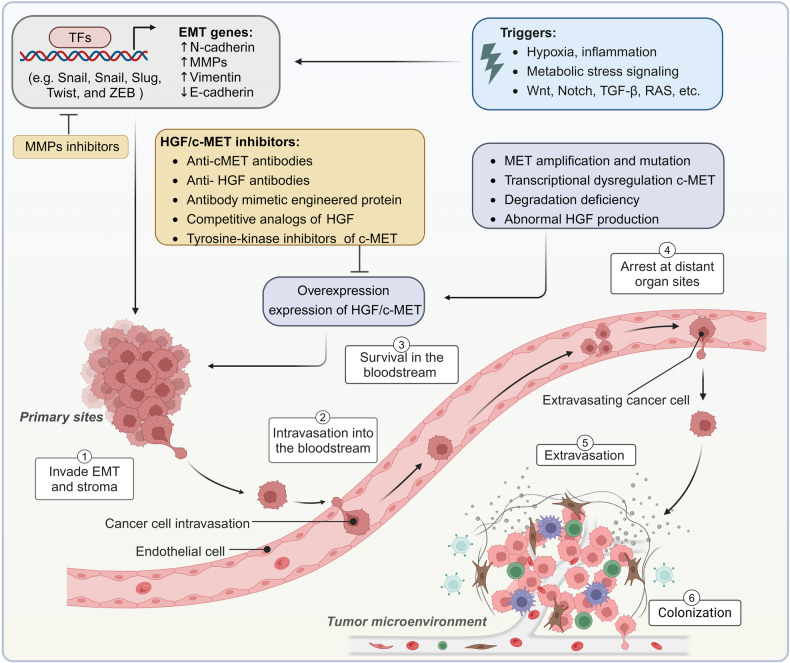
The cancer invasion and metastasis and its targeted therapy. The tumor metastasis process consists of multiple steps. Initially, tumor cells invade the surrounding stroma and extracellular matrix from the primary tumor site, and then intravasate into the bloodstream or the lymphatics. Subsequently, tumor cells arrest in the circulation and arrive at distant organ sites, followed by extravasating and invading the parenchyma of distant tissues. Finally, tumor cells adapt to the new microenvironment and grow to form metastatic colonization. EMT is the basic embryonic developmental process that transforms polarized non-motile epithelial cells into motile and invasive mesenchymal cells. Multiple cellular stress conditions including hypoxia, inflammation, metabolic stress, and signaling cascades, can induce the expression of EMT transcription factors and prompt tumor metastasis. Meanwhile, MET amplification and mutation, the transcriptional dysregulation of c-MET, degradation deficiency, and abnormal HGF production result in the abnormal expression of HGF/c-MET and tumor progression. Various inhibitors including MMP inhibitors and HGF/c-MET inhibitors have been developed and emerging as promising tools in the suppression of tumor metastasis. c-MET mesenchymal-epithelial transition factor, EMT epithelial-mesenchymal transition, HGF hepatocyte growth factor, MMPs matrix metalloproteinases

Tumor metastasis is a process of transferring tumor cells from the primary lesion tumor to distant tissues and organ cascades.^597,598^ Tumor metastasis is divided into multiple steps: (1) tumor cells invade the extracellular matrix (ECM) and the surrounding stroma; (2) tumor cells enter into the bloodstream directly or the lymphatics; (3) tumor cells survive in the circulation; (4) tumor cells arrest in the circulation and arrive at distant organ sites; (5)tumor cells extravasate and invade into the parenchyma of distant tissues; (6) survival in the microenvironment and grow to form metastatic colonization^599–601^ (Fig. 5). In 1889, Stephen Paget vividly compared tumor metastasis to fertile “seeds” (tumor cells) falling on “congenial soil” (the metastatic microenvironment).^602,603^ Many changes occur in “seeds” during the metastasis process, including proteolytic degradation of basement membranes and ECM, changes in tumor cell adherence to cells and the ECM, and physical motility of tumor cells.^599,604^ Meanwhile, homeostasis of “soil” is also altered before tumor cells arrival by modulating the cellular composition, immune status, blood supply, and ECM of the metastatic site to create a microenvironment conducive to tumor cell colonization.^605^

Activated invasion and metastasis have been recognized as one of tumor hallmarks^299,600^ and a major cause of death in patients with solid tumors.^606^ Predicting tumor metastasis facilitates the implementation of personalized therapy in the clinical treatment of tumors, leading to better outcomes for cancer patients. Thus, identifying metastatic biomarkers helps to detect initial tumor metastasis or recurrence in clinical practice, thus improving the potential treatment and management strategy for cancer patients.

##### E-cadherin

Cadherins are a superfamily of at least 80 specific types of adhesion molecules characterized by the ability to form calcium-dependent intercellular homophilic bonds,^607^ which are involved in the regulation of tumor cell recognition, tumor suppression, and tissue morphogenesis.^608^ Common family members include Epithelial (E)-cadherin, Neuronal (N)-cadherin, and Placental (P)-cadherin.^609^ E-cadherin, a homophilic cell-cell adhesion molecule,^610^ is a type I cadherin expressed in epithelial cells. E-cadherin is the first member of the cadherin superfamily to be identified.^607^ The human E-cadherin gene (*CDH1*) is located on chromosome 16q22.1. The structure of mature E-cadherin consists of three parts: a highly conserved carboxyterminal cytodomain that is identical in all cadherin family members, a single-pass transmembrane domain, and an extracellular domain that consists of five cadherin-motif subdomains with putative calcium-binding sites.^610,611^ E-cadherin mediates cell adhesion through calcium-dependent trans-homodimeric interactions of its EC1 structure with the EC1 domain of adjacent cells, while the cytoplasmic part interacts with adherens junctions-related molecules such as β-catenin.^612^ E-cadherin plays a significant role in normal embryonic development, organ morphogenesis, and tissue formation by regulating proliferation, migration, or maintaining epithelial cell polarity.^613^ Thus, E-cadherin is a biomarker of the epithelial cell layer.^613^

The embryonic program EMT is of great importance in the progress of epithelial-derived tumors from benign lesions to invasive carcinomas and metastases.^613,614^ This process is accompanied by changes in cadherin expression^599,615^: from E-cadherin which promotes tumor adhesion and blocks invasion, to N-cadherin which is expressed in mesenchymal cells to promote tumor cell invasion,^599^ and E-cadherin dysfunction is an EMT landmark in this process.^613^ The causes of abnormal E-cadherin in tumor cells include reduced or absent E-cadherin expression, mutations or reduced transcription of E-cadherin genes, abnormal redistribution of E-cadherin within cells, the shedding of E-cadherin from the cell surface, and competition with other proteins for binding.^610^ In addition, E-cadherin is an important tumor growth suppressor,^616^ and inhibits tumor cell growth by upregulating p27-induced cell cycle arrest. Inhibition of E-cadherin leads to a decrease in cell adhesion, which promotes tumor metastasis.^610^

Several investigations have demonstrated the critical role that E-cadherin plays in tumor progression. E-cadherin is closely connected to pathological and clinical characteristics of tumor patients, such as the degree of differentiation, aggressiveness, venous permeation, peritoneal seeding, infiltrative growth, liver and bone metastasis, lymph node metastasis, tumor staging, and poor prognosis.^609,617–619^ The deletion or downregulation of E-cadherin promotes tumor invasion, infiltrative growth, and dedifferentiation.^610,616^ Thus, E-cadherin can be utilized as a prognostic biomarker of tumor metastasis for multiple tumors,^609^ including CRC,^620^ gastric cancer,^621^ pancreatic cancer,^622^ esophageal cancer,^623^ liver cancer,^624^ lung cancer,^625^ bladder cancer,^626^ prostate cancer,^627,628^ breast cancer,^629^ endometrial cancer,^630^ ovarian cancer,^631^ thyroid cancer,^632^ and HNSCC.^633^

##### EMT transcription factors

EMT is the basic embryonic developmental process that transforms polarized non-motile epithelial cells into motile and invasive mesenchymal cells.^634,635^ In tumor cells, EMT promotes tumor cell invasion and metastasis, induces cancer stem cell (CSC) stemness, chemoresistance, immune evasion, and cellular metabolic reprogramming,^636,637^ and inhibits senescence.^638^

EMT is regulated by EMT transcription factors which are classified according to their direct or indirect repression of E-cadherin.^634^ The direct repressors include zinc finger proteins of the Sail superfamily Snail1 (also known as Snail), Snai2 (Slug), and Snai3 (Smuc), zinc finger E-box binding protein (ZEB) family members ZEB1 and ZEB2. The indirect repressors including the basic helix-loop-helix proteins Twist1 and Twist2.^634,639^ Tumor cell stress conditions, such as hypoxia, inflammation, or metabolic stress, stimulate signaling cascades, such as Wnt, Notch, TGF-β, and RAS, and induce the expression of EMT transcription factors Snail, Slug, Twist, and ZEB.^634,640^ Then, EMT transcription factors induce downstream effects of EMT by a series of processes including regulating epithelial marker-related or mesenchymal marker genes, activating matrix metalloproteinases (MMPs) expression or interacting with epigenetic regulators to promote oncogenic transformation, modulating CSCs, generating chemoresistance, and increasing tumor angiogenesis, and ultimately promoting tumor cell motility and metastasis.^634,638,641^ In addition, EMT transcription factors also regulate tumor prosurvival phenotypes, such as participating in tumor cell DNA repair, the evasion of senescence and apoptosis, and immune evasion, providing survival advantages for tumor cells under various stress conditions.^642^

A significant enrichment analysis of 244 differentially expressed EMT-related genes in CRC has revealed that EMT-related signaling pathway genes are highly related to the prognosis prediction of CRC patients, where higher risk scores indicate poor prognosis.^643^ In conclusion, EMT transcription factors have been considered as prognostic biomarkers for tumor aggressiveness and metastasis in clinical practice.^644–646^

##### Twist

Twsit1 and Twsit2 are highly conserved basic helix-loop-helix transcription factors,^638,647^ which are pivotal regulators of embryonic morphogenesis.^648^ *Twist* is expressed in mesodermal and ectodermal-derived tissues, and it has been found that Twsit1 and Twsit2 which are structurally homologous are overexpressed in multiple human cancers.^647,648^ Twsit1 overexpression has been confirmed to be strongly associated with aggressiveness and metastasis in cancer patients, including sarcoma, glioma, melanoma, ESCC,^649^ neuroblastoma,^650^ cervical cancer,^651^ RCC,^652^ and hematological malignancies including AML, chronic myeloid leukemia (CML), ALL, CLL, lymphomas.^653^ In CML patients, the increased expression of Twist is related to tumor progression, tumor staging, and drug resistance, and Twist can be applied as a biomarker to assess MRD.^653^ Inhibition of Twist expression has been found to impair the high metastasis of breast cancer cells from the mammary gland to the lung.^648^ Collectively, Twist is a meaningful biomarker for tumor prognosis and metastasis.^647^

##### Snail

Snail, the first member of the snail superfamily, was first described in *Drosophila melanogaster*,^654,655^ and is essential for cellular mesoderm formation.^654^ The three members (Snail, Snai2, and Snai3) of the Snail family share a similar structure: a highly conserved C-terminal domain containing four to six C2H2-type zinc finger.^656^ In cancer cells, Snail functions as a transcriptional repressor by binding to the E-box motif (CAGGTG) of Snail-related genes with its C-terminal structural domain, thus inhibiting the transcription of target genes.^654,656^ For example, Snail downregulates E-cadherin expression and thereby induces EMT and basal-like phenotype conversion.^636^ The overexpression of Snail is associated with poor prognosis in patients with breast cancer,^657^ CRC,^658^ and liver cancer.^659^ Snail expression is significantly higher in the high-stage, high-grade, and significant lymphovascular invasion patients with upper urinary tract urothelial carcinoma.^660^ Slug, a member of the Snail family, also has a striking impact on EMT. Slug expression is an independent prognostic biomarker for poor survival in CRC^661^ and esophageal SCC patients.^662^

##### ZEB1/2

ZEB1 (also known as Zfhx1a and Zfhep) and ZEB2 (also known as SIP1 and Zfhx1b),^663^ members of the ZEB transcription factor family,^638^ which are encoded by the *ZFHX1a* and *ZFHX1b* genes.^664^ Both ZEB1 and ZEB2 possess two separated clusters of C2H2-type zinc fingers which bind to paired E-box promoter elements.^664^ ZEB1 is a key regulator of tumor cell plasticity and metastasis.^665^ Mechanically, ZEB1 binds directly to the E-box in the promoter of the *CDH1* gene which encodes E-cadherin, blocking *CDH1* transcription and inducing EMT.^666^ ZEB1 overexpression is strongly associated with highly aggressive precursor lesions and poor prognosis of pancreatic cancers.^665,667,668^ ZEB1 deficiency reduces stemness, tumorigenic, and colonization capacities in CSCs of pancreatic cancer, thereby inhibiting the formation of undifferentiated high-grade cancers, invasion, and metastasis.^665^ ZEB1 overexpression serves as a significantly independent adverse prognostic factor for RFS and OS in metaplastic breast cancer.^669^ The knockdown of ZEB1 in human breast cancer cells results in approximately 230 gene changes, most of which are related to epithelial differentiation and intercellular adhesion.^666^ Moreover, aberrant expression of ZEB1 is associated with multiple tumor progression and metastasis, including uterine cancer, osteosarcoma, lung cancer, liver cancer, and gastric cancer, which reveals the importance of ZEB1 in EMT induction and tumor development.^666^

ZEB2 is a DNA-binding transcriptional repressor consisting of multiple functional domains which interact with various transcriptional effectors.^670^ ZEB2 is proven to be highly expressed in human cancer cell lines lacking E-cadherin protein. Overexpression of ZEB2 blocks E-cadherin protein-mediated intercellular adhesion and promotes tumor cell metastasis.^671^ ZEB2 promotes the migration and invasion of breast cancer,^672^ bladder cancer, ovarian cancer, stomach cancer, CRC,^673^ OSCC,^674^ and pancreatic cancer.^667^

##### HGF/c-MET

c-MET, also known as RTK Met, was first identified as a proto-oncogene in the 1980s.^675,676^ c-MET is a disulfide-linked heterodimer composed of an extracellular α-subunit and a single-pass transmembrane β-subunit, which is translated and cleaved form pro-c-MET, a 170 kDa single-stranded precursor protein.^677,678^ The β-subunit of c-MET is involved in the regulation of kinase activity and effector signaling by forming extracellular and partially intracellular structural domains.^677^ Hepatocyte growth factor (HGF, also known as scatter factor) is the only known c-MET ligand,^677^ which is a 90 kDa heterodimer composed of an α chain and a β chain.^677^ HGF consists of six structural domain groups: amino-terminal domain (N), four kringle domains (K1–K4), and a serine proteinase homology (SPH) domain,^675^ of which the N-terminal and the first kringle region are c-MET high-affinity binding sites. HGF induces c-MET dimerization and phosphorylates c-MET residues Y1349/1356, subsequently activating various downstream signaling pathways including the ERK1/2, p38/MAPK, and PI3K-AKT, ultimately promoting cell proliferation and survival.^675,677^

Under normal physiological conditions, HGF/c-MET is involved in cellular processes such as embryogenesis, angiogenesis, wound healing, and organ regeneration. While abnormal expression of HGF/c-MET in tumor cells including MET amplification and mutation, the transcriptional dysregulation of c-MET, degradation deficiency, and abnormal HGF production are closely related to tumor progression.^677,679^ c-MET activation enhances tumorigenicity, invasion, and metastasis.^680,681^ High expression of HGF/c-MET is revealed in various cancers and is closely associated with the poor prognosis of cancer patients.^675,682,683^ For example, c-MET locus amplification occurs in patients with gastrointestinal cancers such as gastric cancer, metastatic CRC, gastroesophageal cancer, and esophageal adenocarcinoma.^677,679^ c-MET mRNA and protein levels are significantly higher in liver metastasis of CRC than in primary CRC, and its expression is positively correlated with tumor stage in CRC liver metastasis.^684^ Besides gastrointestinal cancers, c-MET mutations are found in papillary renal cancer,^685^ ovarian cancer,^685^ SCLC,^686^ HNSCC,^687^ and childhood HCC.^679,685^ Elevated HGF levels are found in various cancers including head and neck cancer,^688^ cervical cancer,^689^ HCC,^690^ and lung cancer,^691^ and are associated with poor prognosis. HGF promotes HCC migration and invasion, and is positively correlated with HCC metastasis.^692^ HGF has been observed to be an independent blood-based predictive biomarker and primary diagnostic marker in ovarian cancer patients.^693^ Moreover, HGF/c-MET can be used as a prognostic biomarker in various hematologic tumors, such as B-cell lymphoma, T and natural killer (NK) cell lymphoma, and Hodgkin lymphoma.^694^

Furthermore, c-MET activation mediates resistance to TKIs, chemotherapy, cetuximab, and radiotherapy in CRC patients.^677^ c-MET mediates radio-resistance by increasing cell motility and inhibiting apoptosis through autocrine and paracrine signaling.^695^ HGF co-amplification leads to clinical resistance in MET-amplified esophagogastric cancer.^696^ In conclusion, c-MET/HGF overexpression is an independent biomarker of poor prognosis and drug resistance in patients with various hematologic and solid tumors.

##### N-cadherin

N-cadherin, also known as cadherin 2 or CDH2,^697^ was identified in the 1980s.^698^ N-cadherin is a single-pass transmembrane calcium-binding glycoprotein that mediates intercellular adhesion,^699,700^ and consists of five extracellular substructural domains (EC1-EC5).^701^ In addition to expression in normal cells such as neuronal cells, osteoblasts, stromal cells, and endothelial cells.^702^ Studies have found that N-cadherin is highly expressed in various tumors including melanoma,^703^ neuroblastoma,^704^ breast cancer,^705^ urothelial cancer,^702^ ovarian cancer,^706^ and multiple myeloma.^701^ Abnormal expression of N-cadherin promotes tumor cell survival, proliferation, invasion, and metastasis by regulating signaling pathways, such as fibroblast growth factor receptor signaling, canonical Wnt signaling,^701,702^ and signalings involved in neovascularization and vascular stability regulation.^701,707^ In addition, N-cadherin exhibits great importance in hematological malignancies, such as leukemia and multiple myeloma,^702^ and is closely associated with poor prognosis in multiple myeloma.^708^ The N-cadherin antagonist ADH-1 induces cell apoptosis in various tumors including neuroblastoma,^704^ multiple myeloma,^709^ and pancreatic cancer,^710^ and improves the efficacy of tumor-infiltrating lymphocyte therapies.^711^ Blocking N-cadherin effectively inhibits prostate cancer invasion, metastasis, and castration resistance, which has become an important therapeutic target and biomarker for prostate cancer.^712^

##### MMPs

MMPs, also called matrixins, are highly conserved zinc-dependent endopeptidases belonging to the metzincin superfamily.^713,714^ MMP1, the first matrix metalloproteinase, was discovered in 1962 in the tadpole tail, which exerted the ability to degrade collagen.^715^ The members of the MMP family can be divided into six major groups: the astacins, the adamalysins (a proteinase with a disintegrin and metalloproteinases, ADAMs, the ADAMs with thrombospondin motif, the pappalysins, the serralysins, and the MMPs.^714–716^ Most catalytic domains of MMPs are highly homologous and basically consist of four structural domains. However, differences between each MMP still exit including substrate specificity, cellular and tissue localization, membrane binding, and regulation.^713,717^ MMPs consisting of 23 members with different structural domains in humans are widely expressed in various organs and tissues.^713^

The ECM is a fundamental component of body tissues and organs, which maintains tissue integrity by homeostatic balance between ECM production and its degradation.^715^ MMPs are proteolytic enzymes capable of degrading the basement membrane and the most of ECM components, thus remodeling the ECM.^604^ In addition, MMPs can also act as extracellular processing enzymes to regulate protein functions, as well as participate in various homeostatic regulations in tumor cells, such as immunity, angiogenesis, cell adhesion, cell proliferation, apoptosis, and EMT.^713,718^

The upregulation of MMPs has been observed in different tumors, such as breast cancer,^719^ CRC,^720^ gastric cancer,^721^ esophageal cancer,^722^ urinary bladder cancer,^718^ and lung cancer,^723^ which increases tumor metastasis and promotes cell invasion.^604,724^ In particular, MMP-9 is critical in cancer cell invasion and metastasis, and has been demonstrated to be a key biomarker in different cancers including NSCLC,^725^ cervical cancer,^726^ gastric cancer,^727^ ovarian cancer,^728^ breast cancer,^729^ osteosarcoma,^730^ and pancreatic cancer.^731^ The expression of MMP1, MMP2, and MMP16 are positively correlated with OS and DFS in patients with uveal melanoma.^732^ Collectively, MMPs can be potential biomarkers in various cancers.

#### Genome instability and mutation

DNA is a relatively stable organic molecule and genomic maintenance systems monitor and resolve damaged DNA, thus ensuring low mutation frequency within cells. During tumor development, cancer cells induce the accelerated accumulation of mutations by compromising genomic integrity or forcing genetically damaged cells to senescence or undergo apoptosis.^299^ DNA damage response (DDR) coordinates DNA repair by regulating cell cycle checkpoints and other global cellular responses. Genome instability and mutation caused by DDR defects are important hallmarks of cancer.^733^

##### PARP

DNA single-strand break (SSB) or single-strand nick are primarily recognized by PARP1 or PARP2, which catalyze the formation of poly (ADP-ribose) (PAR) chains on themselves and neighboring target proteins.^733^ PARP1 and its activity in poly(ADP-ribosyl)ation (PARylation) at SSBs recruit the scaffold protein XRCC1 which drives DNA ligase 3 (LIG3) and accessory repair factors to rejoin disruptions. The poly(ADP-ribose) polymerase (PARP) plays an important role in many cancer types, including ovarian cancer, breast cancer, pancreatic cancer, and prostate cancer.^733^ Furthermore, PAR chains are rapidly degraded by PAR glycohydrolase which restores PARP and PARylated proteins to a de-(ADP-ribosylated) state to promote SSB repair. As PARylation is a highly dynamic and transient process, the inhibition of both PAR glycohydrolase and PARP could reduce the repair efficiency of SSBs, exhibiting their anticancer efficiency.^733^ Especially, PARP inhibitors have been demonstrated to block the SSB repair pathway and trigger synthetic lethality in cancers with homologous recombination (HR) deficiency which results in impaired DNA double-strand breaks (DSB) repair.^734,735^

##### BRCA1/2

In addition to SSB, DSB exerts a vital role in genome integrity. There are two major DSB repair pathways in human cells: the nonhomologous end joining pathway and the HR pathway.^733^ The HR pathway uses the homologous DNA molecule (usually the sister chromatid) as the repair template. HR is initiated when nuclease digests double-stranded DNA ends at DSB sites to produce ssDNA overhangs. Immediately afterward, BRCA1 facilitates the recruitment of BRCA2 to DSB sites through interaction with PALB2, which loads RAD51 directly onto ssDNA ends. Nucleoprotein filaments are formed on ssDNA by RAD51, which subsequently promotes strand invasion and displacement loop (D-loop) ss formation. Finally, the invasion strand is replaced and strand annealing contributes to the HR completion.^733^

BRCA1/2 maintain genomic integrity after DNA damage by promoting accurate DNA repair via the HR pathway.^733^ BRCA1/2 regulate DNA replication by preventing nuclease degradation of nascent DNA and promoting the HR repair of broken replication forks to regulate DNA replication.^733,736,737^ The loss of BRCA1/2 function leads to the accumulation of DNA damage and genomic alterations including insertions, deletions, and chromosomal rearrangements, ultimately damaging genomic integrity and promoting tumorigenesis.^733^ The overexpression of BRCA1/2 is significantly associated with worse OS and clinicopathological characteristics in breast cancer.^738^ High expression of cytoplasmic BRCA1 and BRCA2 is significantly associated with favorable OS in digestive cancers, whereas BRCA1 nuclear expression usually predicts poor outcomes. Thus, BRCA1/2 could be used as clinicopathological biomarkers to evaluate the prognosis of digestive system cancers.^739^ Moreover, BRCA1/2 mutations are closely related to the progression of multiple cancers, including breast cancer, ovarian cancer, prostate cancer, and pancreatic cancer.^733,740^ BRCA1/2-deficient cells are highly sensitive to PARP inhibition,^741^ which is due to inhibition of PARP-dependent SSB repair resulting in the accumulation of DNA lesions (SSBs and DSBs) during replication.^733^ In conclusion, BRCA1/2 serves as a biomarker for prognosis and treatment response in cancer.

##### ATR-CHK1/ ATM-CHK2

Ataxia telangiectasia mutated (ATM) is a kinase responsible for orchestrating cellular responses to DSB and replication stress, including DNA repair, checkpoint activation, apoptosis, senescence, chromatin structural change, and transcription.^742^ Ataxia telangiectasia and Rad3-related protein (ATR), an essential regulator of the cellular replication stress response, is involved in cell-cycle arrest, inhibiting the beginning of replication origins, regulating global fork speed, and promoting fork stabilization.^743^ ATM and ATR respond to DNA damage by phosphorylating hundreds of substrates.^744^ The checkpoint kinase 1 (CHK1) and checkpoint kinase 2 (CHK2) are the major substrates downstream of ATR and ATM, respectively, and are responsible for downregulating the activity of CDKs, thereby preventing cell cycle progression under stress. ATM is recruited to DSB sites and promotes histone H2AX phosphorylation. Phosphorylated H2AX in turn recruits the mediator of DNA damage protein MDC1, and subsequent MDC1 phosphorylation by ATM leads to recruitment of DNA damage mediator proteins 53BP1 and BRCA1, thereby promoting DSB repair.^733^

ATM is frequently mutated or inactivated in a variety of tumors, including lung cancer, breast cancer, brain cancer,^745^ and pancreatic cancer.^746^ Endometrial cancer patients with ATM mutations exhibit a higher tumor mutational burden, a higher neoantigen load, and increased expression levels of immune checkpoints. Thus, ATM mutations can act as an independent prognostic factor and a potential biomarker for immune checkpoint therapy in endometrial cancer.^747^ Moreover, ATM mutations are independently associated with longer OS in patients with metastatic CRC.^748^ ATM deficiency also renders cancer cells sensitive to topoisomerase I inhibitors or PARP inhibitors. PARP and topoisomerase I inhibitors lead to single-ended DSB, while ATM inactivation delays DNA damage repair, leading to toxic chromosome fusions.^733^

#### Tumor-promoting inflammation

As one of the tumor hallmarks,^299^ persistent inflammation plays an essential role in a variety of human cancers by manipulating cancer development, angiogenesis, malignant transformation, invasion and migration, immune surveillance, and response to therapy.^749,750^ Inflammation-related regulators, including tumor necrosis factor-α (TNF-α), nuclear factor-κB (NF-κB), and Nod-like receptor protein 3 (NLRP3), are potential tumor prognostic biomarkers.

##### TNF-α

TNF-α, a vital member of the multifunctional TNF superfamily, is a 17 kDa type II transmembrane protein that was first isolated from the serum of mice infected with Bacillus Calmette-Guérin and endotoxin by E. A. Carswell in 1975.^751,752^ As a key molecule mediating the tumor-promoting inflammatory process, TNF-α drives inflammation directly by promoting inflammatory gene expression, or indirectly by triggering the inflammatory immune response and regulating cell death.^753^ Mechanistically, TNF-α binds as a homotrimer to two distinct homotrimeric receptors on the cell surface: TNFRI (p55 receptor) and TNFRII (p75 receptor),^754^ thus inducing downstream inflammatory mediators and growth factors, which further activates NF-κB and AP1.^754^ NF-κB signaling is a major mediator of protumor activity of inflammatory cytokines.^755^

TNF-α levels are abnormally elevated in various precancerous lesions, such as gastric lesions^754,756^ and inflammatory bowel disease, compared with normal tissues.^754,757^ In addition, TNF-α is overexpressed in the tumor and stroma of multiple malignancies, including breast cancer, ovarian cancer, CRC, prostate cancer, bladder cancer, esophageal cancer, renal cell cancer, melanoma, lymphoma, and leukemia.^752^ For example, ovarian cancer cells express 1000-fold more TNF-α mRNA than normal ovarian surface epithelial cells.^758^ The combination of upregulated TNF-α and C-reactive protein in the patient’s plasma is significantly related to shorter survival in HNSCC patients.^759^ In conclusion, TNF-α is a key regulator linking inflammation and tumorigenesis, and it may serve as a promising prognostic and therapeutic biomarker for tumor inflammation.^755^

##### NF-κB

NF-κB, first identified as a nuclear factor essential for immunoglobulin kappa light chain transcription in B cells in 1986,^760^ is a dimeric transcription factor. The mammalian NF-κB family consists of RELA (p65), NF-κB1 (p50; p105), NF-κB2 (p52; p100), c-REL, and RELB,^761,762^ all of which share a conserved amino-terminal region containing dimerization, nuclear localization, and DNA-binding domains. External stimuli, including infection factors, proteins, stress signals, and proinflammatory cytokines released by necrotic cells can activate NF-κB.^762^ The main activated form of NF-κB is a heterodimer of the p50 or p52 subunit associated with the p65 subunit.^762^ NF-κB proteins are present in the cytoplasm and are associated with inhibitory proteins of IκB. Activated IκB proteins are phosphorylated and ubiquitinated and then degraded by the proteasome, which induces NF-κB proteins to translocate to the nucleus.^762^ The nucleus NF-κB binds to cognate DNA-binding sites, promoting the transcription of various genes involved in cell cycle, proliferation, apoptosis resistance, and metastasis-promoting, ultimately enhancing cell growth, angiogenesis, stem cell formation, and cell metabolism.^762–764^

As a key regulator of inflammation,^765^ NF-κB is activated in various hematological and solid tumors and is closely associated with tumor development.^766^ A meta-analysis of 44 studies with a total of 4418 patients has revealed that NF-κB expression is connected to poor 3-year and 10-year OS in solid tumors.^767^ NF-κB level is significantly associated with large tumor size and high tumor grade in breast cancer patients.^768^ NF-κB also plays an important role in the TME. Activated NF-κB in cancer cells initiates and maintains the TME by upregulating chemokines that recruit immune response cells, inflammatory cells, and progenitors of cancer-associated fibroblasts.^769^ In addition, NF-κB regulates the EMT transition through the induction of EMT transcription factors.^770,771^ In conclusion, NF-κB is a prognostic biomarker of tumor inflammation in cancer.

##### NLRP3

NLRP3, belonging to the NLR protein family, is one of the most characterized inflammasomes.^749^ The NLR protein family has 22 members in humans.^772^ After the first inflammasome was discovered by Fabio Martinon in 2002,^773^ multiple PRRs have been identified and shown to be involved in inflammatory vesicle formation, such as NLRP1, NLRP2, NLRP3, and NLRC4.^774^

The inflammasome is a type of intracellular multiprotein hexamers or heptamers signaling complex that forms in cytoplasmic compartments, and the NLRP3 inflammasome has been intensively studied for its involvement in broad ranges of human diseases. Especially, dysregulation of the NLRP3 inflammasome is closely associated with the development of different cancers, including gastric cancer, CRC, HCC, head and neck cancer, lung cancer, breast cancer, prostate cancer, skin cancer, cervical cancer, and central nervous system tumors.^749^ A high NLRP3 level is correlated with the advanced tumor stage, distant metastasis, and the vascular invasion of cancers.^775^ It has been found that NLRP3 inflammasome promotes cancer cell differentiation by regulating cell cycle proteins and inducing the production of IL-1β which activates NF-κB by binding to its receptor, which ultimately leads to proliferation and invasion of gastric cancer.^749^ NLRP3 inflammasome activation in glioblastoma cells leads to IL-1β in aberrant expression.^776^ In addition, the NLRP3 inflammasome has been demonstrated to be elevated in HNSCC tissues, and its level is correlated with tumor prognosis.^777^ Activation of the NLRP3 inflammasome promotes the progression of prostate cancer,^778^ while reduced expression of NLRP3 inflammasome and IL-1β inhibits melanoma development.^779^ Furthermore, a high NLRP3 level is associated with a low 5-year and 10-year survival rate in CRC patients.^780^ Targeting NLRP3 inflammasome effectively inhibits HCC proliferation and metastasis.^781^ In conclusion, NLRP3 inflammasome activation leads to an inflammatory response that promotes cancer development and progression, and NLRP3 may serve as a prognostic and therapeutic biomarker for tumors.

#### Deregulating cellular metabolism

**Fig. 6:**
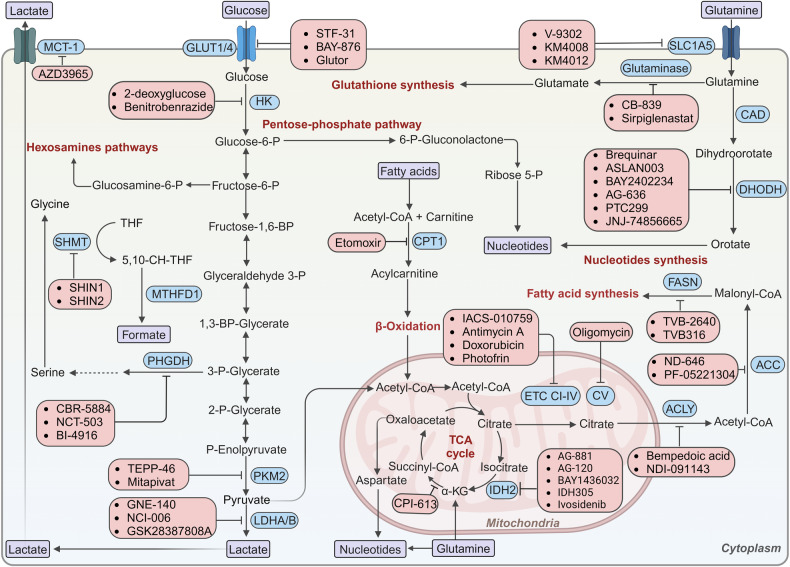
The potential inhibitors that target cancer metabolic process. Glucose is taken up into the cell by glucose transporters GLUT1/4 and phosphorylated by hexokinases HK1 and HK2. Glucose 6-phosphate (P) and its downstream intermediates can either be converted to pyruvate or fuel biosynthesis through different pathways, such as the pentose phosphate pathway which provides ribose 5-P for nucleotide synthesis. Fructose-6-P is involved in the hexosamine biosynthesis pathway. Glycerol 3-P production contributes to the serine and glycine biosynthesis pathways which are regulated by the key enzymes PHGDH and SHMT1/2. Moreover, serine biosynthesis plays an essential role in amino acid metabolism and nucleotide metabolism by regulating one-carbon metabolism which is mediated by the methylenetetrahydrofolate dehydrogenase MTHFD1. Pyruvate can be converted to lactate by LDH and exported through the monocarboxylate transporter MCT-1. Besides, pyruvate can enter the TCA cycle as acetyl-CoA through the mitochondrial pyruvate carrier and pyruvate dehydrogenase. Various pathways influence the production of the mitochondrial acetyl-CoA, including fatty acid β-oxidation, glucose metabolism, and other sources that can condense with oxaloacetate to form citrate, which can then be exported from the mitochondrion. Citrate via ACLY is a vital source of cytoplasmic acetyl-CoA which forms malonyl-CoA by acetyl-CoA carboxylase ACC1 and ACC2. Subsequently, malonyl-CoA is cyclically extended by the addition of carbons from acetyl-CoA by FASN to make saturated fatty acids. Fatty acid catabolism is initiated with the formation of fatty acyl-CoA which is then converted by CPT1 to an acylcarnitine. Pyrimidine synthesis, a multistep process regulated by key enzymes such as CAD and DHODH, can produce pyrimidine nucleotides from glutamine, carbonate, and aspartate. Meanwhile, glutamine is taken up by transporters SLC1A5. Glutamate produced from glutamine by glutaminase enzymes can be used in glutathione synthesis. In addition, the complex V (ATP synthase) and the electron transport chain consisting of four complexes including complex I/II/III/IV (CI–IV), are promising targets for drug development. Inhibitors (red), key enzymes or transporters (blue), and key metabolites (purple) are shown. ACC acetyl-CoA carboxylase, ACLY ATP-citrate lyase, BP bisphosphate, CAD carbamoyl-phosphate synthetase 2, aspartate transcarbamoylase, and dihydroorotase, CoA coenzyme A, CI–IV, complex I/II/III/IV, CV complex V, CPT1 carnitine palmitoyltransferase 1, DHODH dihydroorotate dehydrogenase, FASN fatty acid synthase, GLUT1/GLUT4 glucose transporter 1/4, HK hexokinase, IDH1 isocitrate dehydrogenase 1, LDHA/B lactate dehydrogenase A/B, MCT-1 monocarboxylate transporter 1, MTHFD1 methylenetetrahydrofolate dehydrogenase 1, P phosphate, PHGDH phosphoglycerate dehydrogenase, PKM2 pyruvate kinase M2, SHMT serine hydroxymethyl transferase, SLC1A5 solute carrier family 1 member 5, TCA tricarboxylic acid

Otto Warburg first discovered the tendency of tumors to convert glucose to lactate in the presence of oxygen in 1924, known as “aerobic glycolysis“,^782^ which subsequently came to be termed the “Warburg effect“.^783^ Tumor cells reprogram glucose metabolism even in the presence of oxygen by restricting energy metabolism mainly to glycolysis, thus reprogramming energy production. Extensive alterations in energy metabolism in cancer cells are considered to be important hallmarks of cancer^299^ (Fig. 6).

##### GLUT1

Tumor cells require the high uptake of glucose and glutamine to meet sustained proliferation.^784^ The polarity and hydrophilicity of glucose result in its inability to penetrate hydrophobic cell membranes. The transmembrane glucose transporter protein 1 (GLUT1, also known as SLC2A1) is the major glucose transporter protein, and GLUT1 expression is significantly upregulated in tumor cells.^784^ The crystal structure of human GLUT1 was first reported in 2014,^785^ and the expression of GLUT1 is regulated by various signaling pathways. The PI3K-AKT signaling pathway increases GLUT1 mRNA expression and drives GLUT1 protein transport from the inner membrane to the cell surface, thereby promoting glucose uptake.^786^ RAS upregulates GLUT1 mRNA expression and increases cellular glucose consumption.^787^ Tumor suppressor gene mutations, such as P53, block glycolysis by inhibiting GLUT1 expression.^788^ Additionally, the TME upregulates GLUT1 expression through HIF-1α.^787^

Overexpression of GLUT1 is an important biomarker for poor prognosis in multiple cancers, including breast cancer, ovarian cancer, prostate cancer, thyroid cancer, gastric cancer, HNSCC, glioblastomas, retinoblastomas, CRC, NSCLC, OSCC, esophageal cancer, urothelial papilloma, meningioma, brain cancer, diffuse large B-cell lymphoma, RCC, HCC, and cervical cancer.^789–792^ Studies have demonstrated that inhibitors targeting GLUT are an effective strategy for cancer treatment.^787^ In conclusion, GLUT1 is an essential target for tumor glucose metabolism, and it can be used as a diagnostic biomarker for tumors.

##### IDH1/2

Isocitrate dehydrogenase 1 (IDH1) is localized to peroxisomes and cytoplasmic lysosomes, whereas isocitrate dehydrogenase 2 (IDH2) is localized to mitochondria. Wild-type IDH1 and IDH2 metabolic enzymes catalyze the oxidative decarboxylation of isocitrate to generate α-ketoglutarate (α-KG). Cancer-associated IDH1 and IDH2 mutations occur almost exclusively at different arginine residues in the active site of the enzyme.^793^ IDH1 and IDH2 mutations occur in a wide variety of hematologic and solid tumors, including glioma, AML, intrahepatic cholangiocarcinoma, chondrosarcoma, thyroid cancer, and angioimmunoblastic T cell lymphoma.^793–795^ Mutant IDH1/2 catalyzes the conversion of α-KG to D-2-hydroxyglutarate (D2HG). D2HG is maintained at normal levels under physiological conditions, whereas mutant IDH leads to a large intracellular accumulation of D2HG in IDH mutant cancer. Elevated D2HG levels competitively inhibit α-KG-dependent lysine demethylases, leading to D2HG-induced dysregulation of histone and DNA methylation in cells, ultimately promoting tumor progression.^793^

IDH1/2 mutations have many advantages as easily detectable, reliable, and specific biomarkers. First, IDH1 and IDH2 mutations occur in highly restricted tumor types. Second, almost all tumor-derived mutant loci can be identified by simple PCR amplification and sequencing with a low volume of tumor samples. Third, IDH1 mutations can be identified by routine IHC.^796^ Fourth, techniques for the noninvasive detection of 2-hydroxyglutarate (2-HG) accumulation in glioma patients have been developed.^797^ Moreover, given that D2HG is upregulated in tumors with IDH mutations, elevated D2HG level in tumor tissues is used as a noninvasive detection biomarker for clinical IDH mutated tumors.^793^ In conclusion, IDH1/2 mutations are meaningful diagnostic biomarkers of tumor metabolism.

##### HK2

Hexokinases regulate the first step of glycolysis which produces and captures negatively charged glucose 6-phosphate ions within the cells. The hexokinases family has five isoforms in mammals including hexokinase 1–4 (HK1, HK2, HK3, HK4), and HKDC1.^798^ HK2 is the most active isozyme of the hexokinase family.^799^ In addition to being expressed in the muscle and heart,^798^ HK2 has been evaluated in various cancers, and is induced solely or synergistically by HIF-1 and MYC.^800^ An analysis of 21 studies with 2532 patients has revealed that HK2 overexpression is significantly associated with worse OS and PFS in solid tumors. For example, the negative effect of HK2 on OS is observed in HCC, gastric cancer, and CRC patients.^801^ HK2 expression is correlated with advanced-stage and high-grade ovarian cancer.^798^ HK2 downregulation inhibits tumor occurrence.^802^ Thus, HK2 is a meaningful prognostic tumor biomarker and a potential tumor treatment target.

#### Evading immune destruction

The immune system is responsible for monitoring and eliminating most early cancer cells, thereby inhibiting tumor formation. However, a significant increase in cancers due to low immune function is the evidence of defects in tumor immune surveillance. It has been found that cancer cells generate immune escape by disrupting the immune system, which ultimately promotes tumor progression, dissemination, and metastasis. Tumor cells suppress the action of cytotoxic lymphocytes by recruiting inflammatory cells with active immunosuppressive effects, such as regulatory T cells and myeloid-derived suppressor cells. Thus, immune evasion is another valuable hallmark of cancer.^299^

##### PD-1/PD-L1

PD-1 and PD-L1 participate in the evasion of the immune system by cancer cells.^803^ PD-1 (also called CD279), encoded by the *PDCD1* gene,^804^ was cloned and identified from an apoptotic immune cell line in 1992.^805^ PD-1 is a type I transmembrane protein receptor consisting of 288 amino acids, whose structure consists of an IgV-like extracellular domain, a transmembrane domain, and a cytoplasmic (intracellular) domain.^803^ As a negative regulator of the immune response,^806^ PD-1 is mainly expressed in memory T cells in peripheral tissues, and less in B cells, activated monocytes, dendritic cells, and NK cells.^803,804^ Two ligands of PD-1, PD-L1 (also known as B7-H1 or CD274) and PD-L2 (also known as B7-DC or CD273),^807^ are type I transmembrane protein receptors. PD-L1 is a 290 amino acid protein receptor encoded by the *Cd274* gene and includes two extracellular structural domains (IgV- and IgC-like domains), a transmembrane domain, and a cytoplasmic domain. Activated PD-1/PD-L1 signaling negatively regulates T cell-mediated immune responses in peripheral tissues, thereby limiting effector T cell responses and protecting tissues from damage.^803,804^

PD-1 signaling in the TME promotes tumor progression and survival by evading tumor immune surveillance. PD-1 is highly expressed in tumor-infiltrating lymphocytes in many types of cancers. PD-L1 is expressed on different types of tumor cells, including melanoma, ovarian cancer, lung cancer, and kidney cancer.^808^ The innate and adaptive immune resistance mechanisms contribute to the upregulated expression of PD-L1 in multifarious human cancers.^809^ A meta-analysis that analyzed 1251 patients from eight different microarray gene expression datasets has revealed that the expression levels of PD-1 and PD-L1 individually or jointly are potential prognostic factors for predicting the outcomes of patients with lung cancer.^810^ A study with 128 patients who are diagnosed with NSCLC, SCLC, melanoma, urothelial carcinoma, and other cancers, has proven that patients with high expression levels (>11.0 pg/μL) of soluble PD-L1 are more likely to exhibit progressive than those with low expression levels of PD-L1 (41.8 versus 20.7%). Moreover, a high expression level of soluble PD-L1 is also associated with a worse prognosis, the median PFS is 2.9 months versus 6.3 months, and the median OS is 7.4 months versus 13.3 months. Thus, high soluble PD-L1 is a predictive and prognostic biomarker for both decreased PFS and OS in advanced cancer patients who receive immune checkpoint blockade treatment.^811^ Moreover, a study of 293 HNSCC patients has concluded that strong PD-L1 expression is correlated with distant metastases, and dominates as the strongest prognostic factor of patient outcome.^812^ The PD-L1 expression level is a negative prognostic factor for patients with RCC^813^ and gastric cancer.^814^ Immune checkpoint inhibitors that block PD-1/PD-L1 interactions effectively prolong the survival of patients with various cancers and are promising cancer therapy.^803^ In conclusion, PD-/PD-L1 expression can be used as a predictive and prognostic biomarker for cancers.

##### CATL-4

Cytotoxic T lymphocyte antigen 4 (CTLA-4, also known as cluster of differentiation 152, CD152) is a receptor present on the surface of activated T cells, was discovered in 1987 by screening a cDNA library of mouse cytolytic T cell origin.^815^ CTLA-4 is normally expressed upon T cell activation,^816^ and activated CTLA-4 inhibits T cell proliferation and induces cell cycle arrest by cross-talk with PI3K and MAPK pathways that regulate cell proliferation.^816^ CTLA-4 is an inhibitory checkpoint commonly found in activated T cells and has been discovered to be the most reliable target for the treatment of cancer.^817^ CTLA-4 facilitates the tumor evasion of host immune surveillance, and participates in immune dysregulation in multifarious cancers, including lung cancer, cervical cancer, breast cancer, skin cancer, gastric cancer, CRC, B-cell CLL, and non-Hodgkin’s lymphoma.^818^ Moreover, targeting CTLA-4 significantly improves outcomes in multiple advanced cancers, including melanoma, lung cancer, breast cancer, head and neck cancer, bladder cancer, cervical cancer, liver cancer, gastric cancer, squamous cell skin cancer, classical Hodgkin’s lymphoma, and B-cell lymphoma.^816^

However, the correlation between CTLA-4 expression and patient prognosis in different cancers is controversial. Studies have found a significant correlation between the high expression of CTLA-4 and OS in single nucleotide polymorphisms subgroup cancers, including NPC, esophageal cancer, glioblastoma, and hematologic malignancy, in which CTLA-4 is a good prognostic biomarker.^818^ CTLA-4 overexpressed NSCLC is associated with a reduced death rate. Conversely, malignant pleural mesothelioma with high CTLA-4 exhibits poor prognosis.^818^ Higher CTLA-4 mRNA levels in breast cancer indicate higher clinical stage and axillary lymph node metastasis.^819^ A combined analysis of 844 ESCC patients has found that patients with both a low CTLA-4 and platelet lymphocyte ratio (PLR) level have longer OS.^820^ In conclusion, CTLA-4 is a prognostic biomarker in cancers and its positive or negative effects depend on specific cancer conditions.

#### Unlocking phenotypic plasticity

Cell development and organogenesis are accompanied by terminal differentiation that in most cases results in antiproliferative outcomes and suppresses tumor formation. It has been found that unlocking phenotypic plasticity to evade the state of terminal differentiation is a pivotal component of cancer development.^300^

Tumor cell differentiation is regulated by multiple factors. Liver enriched transcription factors are crucial regulators of hepatocyte differentiation and are essential for the maintenance of hepatocyte phenotype and function. Noncoding single-stranded RNA microRNAs are involved in the post-transcriptional regulation of gene expression, which is closely correlated with tumor dedifferentiation. The expression of miRNAs is negatively correlated with the degree of differentiation in HCC. Moreover, differentiation-related genes such as HMGCS2, BDH1, ALDH2, PIPOX, HAO1, AQP9, and PAH, have been identified to predict survival and poor prognosis in multifarious cancers.^821^

Differentiation and dedifferentiation are also essential for the developmental processes of many tumors. Melanocytes undergo dedifferentiation during tumorigenesis, and the malignant progression of pancreatic islet cell cancers to metastasis-prone carcinomas is associated with dedifferentiation. HDAC inhibitors induce the myeloid leukemia cell differentiation into mature myeloid morphology cells, thereby hindering the progression of leukemia.^300^ Furthermore, cellular plasticity in HCC is presented by the dynamic interconversion of cancer cell subpopulations in multiple developmental lineages and differentiation stages. Regardless, differentiation therapy unlocks phenotypic plasticity in HCC and induces terminal differentiation of CSCs, promoting their transformation into precursor cells that have lost self-renewal capacity, or converting them into non-CSCs that are sensitive to anticancer drugs.^821^ In conclusion, differentiation-related factors can be used as diagnostic and prognostic biomarkers for cancers.

#### Nonmutational epigenetic reprogramming

Since first described by Conrad Waddington in 1942,^822^ the epigenetic program of gene expression has become a hallmark of cancer that initiates and promotes tumorigenesis. The process of gene expression changes through pure epigenetic regulation is called “nonmutational epigenetic reprogramming”, which is different from genomic DNA instability and mutational mechanisms. Epigenetic alterations such as DNA methylation, histone modifications, chromatin remodeling, and noncoding RNA contribute to the signature ability during tumor progression.^300^

##### DNA methylation

DNA methylation is a chemical modification that plays a crucial role in chromatin-based transcriptional regulation, epigenetic gene expression, genomic stability, DNA repair, and replication. DNA methylation is mainly catalyzed by three DNA methyltransferases (DNMTs), DNMT1, DNMT3A, and DNMT3B.^823^ DNMTs are overexpressed in multiple cancers, including AML, CML, glioma, breast cancer, gastric cancer, CRC, HCC, pancreatic cancer, prostate cancer, and lung cancer.^824^ DNA methylation may lead to tumor suppressor gene silence, cell cycle dysregulation, DNA repair, and the misregulation of chromosomal stability genes, resulting in genomic instability in tumor cells.^825^ DNA methylation-based biomarkers have been hailed as an important event in cancer biomarker research.^826^ DNA methylation occurring mainly in centromeres, telomeres, inactive X-chromosomes, and repeat methylation is altered in 70% of mammalian promoter CpG islands, which are essential for gene transcriptional regulation and tumor malignant transformation.^827,828^ It has been found that 5–10% of CpG promoter islands are aberrantly methylated in various cancer genomes. DNMT1 is a CpG dinucleotides methyltransferase that recognizes hemimethylated DNA produced during DNA replication and methylates newly synthesized.^828^

The downregulation of tumor suppressor genes by hypermethylated CpG-rich regions of promoters is a typical example in tumor cells.^826^ Methylation of CpG dinucleotides (e.g., gene promoters) may serve as a clinically valuable biomarker. Moreover, CpG methylation is related to poor prognosis in patients with ccRCC.^829^ Methylation of GSTP1 has been discovered to be a promising diagnostic biomarker for HCC.^826^ The promoter methylations of NMDAR2B and PGP9.5 are linked to poor prognosis in patients with ESCC, and are meaningful clinical diagnostic and prognostic biomarkers for ESCC.^830^ The methylation status of single CpG dinucleotides affects the regulation of gene expression, and it can be utilized as a prognostic biomarker for CLL.^831^ O6-methylguanine-DNA methyltransferase (MGMT) methylation can also be used as a prognostic biomarker in glioblastoma patients.^832^

A study has searched the PubMed database for literature related to DNA methylation-based cancer biomarkers and retrieved a total of 14,743 research papers, which ultimately yields ~1800 tumor biomarkers through calculation and screening. However, only 13 DNA methylation-based biomarkers are currently commercially available and detectable, including GSTP1, APC, RASSF1, NDRG4, BMP3, SEPT9, SHOX2, TWIST1, OTX1, ONECUT2, MGMT, BCAT1, and IKZF1. Only nine of them (GSTP1, APC, RASSF1, NDRG4, BMP3, two SEPT9 biomarkers, SHOX2 and MGMT) have been included in the clinical guideline application.^826^ In addition, only two tests have been approved by the FDA: Cologuard (NDRG4 and BMP3), which analyzes stool DNA samples collected as part of a CRC screening protocol, and Epi proColon (SEPT9), which analyzes blood samples collected for the same purpose.^826^

As a promising biomarker for tumor diagnosis, prognosis, and prediction,^833^ DNA methylation has many advantages: frequent DNA methylation at the early stages of cancer, mature detection technology, good stability of DNA methylation in fixed samples, and presence in various body fluids and cell type specificity. Methylation at specific genomic sites can be a beneficial biomarker under the following conditions: clinically significant differences in methylation expression between the two groups, including diagnostic biomarkers in tumor versus nontumor tissues; prognostic biomarkers between tumor samples from patients with high-risk disease versus those with low-risk disease. In conclusion, DNA methylation in cancer is a clinically valuable biomarker for tumor management.^826^

##### Histone modification

Modification of histone proteins at amino-terminal tails such as acetylation, methylation, phosphorylation, and ubiquitination could alter the chromatin condensation, and DNA accessibility, subsequently interfering with gene expression. Histone modification is a dynamic process that is controlled by writers, such as histone acetyltransferases (HATs), histone methyltransferases (HMTs), readers, such as proteins containing bromodomains, and erasers, such as histone deacetylases (HDACs) and lysine demethylases. Histone modifications coregulate processes, such as DNA transcription, DNA replication, and DNA repair.^834^ Altered post-translational modifications of histones have been found in cancer cells, and changes in the overall level of histone modifications are found to predict clinical outcomes in various cancers.^835^

##### HATs

The N^ε^-acetylation of lysine residues is a major histone modification involved in the regulation of gene transcription, chromatin structure, and DNA repair. Acetylation neutralizes the positive charge of lysine, thereby weakening the electrostatic interaction between histones and negatively charged DNA. Thus, histone acetylation is associated with an open chromatin conformation. The HATs and HDACs family regulate the acetylation of histones.^828^ HATs are involved in a number of solid tumors and hematologic malignancies, and their expression levels are altered during tumor progression.^836^

##### HMTs

Histones are methylated on the side chains of arginine, lysine, and histidine residues and their methylation does not change the total charge of the molecule. The most characteristic sites of histone methylation are mono-, dimethyl- or trimethylation of lysine residues, including H3K4, H3K9, H3K27, H3K36, H3K79, and H4K20. Among them, H3K4, H3K36, and H3K79 are correlated with active genes in euchromatin, while H3K9, H3K27, and H4K2 are associated with heterochromatic regions of the genome.^828,837^ In addition, different methylation states on the same residue have different functions. For example, H3K4me2/3 usually spans the transcriptional start site of the active gene,^828^ while H3K4me1 is linked to active enhancers.^838^ Trimethylation of H3K9 is involved in the repression of gene expression.^828^

KMTs are specific enzymes that target certain lysine residues, including the members of the EZH2 family. EZH2 is the catalytic subunit of polycomb repressive complex 2 and is primarily responsible for the methylation of H3K27. Studies have shown that EZH2 overexpression is strongly associated with poor prognosis in prostate and breast cancers.^828^ Loss-of-function mutations in the EZH2 gene in myeloid malignancies and T cell acute lymphoblastic leukemia (T-ALL) also lead to poor prognosis.^839^

##### HDACs

HDACs are enzymes that reverse lysine acetylation and restore the positive charge on the side chain. HDACs can be classified into four major groups based on sequence homology: class I (HDAC 1–3 and HDAC8), class II (HDAC 4–7 and HDAC 9-10), class III HDAC (sirtuin 1–7), and class IV only (HDAC11).^828^ HDACs promote leukemia development by mediating abnormal gene silencing in malignant tumors. Inhibition of HDACs induces growth arrest, differentiation, and apoptosis in tumor cells.^840^ In addition, studies have demonstrated that HDACs are usually connected to poor tumor prognosis.^834^

##### HDMTs

LSD1 (KDM1A) is a class of demethylases that demethylate lysine through an oxidation reaction with flavin adenine dinucleotide, which is restricted to demethylating mono- and dimethyl lysine. Jumonji is a class of demethylases with a conserved JMJC structural domain that demethylates all three methyl lysine states through an oxidative mechanism and radical attack.^828^ The most studied LSD1 is increased in a variety of cancers, and it is related to the differentiation of neuroblastoma cells. In addition, HDMTs are involved in the development of breast cancer, PDAC, and other tumorigenic processes.^824^

#### Polymorphic microbiomes

The microbiota, an increasingly hot topic in recent years, has been demonstrated to influence the microenvironment, tumorigenesis, and metastasis of various malignancies.^841^ An increasing number of studies have uncovered that the microbiota is critical for the development of various cancers,^842^ including gastric cancer,^843^ ovarian cancer,^844^ CRC,^845^ pancreatic cancer,^846^ prostate cancer,^847^ HCC,^848^ lung cancer,^849^ breast cancer,^850^ and cholangiocarcinoma.^851^

There are three major categories of regulatory mechanisms by which microbiota promote carcinogenesis: altering the balance of host cell proliferation and death such as DNA damage and DNA repair; regulating the tumorigenic inflammatory environment within the tissue and immune system function; and affecting host metabolism.^852,853^ Therefore, small molecule drugs targeting microbiota have become a hot research topic in antitumor therapy.^854^ Known oncogenic gut microbiota include *Salmonella typhi*^855^ and *Helicobacter spp*^856^ in biliary tract cancer, *Helicobacter pylori*^857^ in gastric cancer, etc. *Helicobacter pylori* has been identified as a class I carcinogen by the World Health Organization, and it is associated with gastric cancer and mucosa-associated lymphoid tissue.^858^ CRC is a classic case of dysregulation of the gut microbiota that promotes cancer development.^859^ Certain microbiota species can ultimately exert a pro-carcinogenic effect by stimulating inflammatory states, including the induction of proinflammatory toxins, the increase of ROS production,^860^ the aberration of signaling pathways,^861^ and the blockage of antitumor immune function.^858^ It has been confirmed that *F. nucleatum* is crucial in the progression of CRC,^861^ which was detected in lymph nodes and distant metastasis samples from patients.^858^ *Peptostreptococcus anaerobius* is more enriched in stool samples from CRC patients, and its ability to induce phosphorylation of adherent spot kinase in CRC cells activates NF-κB signaling, ultimately promoting the cause of chronic inflammation and tumor progression. In addition, microbiota in other sites still play carcinogenic roles. Oral micro-common pathogenic bacteria including *Streptococcus anginosus, Veillonella, F. nucleatum*, and *P. gingivalis*, are involved in several digestive cancers. *P. gingivalis* is enriched in ESCC at higher levels than normal tissues, and it utilizes the miR-194/GRHL3/PTEN/AKT signaling pathway to promote ESCC proliferation and migration. The concentration of *F. nucleatum* nucleic acid is significantly higher in esophageal cancer tissues than in normal esophageal tissues. *P. gingivalis* and *F. nucleatum* are associated with a high risk of pancreatic cancer, and *P. gingivali*s can promote the proliferation of pancreatic cancer cells.^853^ Collectively, certain microbiota can be carcinogenic by stimulating chronic inflammatory response. In addition, microbiota can alter key intracellular signaling pathways and attack gastric mucosa by utilizing various virulence factors.^857^ In conclusion, the microbiota is essential for cancer development, and the microbiota can be used as a potential biomarker for tumors.

#### Senescent cells

Cellular senescence is a classic form of irreversible proliferative arrest characterized by the shutdown of the cell division cycle, changes in cell morphology and metabolism, and the activation of the senescence-associated secretory phenotype (SASP), which is capable of transmitting signaling molecules in a paracrine manner to neighboring living cancer cells as well as to other cells in the TME. SASP involves the release of a large number of bioactive protein chemokines, cytokines, and proteases. A variety of conditions, such as microenvironmental stress, and telomere erosion, induce cell senescence.^862–864^ The cellular senescence is a significant biomarker of tumor cells.

##### SASP

SASP is mediated through the proinflammatory transcription factor NF-kB or through transcriptional processes that depend on epigenetic changes.^863^ The NF-κB, p38, mTOR and C/EBPβ signaling pathways induce the formation of SASP^865^ which include proinflammatory cytokines (e.g., IL-1α, IL-1β, IL-6, and IL-8), chemokines (e.g., CCL2, CCL5, and CXCL1), growth factors (e.g., HGF, EGF, and TGFα), MMPs, and various oxylipins SASP factors. These factors are the main paracrine messengers between senescent cells and their surrounding cells (including stromal bystander cells, immune cells, precancerous cells, and cancer cells).^865^

The SASP exerts a double-edged sword effect in tumorigenesis, which might be beneficial or detrimental to tumorigenesis.^866^ On the one hand, SASP exhibits tumor suppressive effects by maintaining the senescence program, permanently blocking tumor transformation of normal cells, and recruiting immune cells to remove damaged or oncogene-expressing cells from the organism.^867^ The SASP factor IL-6 inhibits osteosarcoma formation by inducing and enhancing senescence.^868^ Precancerous lesions in RAS-driven pancreatic cancer are accompanied by extensive senescence and SASP.^869^ On the other hand, some SASP factors are tumorigenic. Studies have revealed that SASP factors promote tumorigenesis due to paracrine mitogenic or metastatic effects on other premalignant cells, as well as interactions with surrounding endothelial cells, stromal cells, and tissues.^867^ Senescent cells and SASP factors favor the promotion of cell transformation, metastasis, and tumor growth. Specific PTEN deficiency in mouse prostate cancer tissues leads to precancerous lesion development with extensive senescence and SASP.^867,870^ In general, SASP factors regulated by NF-kB have tumor-suppressive, immunosurveillance effects, while SASP factors regulated by signal transducer and activator of transcription 3 (STAT3) have tumor-promoting and immunosuppressive effects.^867^

##### Lamin B1

Lamins are intermediate filament proteins that line the inner surface of the nuclear envelop which contribute to the size, shape, and stability of the nucleus.^871^ Nuclear lamins are type V intermediate filaments ranging in size from 60 to 80 kDa. Lamins are divided into A type (lamin A, C) or B type (lamin B1, B2) according to isoelectric points. Lamin regulates nuclear and cytoskeletal organization, mechanical stability, chromatin organization, gene regulation, genomic stability, differentiation, and tissue-specific functions by binding to a variety of nuclear protein complexes.^871^ Lamin B1 is essential for the regulation of normal organogenesis and organism survival.^872^ Lamin B1 knockdown triggers the formation of H3K27me3-enriched mesas and DNA hypomethylation regions overlapping with lamin B1-associated domains in cancer and accelerates the replicative and oncogene-induced senescence. Reduced lamin B1 expression has been discovered in multiple senescent cells, and its overexpression delays senescence.^873,874^ Silencing lamin B1 expression slows cell proliferation and induces premature senescence in WI-38 cells.^873^ Oncogenic Ras-induced premature senescence also reduces lamin B1 expression through a pRb-dependent mechanism. In addition, senescence is induced by DNA damage, replication failure, or oncogene expression when lamin B1 is lost in human and mouse primary cells. Lamin B1 loss is not dependent on p38-MAPK, NF-κB, or ROS signaling pathways which are positive regulators of senescence phenotypes.^872^

On the other hand, Lamin B1 upregulation is widely observed in tumor tissues of most cancer types. A high level of lamin B1 expression predicts poor OS and DFS for cancer patients.^875^ Lamin B1 is overexpressed and facilitates cell proliferation and metastasis in HCC, and increased lamin B1 expression indicates a dismal prognosis and immunotherapy response in HCC.^876^ Besides, lamin B1 has been proposed as a prognostic senescence biomarker in ccRCC^877^ and lung adenocarcinoma.^878^ To summarize, lamin B1 loss is a senescence-associated biomarker.

### Liquid biopsy tumor biomarkers

Liquid biopsies have become a pivotal strategy for cancer diagnosis, real-time monitoring, and prognosis through minimally invasive detection of biofluids, such as blood, saliva, urine, pleural fluid, and ascites.^879^ Liquid biopsy tumor diagnostic biomarkers, including circulating tumor DNA, circulating tumor cells, and exosomes, are all effective monitoring tools for tumor diagnosis and treatment.

#### ctDNA

Cancer cells release naked DNA molecules into the circulation known as ctDNA which has become an essential biomarker for liquid biopsies to predict response to targeted therapies and immunotherapies to guide clinical anticancer treatment.^880^

ctDNA consists of small nucleic acid fragments that are not associated with cells or cellular fragments.^881^ Plasma ctDNA refers to tumor-derived DNA fragments and was detected in human plasma in 1948, and includes plasma cfDNA, circulating DNA derived from the death of hematopoietic cells.^882,883^ A study has revealed that ctDNA is detected in >75% of patients with advanced pancreatic cancer, ovarian cancer, CRC, bladder cancer, gastroesophageal cancer, breast cancer, melanoma, HCC, and head and neck cancer, and in 50% of patients with primary brain cancer, kidney cancer, prostate cancer, and thyroid cancer.^881^ As the plasma ctDNA content is less than 0.01%^884^ and its half-life is very short, plasma ctDNA levels allow for real-time dynamic assessment of tumor evolution through serial sampling, and represent intrapatient and interpatient variability to guide clinical drug use. In addition, plasma ctDNA captures tumor heterogeneity and effectively reflects DNA shed from multiple metastatic sites.^884^ The NCCN Guidelines for Breast Cancer (version 4.2020) recommend the use of ctDNA analysis for the evaluation of PIK3CA mutations in breast cancer.^884,885^

Moreover, ctDNA analysis is used for clinical real-time monitoring of treatment response in a variety of tumors, including breast cancer,^886^ NSCLC,^887^ prostate cancer, gastroesophageal cancer,^884^ HCC,^50^ and CRC.^888^ Plasma ctDNA analysis has been applied to monitor clinical cancer therapy resistance. Metastatic CRC is the first disease that utilizes liquid biopsy to evaluate treatment resistance. ctDNA analysis identifies CRC resistance to HER2-targeted therapy. The NCCN Guidelines for Gastric Cancer (version 2.2020) and the Esophageal and Esophagogastric Junction Cancer Guidelines (version 2.2020) recommend the use of plasma ctDNA analysis to detect drug sensitivity in patient treatment.^884^ Osimertinib resistance in NSCLC patients with EGFR mutation can be detected by plasma ctDNA analysis.^889^ Prostate cancer plasma ctDNA is used to detect BRCA reversion which mediates PARPi treatment resistance.^890^ In summary, ctDNA is widely used in patients with advanced solid tumors for the detection of MRD, the monitor of early recurrence, the prediction of treatment response, and drug resistance monitoring.^58,888^

#### CTCs

In 1869, Thomas Ashworth, an Australian physician, first identified CTCs, a type of cells shed into the bloodstream from primary or metastatic tumor sites.^891^ CTCs are cancer cells isolated from the primary tumor site and transported via the circulation to distant organs.^892^ CTC characteristics are defined as a nucleated circulating cell larger than 4 μm expressing the epithelial cell protein EpCAM and cytokeratin 8, 18, and 19, while not expressing the leukocyte-specific antigen CD45.^893^ This is the main basis and foundation of CTC testing.^891^

CTCs are clinically significant as biomarkers for the clinical management of patients with metastatic cancer and the implementation of precision medicine.^894^ More than 400 clinical trial studies have been conducted using CTCs as biomarkers. The key research contents and objectives are assessing prognostic information, the risk of recurrence and metastasis, stratifying and monitoring treatment in real-time, and identifying therapeutic targets and resistance mechanisms of cancer patients.^895^ CTC assays and molecular characterization have been applied to stratify patients with breast cancer, CRC, prostate cancer, lung cancer, pancreatic cancer, glioblastoma multiforme, and melanoma, and to monitor disease progression.^892^ Studies have discovered that CTC count is a prognostic indicator for multiple myeloma.^896^ The application of CTCs in the clinical assessment of gastric cancer has shown that CTCs are correlated with metastasis, poor prognosis, recurrence, and treatment response in gastric cancer patients.^891^ The comprehensive analyses of CTCs in metastatic breast cancer confirmed heterogeneous mechanisms of patient resistance to targeted therapies.^897^ CTCs can monitor therapeutic response and be utilized as a screening tool for brain micro-metastasis detection in breast cancer.^898^ In addition, CTCs have important clinical utility in the selection of therapeutic-specific biomarkers for the treatment of patients with prostate cancer. The response of prostate cancer to anticancer drugs is strongly correlated with the expression of AR-V7, a treatment-specific biomarker, in the CTC nuclei.^899^ The CTCs in the NSCLC pulmonary vein are independent predictors of NSCL recurrence after surgery.^900^ In addition, CTC analysis captures the tumor heterogeneity noninvasively and real-timely in cancer patients, which effectively explains the existence of heterogeneous drug resistance mechanisms in patients with refractory tumors^901^ and promotes the development of precise targeting strategies.^901^ In conclusion, CTC analysis is a clinically relevant noninvasive tool to monitor the progression and prognosis of cancers, and to deeply explore the biology of metastatic cancers, and has the potential to facilitate personalized precision treatment of cancers. Of course, the difficulties undoubtedly need to be solved in the future, including that the efficiency of CTC targeting approaches varies by cancer types, and the large patient cohorts are needed to flesh out the arguments in CTC application.^894^

#### Exosomes

Exosomes, first discovered in 1983 by Pan and Johnstone,^902^ are extracellular lipid bilayer vesicles of endosomal origin with an average size range of approximately 100 nm in diameter.^903^ The process of exosome biogenesis is well defined, starting with double invagination of the plasma membrane and the formation of intracellular multivesicular bodies containing intraluminal vesicles, followed by exocytosis of multivesicular bodies fused to the plasma membrane, and the intraluminal vesicles are finally secreted as exosomes with a diameter of ~40–150 nm.^904^ Exosomes released from the cell surface can fuse with the plasma membrane of recipient cells, thereby transporting their contents into the cytoplasm. In addition, proteins on the surface of exosomes can bind to the surface receptors of recipient cells to promote intracellular signaling.^905^ The exosomes are highly heterogeneous given their size, content, functional impact on recipient cells, and cellular complexity of origin.^903^ Among them, the content of exosomes is a key factor in the execution of functions. Recent advances have revealed that the contents of exosomes include proteins, DNA, mRNA, microRNA, long noncoding RNA, circular RNA, and other components.^906^

Exosomes have been demonstrated to be involved in cancer development, angiogenesis, metastasis, and therapeutic resistance, and can be used as diagnostic and prognostic biomarkers for tumor patients.^906^ For example, exosomes are useful in liquid biopsy to diagnose various cancers, including lung cancer,^907^ pancreatic cancer,^908^ gastric cancer,^909^ prostate cancer, breast cancer, ovarian cancer, glioblastoma, and melanoma.^905^ The exosomal DNA in serum exosomes has been proven to be of significant value in detecting cancer-related mutations, such as KRAS and TP53.^910,911^ The specific miRNAs that are differentially expressed in exosomes between cancer cells and normal cells have important diagnostic or prognostic value in the early detection of cancers.^912^ Studies have revealed that elevated serum-derived exosomal miR-21 is associated with multiple cancers, including pancreatic cancer, ovarian cancer, and lung cancer.^913^ The tumor suppressor miRNAs, such as miR-146a and miR-34a, are found in exosomes, and their low levels are correlated with poor prognosis of liver cancer, breast cancer, CRC, pancreatic cancer, and hematologic malignancies.^903,912^ The upregulation of other exosomal oncogenic microRNAs, such as miR-155, miR-17-92, and miR-1246, have also been shown to be connected to the progression of multiple cancers.^903,912,914^

Moreover, as the exosomal cargo exchange between cancer cells and stromal cells in the TME, exosomes from multiple cancer cells can effectively regulate TME angiogenesis and extracellular matrix remodeling at metastatic sites, thereby enhancing tumor growth and metastasis.^903,904^ Breast cancer-derived exosomes have been demonstrated to impair vascular integrity and enhance vascular permeability, thereby promoting breast cancer metastasis.^915^ Exosomes from glioblastoma cells promote tumor cell proliferation and induce angiogenesis.^916^ Ovarian cancer cell exosomes are involved in promoting their peritoneal dissemination.^917^ Moreover, cancer cell-derived exosomes also facilitate metastasis in melanoma,^918^ pancreatic cancer,^919^ gastric cancer, etc.^920,921^ Exosomes from different cellular sources, such as immune cells, cancer cells, epithelial cells, and mesenchymal cells, can influence the activity of recipient cells of both the innate and adaptive immune system. Exosomes stimulate or inhibit the function of CCD4^+^ and CD8^+^ T cells.^903^

In addition, exosomes secreted by cancer cells can benefit cell survival by interacting directly with drugs and reducing their antitumor efficacy, or by regulating cancer cell gene expression through TEM cell-derived exosomes. CAF-derived exosomes stimulate chemotherapy resistance in CRC^922^ and breast cancer,^923^ and promote resistance of ovarian cancer cells to paclitaxel.^924^ Moreover, macrophage-derived exosomes induce resistance to gemcitabine in pancreatic cancer cells.^925^ Thus, exosomes secreted by cancer cells can induce resistance to chemotherapeutic drugs.^903^

Exosomes have numerous advantages as liquid biopsy diagnostic biomarkers for cancer. First, exosomes can be secreted by all types of cells and are present in various biological fluids, such as blood, urine, semen, saliva, amniotic fluid, cerebrospinal fluid, ascites, tears, and breast milk.^905^ Therefore, it is easy and convenient to collect samples. Second, exosomes can reveal specific proteins from parental cells and target cells, which isolates the origin-specific exosomes and predict organ-specific metastasis.^117^ Third, the concentration of exosomes is higher than that of other liquid biopsy biomarkers, such as CTCs, thereby reducing the amount of sample collection. Fourth, exosomes are highly stable compared with other liquid biopsy biomarkers, such as ctDNA, which is rapidly degraded in the blood.^926^ In conclusion, exosomes are valuable biomarkers for the early diagnosis, prognosis prediction, and the therapeutic efficacy assessment of cancers. The possibility of combining protein, lipid, RNA, and miRNA exosome cargos in cancer may enhance the diagnostic and prognostic potential of exosomes, which is being considered.

## Tumor biomarker-based cancer therapy

Optimizing the precise medical care of patients according to genetic and molecular characteristics can maximize the benefits of precision medicine. Therefore, discovering and developing tumor biomarkers and related cancer therapies contribute greatly to effective precision medicine. Compared with traditional chemotherapy drugs, targeted cancer therapy including small-molecule targeted drugs, monoclonal antibodies (mAbs), antibody-drug conjugates, and the proportion of biological drugs specifically targeting proteins or genes in cancer cells, results in high potency and low toxicity. During the past 30 years, remarkable progress has been achieved in this field. Targeted therapy has been the mainstream strategy for cancer therapy, and targeted drugs approved by the FDA, European Medicines Agency, Ministry of Health Labour and Welfare and National Medical Products Administration for cancer treatment have increased accordingly.^927,928^ Here, we summarize the pivotal tumor biomarker-based cancer therapies in preclinical and clinical studies in recent years and hope to provide comprehensive insights into cancer therapy.

### Targeting tumor cell proliferative signaling

There is an increasing number of inhibitors related to tumor growth signaling pathways, including inhibitors of RAS, PI3K-AKT-mTOR, and RAF-MEK-ERK signaling pathways. In particular, multiple protein kinases have engaged in cell proliferation, and targeted drugs have rapidly developed and been applied for clinical use since the approval of the first small-molecule TKI, imatinib, by the FDA in 2001.^929^ Although their targets and mechanisms of action are different, all of them can effectively inhibit tumor cell proliferation.

#### RAS inhibitors

Since the discovery of RAS in the rat sarcoma virus, targeting RAS has attracted attention because of its vital role in tumorigenesis and progression. However, the development of RAS targeting therapy is extremely challenging.^930^ The RAS protein surface is very smooth, and the lack of drug-binding pockets makes targeting RAS problematic.^931^ The existence of high intracellular GTP concentrations, the activation of compensation pathway, and drug-resistant mutations in RAS-RAF-ERK pathway genes after RAS inhibitors administration, lead to poor inhibitory effects of RAS inhibitors.^319,932^ In recent years, due to the consecutive failure in the discovery of RAS inhibitors, RAS was once considered an undruggable target. Significantly, Shokat and colleagues opened a new chapter in RAS-targeted therapy by discovering a binding pocket containing the mutant cysteine residue in KRAS^G12C^ in 2013, which prompted the fast development of the first small-molecule KRAS^G12C^ inhibitor sotorasib.^319^ Subsequently, due to the importance of RAS in tumorigenesis and progression, alternative strategies such as targeting RAS mutations, and upstream and downstream effectors have been attempted. For example, drug exploitation in targeting RAS mutated malignancies can be accomplished using various strategies (Table 1), including interfering with RAS maturation and transport, promoting its localization to the plasma membrane, and inhibiting its downstream signaling.^933^ To date, inhibitors against KRAS^G12C^ have been approved for clinical use.^308^ Meanwhile, RAS inhibitor combination options are increasingly developed, i.e., combination with inhibitors that inhibit the RAS signaling pathway, maintain the GDP-binding status of KRAS proteins, and modulate the immune system.^319^

**Table 1:** The FDA-approved and clinically developed RAS inhibitors in cancer therapies

Target	Drug	Highest Phase	Indications	Company/Identifier	Status
Farnesyltr-ansferase	Tipifarnib	Approved	Head and neck squamous cell carcinoma harboring HRAS mutations who have progressed following platinum-containing chemotherapy	Kura Oncology	/
	Lonafarnib (SCH66336)	Approved	Hutchinson-Gilford progeria syndrome and progeroid laminopathies	Merck & Co	/
		III	Carcinoma, non-small cell lung, metastases, neoplasm	NCT00050336	Terminated
		III	Myelodysplastic syndromes, leukemia, myelomonocytic, chronic, myelodysplasia, myelomonocytic	NCT00109538	Terminated
	Salirasib	II	Non-small cell lung cancer	NCT00531401	Completed
SOS	BI-1701963	II	Advanced solid tumors, KRAS^G12C^ mutation	NCT04185883	Recruiting
	MRTX0902	I/II	Solid tumor, advanced solid tumor, non-small cell lung cancer, colorectal cancer	NCT05578092	Recruiting
SHP2	RMC-4630	II	Non-small cell lung cancer	NCT05054725	Active, not recruiting
		I/II	Solid tumor	NCT03989115	Completed
		I/II	Advanced solid tumors, KRAS^G12C^ mutation	NCT04185883	Recruiting
		I/II	Metastatic neoplasm	NCT04418661	Active, not recruiting
	TNO155	I/II	KRAS^G12C^ mutant solid tumors, non-small cell lung, carcinoma, colorectal, lung cancer, pulmonary cancer	NCT04699188	Recruiting
		I/II	Advanced cancer, metastatic cancer, malignant neoplastic disease	NCT04330664	Active, not recruiting
		I/II	Advanced solid tumors, KRAS^G12C^ mutation	NCT04185883	Recruiting
	RLY-1971	I	Advanced solid tumors, metastatic solid tumors	NCT05487235	Recruiting
		I	Solid tumor, unspecified, adult	NCT04252339	Completed
		I	Colorectal cancer, non-small cell lung cancer	NCT05954871	Recruiting
KRAS^G12C^	Adagrasib (MRTX849)	Approved	Solid tumors harboring KRAS^G12C^ oncogenic driver mutation, including non-small cell lung cancer and colorectal cancer	Mirati Therapeutics	/
	Sotorasib (AMG510)	Approved	KRAS^G12C^-mutated locally advanced or metastatic non-small cell lung cancer	Amgen	/
	JNJ-74699157 (ARS-3248)	I	Neoplasms, advanced solid tumors, non-small cell lung cancer, colorectal cancer	NCT04006301	Completed
	Divarasib (GDC-6036)	III	Non-small cell lung cancer	NCT03178552	Recruiting
	D-1553	II	Non-small cell lung cancer	NCT05383898	Recruiting
		II	Non-small cell lung cancer	NCT05492045	Recruiting
		II	Solid tumor, non-small cell lung cancer, colorectal cancer	NCT04585035	Recruiting
		II	Solid tumor	NCT05379946	Not yet recruiting
	JDQ443	III	Non-small cell lung cancer	NCT05132075	Recruiting
	RMC-6291	I	Non-small cell lung cancer, colorectal cancer, pancreatic ductal adenocarcinoma, advanced solid tumor	NCT05462717	Recruiting
Non-KRAS^G12C^	MRTX1133	I/II	Solid tumor, advanced solid tumor, non-small cell lung cancer, colorectal cancer, pancreatic adenocarcinoma	NCT05737706	Recruiting
	RMC-6236	I	Non-small cell lung cancer, colorectal cancer, pancreatic ductal adenocarcinoma, advanced solid tumors	NCT05379985	Recruiting
siRNA strategies	KRAS^G12D^ iExosomes	I	KRAS^G12D^ metastatic pancreatic adenocarcinoma, pancreatic ductal adenocarcinoma	NCT03608631	Recruiting
Antisense oligonucleotide	AZD4785	I	Non-small cell lung cancer, advanced solid tumors	NCT03101839	Completed
RAS analog	Rigosertib	III	Metastatic pancreatic adenocarcinoma	NCT01360853	Completed
		III	Myelodysplastic syndromes, chronic myelomonocytic leukemia	NCT01928537	Completed
		III	Myelodysplastic syndromes, chronic myelomonocytic leukemia	NCT01241500	Completed
Cancer vaccines	mRNA-5671	I	Non-small cell lung cancer pancreatic cancer, colorectal cancer	NCT03948763	Completed
Antibody	NS1	II	Hematologic malignancy, acute leukemia, acute myeloid leukemia, acute lymphoblastic	NCT05735717	Recruiting

Source: All the information is derived from ClinicalTrials.gov (https://www.clinicaltrials.gov) and the United States Food and Drug Administration.gov (https://www.fda.gov/)

#### KRAS^G12C^ mutation inhibitors

Extensive studies on the structure and biofunction of KRAS mutations have shed light on the development of drugs targeting KRAS mutations. In particular, the development of KRAS mutation inhibitors has been encouraged by the approval of the first small-molecule KRAS^G12C^ inhibitor sotorasib (AMG510) for the treatment of KRAS^G12C^ mutant NSCLC patients by the FAD in 2021.^934,935^ Sotorasib is an FDA-approved KRAS^G12C^-specific covalent inhibitor that irreversibly binds to the GDP-binding inactive conformation of KRAS^G12C^, thus blocking its activity.^931^ Clinical trials have shown that sotorasib exerts anticancer activity in advanced solid tumor patients with KRAS^G12C^ mutations, including NSCLC,^936^ CRC, pancreatic cancer, endometrial carcinoma, appendiceal cancer, and melanoma.^937^ The first randomized phase III trial of targeting the KRAS^G12C^ inhibitor has revealed that sotorasib significantly increases PFS in NSCLC patients with KRAS^G12C^ mutation.^938^ Meanwhile, phase II clinical trials of sotorasib used for the treatment of previously treated locally advanced or metastatic NSCLC subjects with KRAS^G12C^ mutation or comorbidities are ongoing (NCT05631249 and NCT05311709). However, the objective response rate to sotorasib monotherapy is still far from satisfactory. A phase II clinical trial has shown that the objective response rate to sotorasib monotherapy in patients with advanced KRAS^G12C^-mutated CRC is only 9.7%, indicating that its combination treatment strategy for KRAS^G12C^-mutated CRC needs to be further evaluated.^939^ Following the successful development of sotorasib, adagrasib (MRTX849), an irreversible KRAS^G12C^ inhibitor received its first approval by the FDA for the treatment of advanced or metastatic NSCLC patients with KRAS^G12C^ mutation in December 2022. Adagrasib has a favorable pharmacokinetic profile, such as a long half-life (~24 h), broad tissue distribution, and dose-dependent pharmacokinetics.^940^ Notably, adagrasib penetrates the cerebrospinal fluid and causes the regression of lesions in patients with KRAS^G12C^ mutant NSCLC brain metastases.^941^ Clinical trials have demonstrated that adagrasib monotherapy is well tolerated with a disease control rate of 87% in 46 patients, and adagrasib in combination with cetuximab has shown clinical activity in patients with KRAS^G12C^-mutated CRC. Moreover, adagrasib in combination with cetuximab is currently in a phase III clinical trial (NCT04793958) in patients with KRAS^G12C^ mutant CRC.^942^ The approval of sotorasib and adagrasib has opened the door to the possibility of developing more effective RAS^G12C^ inhibitors. ARS-853 is a highly cell-active, KRAS^G12C^ mutant-specific covalent inhibitor that targets the GDP-bound inactive state of KRAS and prevents its activation, resulting in the abrogation of KRAS mutation-induced signaling.^930^ ARS-853 is the first direct targeting KRAS inhibitor by covalently reacting with the RAS-GDP complex to trap it in its inactive state.^930^ Other KRAS^G12C^ mutation inhibitors such as JNJ-74699157 (ARS-3248), divarasib (GDC-6036), garsorasib (D-1553), JDQ443, and RMC-6291 are undergoing different phases of clinical trials in cancer patients (Table 1).Although extensive development of KRAS^G12C^ inhibitors is ongoing, they only work in a small fraction of patients with KRAS^G12C^ mutation, and the median PFS is fairly short, less than 1 year. Thus, an in-depth study of the resistance mechanism of KRAS^G12C^ mutation inhibitors is urgently needed. To date, KRAS inhibitor resistance mechanisms are distinguished into intrinsic resistance and acquired resistance.^931,943^ Intrinsic resistance is mainly influenced by the KRAS status in the cells. The cancer cells carrying KRAS mutations can be divided into two categories, KRAS-dependent and KRAS-independent,^944^ the latter of which may be less sensitive to KRAS inhibitors. Similarly, KRAS knockdown PDAC cells are able to maintain cell proliferation through PI3K alternative bypass pathway.^931,945^ The mechanisms of acquired resistance can be broadly classified into three categories: (1) KRAS alterations: mutations in the KRAS Y96, R68, and H95 residues. KRAS^Y96D^ mutation can affect the binding pocket of inhibitors to KRAS protein switch-II.^931,946^ KRAS G12D/V/R, G13D, and Q61H mutations similarly cause resistance to KRAS G12C. Certain tumor cells produce new KRAS^G12C^ mutation after inhibitor treatment which maintains KRAS active and results in drug resistance.^931^ (2) Altered vertical signaling cascades: changes in upstream signaling pathways affecting KRAS-GTP binding affinity, downstream signaling pathways, such as MEK-ERK^947^ and EGFR,^947^ and other upregulation can all induce drug resistance.^931^ (3) Changes in the TME such as immune escape pathways produce resistance to KRAS^G12C^ inhibition^931,948^ Therefore, the combination of KRAS^G12C^ inhibitors with other drugs is considered an efficacious way to improve their antitumor effect. The results of clinical trials have proven that KRAS^G12C^ inhibitor combination strategies are well tolerated with no serious adverse effects in most patients. Of note, many clinical trials of the KRAS^G12C^ inhibitor combination are currently underway for the treatment of NSCLC and other solid tumors.^931^

In summary, KRAS^G12C^ inhibitors have promising therapeutic prospects for both monotherapy and combination use in cancer treatment. Given that KRAS inhibitor resistance is still a serious challenge in clinical practice, it remains to be elucidated in deeper detail to achieve precise therapies for cancer patients.

#### Non-KRAS^G12C^ mutation inhibitors

The promising advances in KRAS^G12C^ inhibitors have brought light to target other KRAS mutations. The KRAS^G12C^ mutation only represents a small part of the mutations, and other mutations include G12D, G12V, G12S, and G12R.^931^ MRTX1133 is a selective noncovalent KRAS^G12D^ inhibitor^949^ with a high affinity for KRAS^G12D^ and effectively inhibits the tumor growth of PDAC xenograft mouse models in vivo.^950^ RMC-9805 is a selective, orally bioavailable covalent KRAS^G12D^ inhibitor that effectively blocks the growth of KRAS^G12D^ mutant cancer cells in vitro and in vivo.^931^ In addition, RMC-6236 is a pan-RAS inhibitor that inhibits all RAS mutant subtypes,^931^ and is currently in phase I clinical trial (NCT5379985) in advanced solid tumors with KRAS mutations. However, due to the lack of active residues and intrinsic hydrolytic activity in these mutated proteins, the development of highly potent inhibitors against the above KRAS mutations remains a big challenge.^931^

The fast development of the above inhibitors targeting KRAS mutations may take advantage of the “addiction” of tumors to mutant RAS and create more effective regimens for more patients such as pancreatic cancer patients. The in-depth study of KRAS-specific mechanisms by which they enhance or hinder cancer proliferation will provide new insights for subsequent inhibitor development.

#### Other RAS targeting strategies

Existing KRAS^G12C^ inhibitors have narrow therapeutic windows and are only effective in a small proportion of cancer patients with KRAS mutation. Many studies are currently dedicated to discovering alternative strategies for targeting RAS. Pan-RAS inhibitors broaden the therapeutic windows by directly targeting multiple RAS-mutated cancers.^951^ The pan-RAS inhibitor RAS-IN-3144 can block the growth of KRAS^G13D^ mutant MDA-MB-231 cell-derived mouse xenograft tumors in vivo.^952^ A synthetic sos protein mimic has been found to suppress RAS activation as a pan-RAS inhibitor.^953^ Moreover, rigosertib is a RAS analog that inhibits RAS-mediated pancreatic cancer growth by interacting with the RAS-binding domain of RAF kinase, resulting in the inability of RAF.^954^ Additionally, oligonucleotides can inhibit protein synthesis by boosting mRNA degradation or interfering with translation.^306^ AZD4785 is an antisense oligonucleotide that targets KRAS mRNA with high affinity. It inhibits downstream signaling pathways by depleting cellular KRAS mRNA and proteins, thus exerting antitumor effects.^955^ A phase I clinical trial of AZD4785 (NCT03101839) in patients with KRAS mutated solid tumors has been completed, but no results have been posted. In addition, KRAS dimerization is essential for downstream signaling when KRAS is localized at the plasma membrane.^951^ NS1, a synthetic binding protein antibody, interferes with RAS dimerization and blocks CRAF-BRAF heterodimerization and activation.^951,956^ Recently, proteolysis-targeting chimeras (PROTACs) technology can directly degrade targeted proteins,^308^ which have been used to specifically degrade KRAS in cancer cells.^957^ In addition, CRISPR/Cas9 screening is also applied in the identification of RAS synthetic lethal gene.^958^ In addition, fibroblasts-derived exosomes loaded with G12D siRNA (iExosomes) can effectively restrain PDAC tumor growth.^959,960^ Mesenchymal stromal cells-derived exosomes with KRAS are currently in a phase I clinical trial (NCT03608631) evaluating efficacy in patients with pancreatic cancer. A phase I clinical trial (NCT03948763) of the KRAS mRNA vaccine V941 (mRNA-5671) in KRAS-mutated NSCLC, pancreatic cancer, and CRC has been completed, but no results have been posted.

##### Targeting upstream of RAS

***Targeting RAS plasma membrane localization****.* Only plasma membrane localization of RAS proteins can stimulate downstream effectors and signaling pathways. If post-translational modification and membrane localization are blocked, RAS proteins will be inactive, suggesting that the inhibition of RAS membrane localization is an effective therapeutic strategy.^951^

Farnesyltransferase plays a vital role in RAS localization. The farnesyltransferase inhibitors (FTIs) can prevent RAS localization in the plasma membrane by suppressing farnesyltransferase, leading to the blockade of downstream signaling.^931^ Tipifarnib, an orally bioavailable, nonpeptidomimetic quinolinone FTI,^961^ was granted a fast track designation by the FDA in December 2019 for the treatment of patients with HNSCC harboring HRAS mutations who have progressed following platinum-containing chemotherapy. The objective response rate of tipifarnib in a phase II clinical study of HRAS-mutated recurrent and/or metastatic HNSCC was 55%, with common adverse effects of anemia and lymphocyte reduction.^962^ Subsequently, tipifarnib was given fast track status again by the FDA for the treatment of adult patients with different subtypes of peripheral T-cell lymphoma. Moreover, tipifarnib has been demonstrated to be effective in advanced refractory uroepithelial carcinoma with HRAS mutation,^963^ in recurrent and metastatic salivary gland carcinoma with HRAS mutation,^964^ and in HRAS-driven dedifferentiated thyroid cancers.^965^ Lonafarnib, originally discovered by Merck & Co as an investigational oncology drug^966^ and known as the world’s first drug approved by the FDA in November 2020 for the treatment of progeria and progeroid laminopathies, is also an orally active FTI that blockades RAS localization in the plasma membrane. Salirasib is a farnesylcysteine mimetic that blocks the function of all RAS isoforms (H-RAS, K-RAS, and N-RAS) by interfering with RAS binding to the plasma membrane.^967^ Numerous preclinical studies have shown the ability of salirasib to inhibit the proliferation of various human cancer cells,^967^ including breast cancer,^968^ glioblastoma,^969,970^ CRC,^971^ melanoma,^972^ ovarian cancer,^973^ HCC,^974^ and pancreatic cancer.^975^ However, a phase II clinical study revealed poor therapeutic activity of salirasib in KRAS-mutant lung cancer,^976^ and its development in subsequent clinical trials has been discontinued.^931^

Phosphodiesterase-δ phosphodiesterase-δ (PDEδ) is a prenyl-binding protein involved in regulating the membrane localization and signaling of farnesylated RAS. PDEδ binds and solubilizes farnesylated RAS proteins to enhance their diffusion in the cytoplasm and transfers RAS from the Golgi apparatus and endomembranes to the plasma membrane, thereby facilitating RAS enrichment at the plasma membrane and signaling transduction.^977–979^ Deltarasin inhibits KRAS-dependent proliferation of human PDAC cells by competitively binding the farnesyl-binding pocket of PDEδ and reducing RAS enrichment at the plasma membrane.^978^ Deltazinone has a similar mode of action to deltarasin, but possesses a higher selectivity and lower cytotoxicity.^979^ However, the fast release of KRAS-PDEδ inhibitors from PDEδ hindered drug-binding affinity, which resulted in poor antiproliferative activity of PDEδ inhibitors.^980^ Novel strategies, such as PDEδ degraders are underway.^980^

***SOS1 inhibitors****.* Son of sevenless homolog 1 (SOS1), a guanine nucleotide exchange factor (GEF), catalytically promotes the activation of RAS which in turn consecutively enhances the GEF function of SOS1.^306^ Targeting SOS1 to disturb its interaction with KRAS in tumors is referred to as an effective way to inhibit a wide panel of KRAS-driven cancers.^930,981,982^ Multiple SOS1 inhibitors have been developed, mainly including quinazoline-based SOS1 inhibitors such as BAY-293 and BI-3406. BAY-293, first reported by Bayer and identified by combining high throughput screening and fragment screening in 2019, is a selective SOS1 inhibitor that suppresses the KRAS-SOS1 interaction at an IC_50_ value of 21 nM.^983,984^ BI-3406, an orally selective SOS1 inhibitor with quinazoline structure developed by Boehringer Ingelheim, can bind to the SOS1 catalytic domain, reduce GTP-RAS formation, and impair MEK inhibitor-induced feedback activation, eventually inhibiting KRAS-driven cancer cell proliferation.^985^ The most advanced SOS1 inhibitor is BI-1701963, an analog of BI-3406, which has demonstrated safety in clinical phase I trials and is under phase I clinical trial as monotherapy and in combination with trametinib in patients with KRAS mutated advanced or metastatic solid tumors (NCT04111458).^931^ MRTX0902 is a selective, orally bioavailable SOS1 inhibitor with antitumor effects in combination with MRTX849 in KRAS^G12C^ mutant NSCLC,^931^ and a phase I/II study of MRTX0902 in solid tumors with mutations in the KRAS MAPK pathway is ongoing (NCT05578092). In addition to the above representative small-molecule agonists, ZZ151, a potent, cooperative, and selective SOS1 PROTAC, has shown superior anticancer activities in KRAS^G12D^ and KRAS^G12V^ mutant xenografts in mice, which is worth further optimization.^986^ However, there is no marketable SOS1 compound, and only two cases are in clinical studies, with a lack of publicly disclosed clinical data. Exploiting highly selective and low-toxicity SOS1 inhibitors is a major research focus in the future.

***SHP2 inhibitors****.* SHP2, a nonreceptor protein tyrosine phosphatase encoded by the PTPN11 gene, promotes GEF-mediated RAS-GTP interactions and activates the downstream RAS-RAF-ERK pathway.^987,988^ RMC-4630 is a selective orally bioavailable allosteric SHP2 inhibitor,^988^ which is undergoing a clinical phase I trial (NCT03634982) of monotherapy in participants with advanced relapsed or refractory solid tumors. A clinical phase I trial (NCT04916236) combining RMC-4630 and the ERK inhibitor LY3214996 for the treatment of KRAS-mutated cancer is ongoing. In addition, RMC-4630 and sotorasib are being used in combination in a clinical phase II trial (NCT05054725) to explore antitumor effects in patients with KRAS^G12C^-mutated NSCLC. Similarly, TNO155, a selectively orally bioavailable SHP2 inhibitor,^989^ is being used alone or in combination with EGF816 (nazartinib) in phase I clinical trial (NCT03114319) for advanced solid tumor treatment. Other SHP2 inhibitors are also being tested in clinical trials. A clinical phase I trial of RLY-1971 in patients with advanced or metastatic solid tumors (NCT04252339) has been completed, but results have not yet been disclosed. SHP099 is a potent and selective small molecule SHP2 inhibitor that inhibits SHP2 activity by binding to the N-terminal and C-terminal ends of SH2 and the protein tyrosine phosphatase structural domain. SHP099 effectively suppressed tumor cell proliferation in vivo and in vitro by blocking RAS-ERK signaling.^990^ However, SHP2 inhibitor-based development has come a long way in the past few years. Earlier developed SHP2 inhibitors targeting the catalytic site failed in clinical practice due to poor selectivity and low bioavailability. Therefore, the development of SHP2 metathesis inhibitors has become an important research direction. Moreover, SHP2 inhibitors have a wide scope in drug combinations. In addition, due to the extensive expression of SHP2, a strategy that induces toxic effects and increases the safety of SHP2 inhibitors on normal cells is a primary problem to be solved.

##### Targeting RAS downstream effectors

The most classical downstream effector pathways of RAS are the RAF-MEK-ERK cascade pathway and the PI3K-AKT-mTOR pathway. A large number of inhibitors targeting the RAF-MEK-ERK and PI3K-AKT-mTOR pathways have been developed and are under clinical evaluation.^958^

***PI3K-AKT-mTOR pathway inhibitors***. Dysregulation of the PI3K-AKT-mTOR pathway is critical to the oncogenesis and progression of many human tumors, and their inhibitor development is of great significance^991^ (Table 2). However, the PI3K-AKT-mTOR pathway inhibitors have various resistance mechanisms. For example, the treatment of PI3K pathway inhibitors can result in the feedback activation of upstream signaling pathways, thus limiting their efficacy^992^

**Table 2:** The FDA-approved and clinically developed PI3K-AKT-mTOR inhibitors

Type	Target	Drug	Highest Phase	Indications	Company/Identifier	Status
PI3K	Pan-PI3K	Copanlisib (BAY80-6946)	Approved	Relapsed follicular lymphoma	Bayer	/
		Buparlisib (BKM120)	III	Head and neck cancer	NCT04338399	Recruiting
			III	Metastatic breast cancer	NCT01633060	Terminated
			III	Breast cancer	NCT01610284	Completed
			III	Breast cancer	NCT01572727	Completed
		SF1126 (prodrug of LY294002)	I	Neuroblastoma	NCT02337309	Terminated
	p110α	Alpelisib (BYL719)	Approved	ER + /HER2-advanced metastatic breast cancer	Novartis Pharmaceuticals	/
		Inavolisib	III	Breast cancer	NCT05646862	Recruiting
			III	Breast cancer	NCT04191499	Recruiting
			III	Metastatic breast cancer	NCT05894239	Recruiting
	p110β	AZD8186	I	Advanced castrate-resistant prostate cancer, squamous non-small cell lung cancer, triple-negative breast cancer	NCT01884285	Completed
		GSK2636771	II	Melanoma and other malignant neoplasms of skin, metastatic melanoma	NCT03131908	Active, not recruiting
			II	Advanced lymphoma, advanced malignant solid neoplasm, hematopoietic and lymphoid cell neoplasm, refractory lymphoma, refractory malignant solid neoplasm, refractory multiple myeloma	NCT04439188	Active, not recruiting
			II	Advanced lymphoma, advanced malignant solid neoplasm, hematopoietic and lymphoid cell neoplasm, refractory lymphoma, refractory malignant solid neoplasm, refractory multiple myeloma	NCT04439149	Active, not recruiting
			I/II	Advanced gastric adenocarcinoma	NCT02615730	Completed
	p110γ	IPI-549	II	Head and neck squamous cell carcinoma, head and neck cancer, head and neck carcinoma, head and neck cancer stage IV, head and neck cancer stage III, HPV-Related carcinoma, HPV-Related malignancy, HPV-Related squamous cell carcinoma	NCT03795610	Recruiting
			II	Bladder cancer, urothelial carcinoma, solid tumor, advanced cancer	NCT03980041	Completed
			II	Breast cancer, renal cell carcinoma	NCT03961698	Active, not recruiting
	p110δ	Idelalisib	Approved	Relapsed chronic lymphocytic leukemia	Gilead Sciences	/
		Duvelisib	Approved	Relapsed or refractory chronic lymphocytic leukemia, small lymphocytic lymphoma	Verastem	/
		Umbralisib	Approved	Relapsed or refractory marginal zone lymphoma	TG Therapeutics	/
Dual PI3K/mTOR		Omipalisib (GSK2126458)	I	Solid tumors	NCT00972686	Completed
AKT		Capivasertib (AZD5363)	Approved	HR + , HER2- locally advanced or metastatic breast cancer with one or more PIK3CA/AKT1/PTEN-alterations	AstraZeneca	/
		Ipatasertib (GDC0068)	III	Breast cancer	NCT04060862	Active, not recruiting
		GSK2141795	II	Cervical cancer	NCT01958112	Terminated
			II	Melanoma	NCT01941927	Completed
			II	Recurrent adult acute myeloid leukemia, untreated adult acute myeloid leukemia	NCT01907815	Terminated
			II	Estrogen receptor negative, HER2/Neu negative, invasive breast carcinoma, progesterone receptor negative, recurrent breast carcinoma, stage IV breast cancer, triple-negative breast carcinoma	NCT01964924	Completed
		MK-2206	II	Colorectal neoplasms	NCT01333475	Completed
			II	Ovarian sarcoma, recurrent fallopian tube carcinoma, recurrent ovarian carcinoma, recurrent primary peritoneal carcinoma	NCT01283035	Completed
			II	Recurrent nasopharyngeal carcinoma	NCT01370070	Completed
mTOR	mTORC1	Everolimus	Approved	Advanced renal cell carcinoma following one prior antiangiogenic therapy	Novartis	/
			Approved	HR+ and HER2- breast cancer	Novartis	/
		Temsirolimus	Approved	Advanced renal cell carcinoma	Wyeth Pharmaceuticals	/
	mTORC1/ mTORC2	AZD2014	II	Meningioma	NCT03071874	Active, not recruiting
			II	Neurofibromatosis 2 meningioma	NCT02831257	Completed
			II	Diffuse large B-cell lymphoma	NCT02752204	Completed
			I/II	Endometrial carcinoma, metastatic carcinoma, HR+ tumor	NCT02730923	Active, not recruiting
			II	Metastatic breast cancer	NCT02299999	Active, not recruiting
			II	Non-small cell lung cancer metastatic	NCT02117167	Active, not recruiting
			II	Hepatocellular carcinoma	NCT03591965	Terminated
		INK128	II	Metastatic castration-resistant prostate cancer	NCT02091531	Completed
			II	merkel cell carcinoma	NCT02514824	Completed
			II	anaplastic thyroid cancer, thyroid cancer	NCT02244463	Active, not recruiting
		OSI-027	I	Any solid tumor or lymphoma	NCT00698243	Completed

Source: All the information is derived from ClinicalTrials.gov (https://www.clinicaltrials.gov) and *the* United States Food and Drug Administration.gov (https://www.fda.gov/)

***PI3K inhibitors***. Numerous PI3K inhibitors have been developed and can be divided into three major classes: pan-PI3K inhibitors, isoform-specific PI3K inhibitors, and dual PI3K/mTOR inhibitors (Table 2). Most PI3K inhibitors are currently in clinical trials, and some of them (copanlisib, alpeilisib, idelalisib, duvelisib, and umbralisib) are approved by the FDA. However, the approvals/accelerated applications of some PI3K inhibitors, such as idelalisi, duvelisab, and umbrailisib, have been withdrawn due to frequent and severe adverse effects.^993^

Pan-PI3K inhibitors simultaneously inhibit the four catalytic subunits of class I PI3K p110α (PIK3CA), p110β (PIK3CB), p110γ (PIK3CG), and p110δ (PIK3CD). LY294002 and wortmannin are the first generation of pan-PI3K inhibitors, which build the foundation for the exploitation of novel high-efficiency and low-toxicity PI3K inhibitors. Unfortunately, the clinical applications of LY294002 and wortmannin are seriously limited by obvious adverse effects. LY294002 has poor aqueous solubility and adverse effects including severe respiratory depression and lethargy. Wortmannin has similar poor pharmacological properties, including a short half-life, chemical instability, and side effects, such as liver dysfunction and lymphocytopenia.^993^ Copanlisib (BAY80-6946) is an intravenous Pan-PI3K inhibitor developed by Bayer that inhibits α, β, γ, and δ isoforms with varying degrees of affinity,^994^ and received accelerated FDA approval in 2017 for the treatment of recurrent follicular lymphoma.^993^ Buparlisib (BKM120) is an oral ATP-competitive pan-PI3K inhibitor, being used for the treatment of stage II ESCC.^995^ The good brain penetration of buparlisib makes it a promising drug for the treatment of intracranial tumors.^996^ Buparlisib is currently being evaluated in a clinical phase III trial (NCT04338399) in combination with paclitaxel for the treatment of HNSCC. However, the lack of selectivity of pan-inhibitors results in nonselective inhibition of the PI3K pathway, leading to serious adverse side effects.

Researchers developed selective ATP-competitive inhibitors for each isoform of PI3K to limit the emergence of toxic effects. Isoform-specific PI3K inhibitors selectively inhibit p110α, p110β, p110δ, or p110γ subunits with greatly improved off-target effects. Alpelisib (BYL719), an oral inhibitor targeting PI3Kα developed by Novartis, was approved by the FDA for ER+/HER2-advanced metastatic breast cancer treatment in 2019.^993^ Inavolisib (GDC-0077) is also an oral inhibitor targeting PI3Kα with multiple ongoing clinical trials,^997^ including a phase III clinical trial (NCT05646862) for the treatment of HR+, HER2−, PIK3CA mutated breast cancer, and a phase II clinical trial (NCT05306041) for HR+, HER2+, PIK3CA mutated breast cancer. There are a few PI3Kβ-specific inhibitors, including GSK2636771,^998,999^ and AZD8186, developed by AstraZeneca.^999^ In addition, PI3Kδ inhibitors have mostly been approved by the FDA for clinical treatment. Idelalisib, developed by Gilead Sciences, was approved by the FDA in 2014 for the treatment of patients with relapsed CLL. Duvelisib, developed by Verastem, was approved by the FDA in 2018 for the treatment of relapsed or refractory CLL and small lymphocytic lymphoma, and subsequently received accelerated approval for adult patients with relapsed or refractory FL.^1000^ Umbralisib was developed by TG Therapeutics and received accelerated approval by the FDA in 2021 for the treatment of adult patients with relapsed or refractory marginal zone lymphoma.^1001^ Finally, no PI3Kγ inhibitors are currently available for clinical treatment. IPI-549, a potent and highly selective PI3Kγ inhibitor developed by Infinity Pharmaceuticals,^1000^ is being used in clinical phase II trials as a single agent for the treatment of HNSCC (NCT03795610) and in combination therapy for TNBC (NCT03961698). Furthermore, dual-targeted PI3K and mTOR inhibitors also exert potential roles in cancer therapy. Omipalisib (GSK2126458), a dual-targeted PI3K and mTOR inhibitor developed by GlaxoSmithKline,^993^ has completed a phase I clinical trial in solid tumors (NCT00972686), but no results have been disclosed yet.

In summary, although the side effects of PI3K inhibitors that can lead to serious and fatal immune-related adverse reactions remain an urgent issue,^1002^ PI3K inhibitors have great potential in clinical treatment. Exploring PI3K inhibitors in combination with other targeted therapies may be an effective strategy to reduce toxicity and improve clinical activity.^346^

***AKT inhibitors***. AKT belongs to the serine/threonine kinases family,^343^ which contains three isoforms with highly similar structures: AKT1, AKT2, and AKT3, making the development of their isoform-specific inhibitors challenging.^346^ Meaningfully, tumors with AKT1 mutations and AKT2 and AKT3 amplifications are highly sensitive to AKT inhibitors, while many PIK3CA mutant cancer cells were considerably less dependent on AKT,^1003^ which guides the rational use of AKT inhibitors. Currently, various selective ATP-competitive pan-AKT inhibitors (Table 2), including capivasertib by AstraZeneca Pharmaceuticals,^1004^ ipatasertib (GDC0068),^1005^ and GSK2141795,^1006^ have been developed which can inhibit all three AKT isoforms.^1003^ Significantly, on November 16, 2023, the FDA approved capivasertib (truqap) with fulvestrant for adult patients with HR+, HER2− locally advanced or metastatic breast cancer with one or more PIK3CA/AKT1/PTEN-alterations. In addition, AKT allosteric inhibitors such as the potent allosteric pan-AKT inhibitor MK-2206 possess better AKT specificity.^1006^ However, concerns such as the poor selectivity and toxicity of AKT inhibitors remain a burning challenge. Presently, PROTAC and AKT drug combination strategies may change the landscape of AKT drug development.

***mTOR inhibitors***. The mTOR pathway participates in multiple tumor cell processes, and hyperactivated mTOR signaling is observed in different types of cancers. Suppression of mTOR was approved by the FDA and the EMA as an effective strategy capable of inhibiting the PI3K-AKT-mTOR signaling.^343^ Currently, drug development against mTOR is a hot track, and mTOR inhibitors can be categorized into three generations: antibiotic allosteric inhibitors (first generation), ATP-competitive inhibitors (second generation), and novel mTOR inhibitors (third generation) (Table 2).^1007^ Rapamycin and its analogs (termed rapalogs) are members of the first generation of allosteric inhibitors of mTOR. Significantly, the rapamycin analogs everolimus (RAD001) has been approved for the treatment of RCC,^1008^ HR+ and HER2− breast cancer.^1009^ On May 30, 2007, temsirolimus (CCI-779) was approved for the treatment of advanced RCC.^1010^ Temsirolimus and metformin in combination with other drugs are currently in clinical phase I trials to evaluate their safety and dosage appropriateness in tumor patients. Researches on rapalogs have been relatively mature, but are further challenged by defects of large molecular weight, complex structure, difficulty in synthesis, and limited modification sites.^1007^ Therefore, the second-generation of small molecule-based mTOR inhibitors with simplified structures is a promising direction in the field of mTOR inhibitor development.^1007^ The second-generation mTOR inhibitors are structurally quite different from the first-generation mTOR inhibitors. They selectively target the active kinase site of mTOR and thus act as ATP-competitive inhibitors. A large number of ATP-competitive mTOR inhibitors are currently in clinical trials, including MLN01289 (INK128) in a phase II clinical trial (NCT02244463) for the treatment of anaplastic thyroid cancer. AZD2014 is in a phase I clinical trial (NCT02398747) for the treatment of advanced solid malignancies and a phase II clinical trial (NCT03071874) for the treatment of meningioma. OSI-027, an oral selective mTOR inhibitor, is being tested in a phase I clinical trial for advanced solid tumors or lymphoma (NCT02398747). A phase I clinical trial for AZD8055 in adults with recurrent gliomas (NCT01316809) has been completed, but no results have been disclosed. The third-generation mTOR inhibitor RapaLinks are important research breakthroughs that link rapamycin to ATP-competitive mTOR inhibitors via linkers to improve drug efficacy.^1007^ In addition, some natural products such as resveratrol can directly or indirectly regulate mTOR and mTOR signaling pathways.^1007^ Moreover, differences in mTOR inhibitor administration formula, doses, and oral bioavailability are found to result in differences in drug exposure and efficacy.^1011^ Improving the administration formula and combination with targeted drugs may be effective strategies to improve mTOR inhibitors.

##### RAF-MEK-ERK inhibitors

The RAF-MEK-ERK pathway regulates intracellular growth signaling^1012^ and is activated in more than 30% of human cancers.^1013^

BRAF is a commonly mutated protein kinase in human cancers, particularly frequent BRAF V600 point mutation in melanoma, and it has been thought to be an ideal target for cancer therapy.^354^ The first-generation selective RAF inhibitors, such as vemurafenib and dabrafenib, have been well-demonstrated to possess therapeutic effects in patients with BRAF^V600E^ or BRAF^V600K^ mutations although their efficacy was abrogated by quick-rising drug resistance.^1014^ To improve the efficiency and overcome drug resistance of first-generation RAF inhibitors, second-generation novel RAF inhibitors such as pan-RAF inhibitors and RAF dimer breakers have been developed.^1015^ Until now, DAY101 is the fastest progressing pan-RAF inhibitor in a phase II study to evaluate its safety and efficacy in patients with recurrent or progressive low-grade glioma or advanced solid tumors harboring a known BRAF alteration (NCT NCT04775485, NCT05760586). KIN-2787 is another pan-RAF inhibitor that is under a phase I clinical trial in adults with BRAF/NRAS-mutated advanced or metastatic solid tumors (NCT04913285). The phase I clinical trials of the pan-RAF inhibitor LY3009120 and LXH254 in advanced or metastatic cancers have been terminated because of their unfavorable efficiency.^1016–1018^ However, a phase I clinical trial of LXH254 in combination with LTT462 or trametinib or ribociclib for the treatment of KRAS or BRAF mutant NSCLC or NRAS mutant melanoma (NCT02974725) is ongoing.^951^ In addition, PLX8349 is an orally available, second-generation BRAF inhibitor with IC_50_ values of 3.8 nM, 14 nM, and 23 nM for BRAF^V600E^, BRAF, and CRAF, respectively. Importantly, PLX8349 effectively suppresses mutant BRAF cells without activating the MAPK pathway, thereby overcoming the resistance of first-generation RAF inhibitors.^1019^

Moreover, the inhibitors of MEK and ERK which are typical effectors of the RAS-RAF signaling pathway have been developed. Most MEK inhibitors are allosteric inhibitors rather than ATP-competitive inhibitors that block ERK phosphorylation via MEK.^951^ The oral ERK inhibitor MK-8353 is well tolerated and has good antitumor activity in BRAF^V600^ mutant melanoma patients.^1020^ The ATP-competitive ERK inhibitor SCH772984 effectively inhibited ERK1 and ERK2 activity and significantly suppressed the proliferation of BRAF, NRAS, or KRAS mutated tumor cells.^1021^ Clinical phase I trials have indicated that the ATP-competitive ERK1/2 kinase inhibitor ulixertinib (BVD-523) has antitumor effects in patients with advanced solid tumors.^1013^ In addition to monotherapy by the above inhibitors, the combination of MEK inhibitors or ERK inhibitors with RAF inhibitors effectively blocks the feedback activation pathway induced by RAF inhibitors or ERK inhibitors in BRAF-mutated or KRAS-mutated cancer cells.^1022^ In addition, the combination of the RAF-MEK-ERK pathway and PI3K-AKT-mTOR pathway for tumor-targeted therapy is a promising therapeutic strategy,^951^ which potently curbs the growth of various cancers such as KRAS-mutant lung cancer,^1023^ NRAS-mutated melanoma,^1024^ pancreatic cancer,^1025^ and many other cancers.^1026^

Taken together, inhibitors of the RAF-MEK-ERK pathway exhibit promising anticancer efficiency, although drug resistance impedes their further use. The in-depth investigation of the interaction of these inhibitors and growth regulatory mechanisms in specific tumor cell contexts and environments will improve the therapeutic effects and benefit future drug development. The combination of drug strategies based on RAS-targeted therapies and new technological approaches such as RNAi and CRISPR technologies will shed light on tumor therapy.^992^

### Targeting evading growth suppressors

Cancer cells can maintain tumor progression by circumventing processes that negatively regulate cell proliferation which are supported by numerous tumor suppressor genes, such as RB and TP53. Therefore, targeting evading growth suppressors is a promising antitumor strategy.^299^ Indeed, CDK4/6 and MDM2 inhibitors are currently being used in clinical treatment or under development.^1027,1028^

#### CDK4/6 inhibitors

Relaxation of cell cycle mechanisms is associated with the dysregulation of CDKs, which ultimately promotes abnormal tumor proliferation and disease progression.^1027^ Over the past few decades, three generations of CDK inhibitors (CDKIs) have been developed. First- and second-generation CDKIs receive few clinical attention in the treatment of cancer patients due to their limited specificity and high toxicity. The development of third-generation CDKIs has made significant progress in clinical practice. Preclinical and clinical results suggest that these selective CDK4/6 inhibitors significantly reduce the progression of multiple malignancies.^1029^ Early in the cell cycle, mitotic signaling increases cyclin D expression, which binds to and activates CDK4/6. CDK4/6 phosphorylates the Rb protein which is further phosphorylated by CDK2, followed by releasing the E2F transcription factor which allows the cell to enter S phase.^1030^ Selective CDK4/6 inhibitors bind to ATP pockets, prevent CDK4/6 from binding to cyclin D, and prevent Rb phosphorylation, leading to G1 phase arrest and tumor cell death.^1031^ Currently, the typical CDK4/6 inhibitors palbociclib, ribociclib, and abemaciclib have been approved by the FDA for the treatment of different cancers alone or in combination with established therapies (Table 3).^1032–1034^

**Table 3:** The FDA-approved and clinically developed CDK4/6 inhibitors

Target	Drug	Highest Phase	Indications	Company/identifier	Status
CDK4/6	Palbociclib (PD0332991)	Approved	HR+, HER2− advanced breast cancer in combination with hormonal therapy	Pfizer	/
	Ribociclib (LEE-011)	Approved	HR+, HER2− advanced breast cancer in combination with hormonal therapy	Novartis	/
	Abemaciclib (LY2835219)	Approved	HR+, HER2− advanced breast cancer in combination with hormonal therapy, monotherapy for advanced HR+, HER2− breast cancer, adjuvant therapy for high-risk, early-stage HR+, HER2− breast cancer in combination with hormonal therapy	Eli Lilly	/
	Trilaciclib	Approved	Approved to reduce chemotherapy-induced bone marrow suppression in patients with extensive-stage small cell lung cancer	G1 Therapeutics	/
	TQB3616	III	HR+, HER2− in advanced breast cancer	NCT05375461	Recruiting
		III	Breast cancer	NCT05780567	Recruiting
		III	HR+, HER2− breast neoplasms	NCT05365178	Not yet recruiting
	SPH4336	III	Locally advanced or metastatic breast cancer	NCT05860465	Not yet recruiting
		III	Locally advanced or metastatic breast cancer	NCT05744687	Recruiting
	Dalpiciclib (SHR6390)	III	Advanced breast cancer	NCT05861830	NCT05861830
		III	Female breast cancer	NCT04842617	NCT04842617
		III	Advanced breast cancer	NCT03966898	NCT03966898
	Flavopiridol (L86-8275)	I/II	Lymphoma	NCT00445341	NCT00445341
		I/II	B-cell chronic lymphocytic leukemia, recurrent small lymphocytic lymphoma, refractory chronic lymphocytic leukemia, waldenström macroglobulinemia	NCT00058240	NCT00058240
		II	Leukemia, lymphocytic, chronic	NCT00464633	Recruiting
		II	B-cell chronic lymphocytic leukemia, refractory chronic lymphocytic leukemia, stage I–IV chronic lymphocytic leukemia,	NCT00003620	Completed
		II	Refractory multiple myeloma, stage I–III multiple myeloma	NCT00047203	Completed
		II	Adenocarcinoma of the pancreas, recurrent pancreatic cancer, stage IV pancreatic cancer	NCT00331682	Completed
		II	Sarcoma	NCT00005974	Completed
		II	Prostate cancer	NCT00003256	Completed
		II	Adenocarcinoma of the gastroesophageal junction, diffuse adenocarcinoma of the stomach, intestinal adenocarcinoma of the stomach, mixed adenocarcinoma of the stomach, recurrent gastric cancer, stage IIIA gastric cancer, stage IIIB gastric cancer, stage IIIC gastric cancer, stage IV gastric cancer	NCT00991952	Completed
		II	Lymphoma	NCT00003039	Completed
		II	Kidney cancer	NCT00016939	Completed
		II	Melanoma (skin)	NCT00005971	Completed
		II	Endometrial cancer	NCT00023894	Completed
		II	Lymphoma	NCT00005074	Completed
		II	Liver cancer	NCT00087282	Completed
		I/II	Recurrent adult acute lymphoblastic leukemia, recurrent adult acute myeloid leukemia, secondary acute myeloid leukemia, untreated adult acute lymphoblastic leukemia, untreated adult acute myeloid leukemia	NCT00016016	Completed
		II	B-cell chronic lymphocytic leukemia, prolymphocytic leukemia, refractory chronic lymphocytic leukemia	NCT00098371	Terminated
		II	Head and neck cancer	NCT00020189	Completed
		II	Esophageal cancer	NCT00006245	Completed
		II	Breast cancer	NCT00020332	Completed

Source: All the information is derived from ClinicalTrials.gov (https://www.clinicaltrials.gov) and the United States Food and Drug Administration.gov (https://www.fda.gov/)

Palbociclib, an orally reversible small molecule inhibitor developed by Pfizer, is the first selective CDK4/6 inhibitor. The chemical structure of palbociclib was identified in 2004,^1030^ and subsequently followed by 11 years of development. In February 2015, Palbociclib in combination with letrozole was accepted for accelerated approval by the FDA for the treatment of ER+, HER2− advanced breast cancer as initial endocrine-based therapy in postmenopausal women. Thereafter, in February 2016, the FDA approved palbociclib in combination with fulvestrant for the treatment of HR+, HER2− advanced or metastatic breast cancer in women with disease progression following endocrine therapy, followed by the approval of palbociclib in combination with an aromatase inhibitor for women with HR+, HER2− advanced or metastatic breast cancer and men in April 2019.^1035^ In preclinical studies, palbociclib exhibits significant antiproliferative effects on Rb-positive cells and leads to the selective arrest of the G1 phase in a series of tumor cells.^1036^ In a phase II clinical trial of palbociclib, the efficacy and safety of letrozole with or without palbociclib in the treatment of ER+, HER2− postmenopausal breast cancer patients were compared. The PFS of palbociclib plus letrozole versus letrozole alone was 20.2 months versus 10.2 months. The adverse effects especially hematologic aspects have been proven to be higher in the combination group compared with the letrozole monotherapy group.^1037^

Ribociclib is the second selective inhibitor of CDK4/6 developed by Novartis and approved by the FDA in 2017 for the treatment of patients with HR+, HER2− advanced or metastatic breast cancer.^1030,1038^ The IC_50_ values of ribociclib for CDK4 and CDK6 are much lower than those of other kinases, at 10 and 39 nM, respectively.^1029^ Ribociclib is used as a single agent or in combination with other drugs in preclinical studies. A phase III clinical trial of ribociclib has evaluated the efficacy and safety of ribociclib plus letrozole in patients with HR+, HER2− relapsed, or metastatic breast cancer. Results have shown PFS after 18 months in the ribociclib group versus placebo group 63.0 versus 42.2%. Moreover, adverse effects in the ribociclib group included nausea, infection (mainly upper respiratory tract infections and urinary tract infections), fatigue, diarrhea, neutropenia, leukopenia, hypertension, elevated alanine aminotransferases, lymphopenia, and QTc interval prolongation.^1034^ In the clinical trial MONALEESA-7, the efficacy and safety of ribociclib in combination with tamoxifen or nonsteroidal aromatase inhibitors have been observed in the treatment of advanced breast cancer with HR+ and HER2-. Moreover, ribociclib plus nonsteroidal aromatase inhibitors have been found to benefit more in advanced breast cancer without new adverse effects.^1039^

Abemaciclib is an oral CDK4/6 inhibitor developed by Eli Lilly with IC_50_ values of 2 and 10 nM for CDK4 and CDK6, respectively, and is approved by the FDA for the treatment of patients with HR+, HER2− advanced or metastatic breast cancer.^1040^ Abemaciclib is more active and less neutropenic than palbociclib and ribociclib.^1041,1042^ In addition, numerous studies have shown that abemaciclib can cross the blood-brain barrier into the central system, suggesting the possibility of treating primary or metastatic brain cancer.^1043^ In preclinical studies of abemaciclib, both monotherapy and in combination with endocrine therapy with abemaciclib have been found to have significant inhibitory effects on cancers.^1043,1044^ In the clinical study MONARCH-1, the safety and efficacy of abemaciclib monotherapy in patients with advanced breast cancer with refractory HR+, HER2− were evaluated. Results have shown that patients using abemaciclib have a significantly longer duration of response and PFS with milder adverse effects.^1045^ In a follow-up, a clinical study of abemaciclib in combination with fulvestrant or letrozole showed significant increases in PFS in the abemaciclib combination group compared with abemaciclib monotherapy group, and most patients were not severely neutropenic.^1044,1046^

Trilaciclib, a short-acting CDK4/6 inhibitor developed by G1 Therapeutics, has received approval from the FDA to decrease the incidence of chemotherapy-induced myelosuppression in adult patients when administered before a platinum/etoposide-containing regimen or topotecan-containing regimen for extensive-stage SCLC in 2021.^1047^ Other clinically CDK4/6 inhibitors have been developed including TQB3616, SPH4336, dalpiciclib (SHR6390), and flavopiridol (L86-8275). TQB3616, a novel CDK4/6 inhibitor, exhibits high selectivity and effectiveness in preclinical cancer models.^1048^ SPH3643, a highly selective CDK4/6 inhibitor, can efficiently and stably cross the blood‐brain barrier.^1049^ Dalpiciclib (SHR6390), a novel, highly selective CDK4/6 inhibitor, reveals high activity with IC_50_ of 12.4 nM and 9.9 nM against CDK4 and CDK6, respectively.^1050^ Flavopiridol, a pan-CDK inhibitor originally purified from Dysoxylum binectariferum,^1051^ is the first CDK inhibitor entering clinical trials and can target CDK1, CDK2, CDK4, CDK6, and CDK7.^1052^

#### MDM2 inhibitors

Since the first discovery of the co-crystal structure of MDM2-p53 complex in 1996,^1053^ an increasing number of researches have been dedicated to uncovering its functions in tumors.^1054^ Gene amplification, increased transcription, and accelerated translation cause the aberrant elevation of MDM2, which promotes p53 ubiquitination and increases p53 degradation. Thus, targeting MDM2-p53 interaction is a particularly attractive therapeutic strategy for p53 reactivation in tumors.^1054,1055^ Numerous small-molecule MDM2 inhibitors have been discovered so far, and nine of them, including RG7112, idasanutlin, AMG-232, SAR40583, APG-115, NVP-CGM097, siremadlin, and MK-8242, and milademetan, have undergone or been undergoing clinical trials for the treatment of cancers.^1028^

Nutlins, including Nutlin-1, Nutlin-2, and Nutlin-3, are the first selective and potent MDM2 inhibitors synthesized in 2004,^1055^ which lay the foundation for the following development of MDM2 inhibitors. RG7112, an MDM2 inhibitor developed by Roche based on the structure of Nutlins, can bind to the p53 pocket on MDM2 and suppress the p53-MDM2 interaction, and is the first MDM2 inhibitor to be assessed in clinical trials.^387,1054^ RG7112 has demonstrated clinical activity in a phase I trial for the treatment of patients with leukemia.^1056^ However, the poor tolerability and the adverse events, such as gastrointestinal toxicity, myelosuppression, sepsis, and hemorrhage, hinder its further developmen.^387,1054,1056^ Subsequently, idasanutlin (RG7388), another highly potent and selective MDM2 antagonist, exhibits superior potency, selectivity, and bioavailability compared with RG7112, although sharing the same action mechanism.^1057^ Idasanutlin is the second MDM2 inhibitor to be evaluated in clinical trials.^387^ The most common adverse events of idasanutlin include gastrointestinal toxicity (diarrhea and nausea) and hypokalemia.^1054^ A phase Ib study of idasanutlin in combination with XPO1 inhibitor selinexor for the treatment of children with progressive or recurrent atypical teratoid/rhabdoid tumors and malignant rhabdoid tumors is ongoing (NCT05952687). Meanwhile, a phase I/II study of idasanutlin monotherapy or in combination with chemotherapy or venetoclax for the treatment of acute leukemias or solid tumors is recruiting (NCT04029688). In addition, AMG-232 (navtemadlin, KRT-232) is an investigational oral, selective MDM2 inhibitor. Its most common adverse events include nausea, diarrhea, vomiting, decreased appetite, anemia, thrombocytopenia, and leukopenia.^1058^ Currently, five clinical trials of AMG-232 monotherapy or in combination with other drugs for the treatment of patients with cancers are recruiting (NCT03031730, NCT03041688, NCT03107780, NCT03217266, and NCT04190550).

Furthermore, SAR405838(MI-77301) and APG-115 are all spirooxindole-based MDM2 inhibitors developed by Wang Shaomeng’s research group.^387,1059^ APG-115 is designed to overcome stability-related issues observed in SAR405838.^1054,1060^ APG-115 can strongly bind with MDM2 protein, and has good chemical stability and excellent oral pharmacokinetic parameters.^387^ A phase I study of SAR405838 in patients with advanced cancer has been completed (NCT01636479), but no result has been posted. Significantly, seven clinical trials of APG-115 monotherapy or in combination with other drugs for the treatment of patients with tumors are recruiting. NVP-CGM097(CGM097), a highly potent and selective MDM2 inhibitor, has good cell activity, metabolic stability, and PK parameters.^1061^ A phase I study of CGM097 in patients with advanced solid tumors with p53 wild-type status has been completed with no results posted (NCT01760525). Siremadlin (HDM201) is an orally bioavailable and selective inhibitor of the p53-MDM2 interaction designed by Novartis.^1062^ Currently, three clinical trials of siremadlin monotherapy or in combination with other drugs for the treatment of patients with advanced soft-tissue sarcoma (NCT05180695), AML (NCT05447663, NCT05155709) are ongoing. MK-8242 (SCH900242) is a potent, orally bioavailable, small-molecule inhibitor of the MDM2-p53 interaction. Its common adverse events include anemia, leukopenia, pancytopenia, nausea, hyperbilirubinemia, hypophosphatemia, and anorexia.^1063^ A phase I trial of MK-8242 in patients with refractory/recurrent AML has been completed.^1064^ Furthermore, Milademetan (DS3032b) is an orally active MDM2 inhibitor by disrupting the MDM2-p53 interaction.^1065^ A phase I study of milademetan in combination with low-dose cytarabine with or without venetoclax for the treatment of AML has been completed, and has revealed modest therapeutic responses with recognizable gastrointestinal toxicity.^1065^

Several MDM2 inhibitors are currently being evaluated clinically for cancer therapy. However, there is no MDM2 inhibitor approved for clinical application.^387^ Challenges such as acquired resistance and toxicity remain. In addition, MDM2 PROTAC degraders have received heightened attention in recent years with higher cancer therapeutic efficacy, and their safety needs further determination^1066^.

### Targeting enabling replicative immortality

Studies have illustrated that the protection of telomeres at the ends of chromosomes is essential for the replicative immortality ability of tumor cells. Tumor cells avoid senescence or apoptosis by upregulating telomerase expression to maintain telomeric DNA length. Therefore, targeting telomerase is of great importance. Various telomerase targeting strategies have been developed, such as vaccines, antisense oligonucleotides, and small molecule inhibitors.^390,1067^

#### Telomerase inhibitors

Given the critical role of telomere length in tumor proliferation, targeting telomerase is a promising antitumor treatment approach.^392^ It has been observed that the initial telomere length affects the therapeutic effect of telomerase inhibitors.^1068^ Therefore, it is valuable to detect telomerase length during clinical telomerase-targeted therapy.^1069^

The key goals of antitelomerase therapy are to induce tumor cell death and to reduce normal cytotoxicity. Numerous telomerase therapeutic options inhibit hTERT or hTR activity.^390,1067^ Imetelstat (GRN163L), developed by Geron, is a competitive inhibitor of telomerase that binds to the nucleotide region of the telomerase holoenzyme at a specific active site with high affinity, resulting in complete inhibition of telomerase activity.^390^ It has been identified that imetelstat has inhibitory effects on a series of cancer cells, including breast cancer,^1070^ lung cancer,^1071^ prostate cancer,^1072^ pancreatic cancer,^1073^ osteosarcoma,^1074^ glioblastoma,^1075^ HCC, and bladder cancer.^390^ In August 2023, Geron corporation announced the FDA acceptance of a new drug application for imetelstat for the treatment of transfusion-dependent anemia in patients with lower-risk myelodysplastic syndromes (MDS). Imetelstat is currently in a phase II clinical trial to evaluate its efficacy and safety in participants with high-risk MDS or AML that is relapsed/refractory to hypomethylating agent treatment (NCT05583552).

In addition, an increasing number of studies have identified telomerase as a promising anticancer immunotherapy target, and telomerase-based immunotherapy is a potential antitumor therapeutic strategy.^1076^ Antitelomerase immunotherapy exerts its antitumor effects mainly by enhancing the sensitivity of the immune system to tumor cells expressing telomerase-specific antigenic epitopes, thereby activating hTERT-specific CD8+ cells. The protein fragments or peptides formed by telomerase degradation in tumor cells are expressed on the surface of tumor cells as tumor-associated antigens via the human leukocyte antigen (HLA) class I pathway, which in turn triggers antitumor cytotoxic T lymphocyte responses.^390^ CD4+ or CD8+ cytotoxic T lymphocytes can target telomerase-specific antigenic epitopes to kill tumor cells.^1076^ Current telomerase-based cancer immunotherapy mainly includes the hTERT vaccine and dendritic cell strategy. Immunotherapy-based hTERT peptide (GV1001), cryptic peptides (Vx001), and dendritic cells (GRVAC1), three promising telomerase-targeted vaccines with low toxicity to normal cells and no autoimmunity,^390^ have been used for antitelomerase immune response therapy in cancer patients.^1077^ GV1001 is a 16 amino-acid hTER peptide vaccine containing the hTERT active site which significantly activates CD4+ or CD8+ cytotoxic T lymphocyte responses.^1078^ Vx001 is a vaccine containing the hTERT amino acid sequence with a high affinity for HLA class I. GRNVAC1, a dendritic cell-based vaccine with good activity and tolerance, exerts the antitumor effect by stimulating lysosomal degradation of hTERT into small peptide.^390^ Currently, GV1001, GRNVAC1, and Vx001 vaccines are in clinical trials. A phase II clinical trial (NCT00510133) of active immunotherapy with GRNVAC1 in patients with acute myelogenous leukemia has been completed, but no results have been posted.

Although targeting telomerase is an attractive antitumor strategy, some limitations still exist. First, as telomere shortening induced by cell division is a slow and long-term process, therapies that inhibit telomerase activity are hysteretic and are not suitable for first-line cancer therapy. Second, since telomerase is expressed in highly proliferative cells such as hematopoietic precursor cells and epidermal stem cells, telomerase targeting therapies are potentially toxic and cause side effects. Third, preclinical telomerase targeting therapy lacks a suitable model for evaluation.^1068^ It has been observed that mouse telomeres are 5–10 times longer than human,^1068,1079^ and their telomerases are widely expressed at low levels in adult tissue.^1080^ Thus, the dependence of mice on replicative immortality induced by telomerase activation is much lower than that of humans. Fourth, the structure characteristics of human telomerase holoenzymes need to be further resolved, which is crucial for telomerase inhibitor development.^1068^

### Antiangiogenesis therapy

The concept of “antiangiogenesis” therapy was proposed by Dr. Judah Folkman in 1971, who found that tumor growth requires neovascularization for maintenance.^1081^ Antiangiogenic therapy has become an invaluable strategy to hinder tumor proliferation and metastasis. The current antiangiogenic inhibitors mainly include VEGF inhibitors, FGF inhibitors, and PDGF inhibitors.

#### VEGF inhibitors

Antiangiogenic therapies target angiogenesis via two major mechanisms: blocking intracellular receptor tyrosine kinases or neutralizing angiogenic factors such as VEGF or its receptor. The current drugs targeting VEGF include mAbs, VEGF decoy receptors, and small molecule TKIs. These drugs can be used as monotherapy or in combination with other chemotherapeutic agents for clinical treatment.^401^

In 2004, the anti-VEGF-A humanized mAb bevacizumab (avastin), the first antiangiogenic agent, was first approved by the FDA for the treatment of advanced CRC. In May 2009, bevacizumab was approved as a second-line cancer therapy for the treatment of glioblastoma. Subsequently, in July 2009, the FDA approved bevacizumab in combination with interferon-alpha for the treatment of RCC.^401^ Currently, combination chemotherapy is used for treating patients with advanced CRC, NSCLC, and breast cancer.^398,1082^ Ramucirumab (cyramza), a mAb that binds VEGFR2 to block the VEGF signaling pathway, has been approved for the treatment of many types of solid tumors, such as advanced or metastatic gastric cancer, gastroesophageal junction adenocarcinoma, uterine cancer, CRC, and ovarian cancer.^425,1083,1084^ Aflibercept, also known as VEGF-Trap, is a soluble recombinant fusion protein consisting of the extracellular binding domains of VEGFR-1 and VEGFR-2, which was approved by the FDA in 2012 for the treatment of metastatic CRC.^1084^ Aflibercept interacts with circulating VEGF, thus preventing its binding to receptors on endothelial cells.^1085^ There are clinical studies using aflibercept in combination with the chemotherapeutic agent for the treatment of various malignancies, such as patients with advanced CRC.^401^ In addition, ziv-aflibercept was approved for the treatment of patients with metastatic CRC in combination with FOLFIRI (folinic acid, fluorouracil, and irinotecan) in 2012.^425,1086^

Since angiogenesis contains multiple signaling pathways, such as the VEGFR family and FGFR family, selective antiangiogenic agents especially TKIs can minimize the induction of toxicities.^1087^ Compared with macromolecules, small molecule TKIs possess multitarget inhibitory efficiency, and penetrate into cells easily due to hydrophobic properties, thus blocking the activation of various signaling pathways, which ultimately increases treatment efficiency.^1087^ In addition, small molecule TKIs can be administered orally as inhibitor salt form.^1087^

Sorafenib (BAY43-9006) is a selective inhibitor targeting VEGFR-2 and VEGFR-3, PDGFR, FMS-like tyrosine kinase 3 (FLT-3), and c-Kit^401^ and is the first TKI approved by the FDA as a first-line treatment for advanced HCC.^1088^ A phase III clinical trial uncovered significant improvements in OS in patients with advanced HCC treated with sorafenib.^1089^ Common clinical adverse effects of sorafenib include diarrhea, fatigue, skin reactions on the hands and feet, rash or desquamation, and anorexia.^416^ Lenvatinib, an oral TKI that targets VEGFR, FGFR, PDGFR, KIT, and RET, exerts antiproliferative and immunomodulatory activity in preclinical cancer models, and has been approved as a first-line treatment for HCC. Compared with sorafenib, levatinib has improved OS and PFS in patients with HCC.^1090^ In addition, lenvatinib suppresses the development of various cancers by inhibiting VEGFR signaling, such as kidney cancer,^1091^ pancreatic cancer,^1092^ SCLC,^1093^ breast cancer,^1094^ differentiated thyroid cancer,^1095^ and anaplastic thyroid cancer.^1090^ The most common adverse effects of lenvatinib treatment include hypertension, diarrhea, loss of appetite, weight loss, and fatigue.^416^ Sunitinib, an oral TKI targeting VEGFR1-3, PDGFR, FLT-3, and c-Kit,^401,1085^ is approved by the FDA for the treatment of refractory GIST and metastatic RCC.^401^ There are clinical studies using sunitinib for the treatment of advanced or metastatic breast cancer and NSCLC.^401^ Pazopanib (GW786034) has been approved for treating advanced RCC. Its common adverse effects are diarrhea, hypertension, hair color change, nausea, anorexia, and a 2% occurrence of myocardial infarction or ischemia. Axitinib, a second-generation inhibitor of VEGF-1, 2, and 3, is more selective for VEGF without blocking PDGF, B-RAF, FLT-3, and KIT targets. In January 2012, axitinib was approved by the FDA for the treatment of RCC after failure of a prior systemic therapy.^401^

Despite the emerging number of antiangiogenic therapeutic agents, challenges remain to be solved in antiangiogenic drug development and application.^398^ First, the tumor vascular system in preclinical mouse models is more sensitive to antiangiogenic therapy than the human tumor vascular system, which enhances the inconsistency between preclinical and clinical practice.^398^ Second, the effect of antiangiogenic monotherapy is limited, and the appropriate strategy for the combination of antiangiogenic drugs with other drugs needs to be discovered. For example, immune checkpoint inhibitors and antiangiogenic agents have been proven to be a promising therapeutic strategy. Antiangiogenic agents can reshape the immune TME, while immune checkpoint inhibitors can downregulate vascular endothelial growth factor expression and alleviate hypoxic conditions to combat angiogenesis.^1096^ Moreover, withdrawal of antiangiogenic drugs may result in tumor growth and metastasis, and the strategy to prevent this rebound needs to be developed.^1097^ In conclusion, tumor angiogenesis is essential for tumor growth. The regulatory factors of tumor angiogenesis and targeted therapies are diverse and need to be further elucidated in the future.

#### FGF/FGFR inhibitors

Studies have found that abnormal FGF signaling contributes to tumor angiogenesis and subsequently promotes tumor growth.^422^ Researches on FGF-targeted therapies have flourished in recent years, but no FGF-targeted therapy has been approved for cancer treatment. Current FGF-targeted therapies include multitargeted small molecule TKIs, selective FGFR-targeted TKIs, mAbs against FGFR, and FGF ligand traps.^1098,1099^

Dovitinib (TKI258) is an orally active and nonselective TKI that targets VEGFR1-3, FGFR1-3, and PDGFR.^1100^ It has good antitumor activity against RCC^1101^ and breast cancer.^1100^ Clinical trials evaluating the efficacy and safety of dovitinib alone or in combination with other anticancer drugs in patients with solid tumors and hematologic malignancies are ongoing. Other nonselective multitargeted anti-FGFR TKIs include nintedanib (BIBF1120),^1102^ lucitanib (E3810),^1103^ and ponatinib (AP24534),^1104^ which reveal antitumor activity in advanced solid tumors. Given the toxicity of multitargeted TKIs, several FGFR-selective inhibitors have been developed that hinder FGFR1-3 kinase activity to varying degrees and are currently in clinical trials. Infigratinib (NVP-BGJ398), an oral ATP-competitive FGFR inhibitor developed by QED Therapeutics, was granted accelerated approval by the FDA on May 28, 2021, for the treatment of adults with previously treated, unresectable locally advanced or metastatic cholangiocarcinoma with an FGFR2 fusion or other rearrangement.^1105^ In addition, infigratinib is currently in a phase I clinical trial for renal pelvis and ureter urothelial carcinoma (NCT04228042), and phase II clinical trials for gastric cancer (NCT05019794), advanced solid tumors (NCT04233567), cholangiocarcinoma (NCT04233567), and metastatic and refractory malignant solid neoplasm (NCT04233567) treatment. Erdafitinib (JNJ-42756493), an FGFR inhibitor developed by Janssen Pharmaceutical, was granted accelerated approval by the FDA on April 12, 2019, for the treatment of patients with locally advanced or metastatic urothelial carcinoma with susceptible FGFR3 or FGFR2 genetic alterations.^1106^ Erdafitinib is currently in a phase II clinical trial for urinary bladder neoplasms (NCT04172675) and advanced solid tumor (NCT04083976) treatment. Moreover, futibatinib (TAS120), an oral, covalently binding, irreversible inhibitor of FGFR1-4, is being developed by Taiho Oncology and Taiho Pharmaceutical for the treatment of various cancers.^1107^ On September 30, 2022, the FDA granted accelerated approval to futibatinib for the treatment of adult patients with previously treated, unresectable, locally advanced or metastatic intrahepatic cholangiocarcinoma harboring FGFR2 gene fusions or other rearrangements.^1108^ Futibatinib is currently in a phase II clinical trial for the treatment of patients with metastatic breast cancer (NCT04024436), advanced and metastatic HCC (NCT04828486), and advanced and metastatic urothelial cancer (NCT04601857), phase I and II clinical trials for NSCLC (NCT04965818), and a phase III clinical trial for soft tissue sarcoma (NCT03784014). In addition to small molecules, there are few clinical anti-FGFR mAbs. MGFR1877S is an anti-FGFR3 mAb that is being studied in a phase I clinical trial in patients with solid tumors (NCT01363024). FP-1039 (GSK3052230) is a soluble fusion protein that belongs to an FGF ligand trap that isolates the FGF ligand and prevents its binding to the receptor.^1099^ FP-1039 was studied in a phase I trial in patients with advanced tumors (NCT00687505) and a phase I trial was withdrawn in endometrial cancers with FGFR2 mutations (NCT01244438) due to substandard recruitment of patients.

Although there are numerous inhibitors and antibodies targeting FGF/FGFR under investigation, the development of anti-FGF/FGFR agents remains a tough challenge. Tumors with higher FGFR amplification copy numbers are more sensitive to FGFR inhibitors, whereas the fraction of patients with high FGFR amplification is not frequent. Thus, monitoring the status of FGFR and choosing a subgroup of patients could be beneficial for FGRF-targeted therapy.^438,1098^ The precise treatment for patients with tumor heterogeneity and specific FGFR abnormalities remains an urgent task.^1098^ Moreover, the side effects and toxicity of nonselective FGFR inhibitors remain an unresolved challenge.^1109^

#### PDGF/PDGFR inhibitors

PDGF is an essential angiogenic factor and its expression is upregulated in a diverse range of tumor cells. Therefore, blocking PDGF/PDGFR signaling is a promising strategy for targeted therapy.

##### Targeting PDGFR

PDGFR-targeted therapies include specific inhibitors, nonspecific inhibitors, mAbs, and RNA aptamers, among which inhibitors have been extensively studied. PDGFR inhibitors are classified into two categories including specific and nonspecific inhibitors, based on their binding properties to the receptor, and many PDGFR inhibitors are currently used in the clinical treatment of different cancers.^443^ CP-673451, a specific ATP-competitive PDGFR inhibitor, effectively inhibits PDGFRβ activity.^1110^ CHMFL-PDGFR-159 is a highly selective inhibitor of PDGFRα, which significantly inhibits the proliferation of chronic eosinophilic leukemia cells.^1111^ In addition, there are a large number of nonspecific PDGFR inhibitors. As tyrosine kinases have a conserved ATP-binding pocket, most small molecule TKIs are in a multitarget binding mode and inhibit both PDGFRα and PDGFRβ activity, including imatinib, ponatinib, sorafenib, nilotinib, nintedanib, dasatinib, midostaurin, ripretinib, sitravatinib, masitinib, sunitinib, axitinib, pazopanib, crenolanib.^1112^ Imatinib, the milestone in the history of TKI development, is a multitarget TKI that targets PDGFR signaling^1112^ and is used for the first-line treatment of patients with BCR-ABL-positive leukemia.^443^ Sunitinib (SU11248) is a multitarget inhibitor that targets PDGFR, VEGFR, c-Kit, FLT-3, and Ret kinase,^1113^ which was approved by the FDA in 2006 for the first-line treatment of metastatic RCC.^1114^ Sorafenib is an inhibitor targeting PDGFR, c-Kit, FLT-3, and VEGFR for the treatment of patients with advanced HCC.^443,1115^ Ponatinib (AP24534) is a multitarget TKI targeting BCR-ABL, PDGFR, VEGFR, FGFR, and Src, which has been applied as a third-line agent for CML treatment.^443^ Regorafenib is a multitargeted TKI that suppresses PDGFR, VEGFR, c-Kit, BRAF, as well as EGFR, and ERK^1116^ for the treatment of advanced HCC, CRC, and GIST.^443,1116,1117^ Nilotinib is a TKI that blocks PDGFR α/β, c-Kit, and is primarily used in the treatment of patients with CML and ALL.^443,1118^ Nintedanib is an inhibitor that blocks PDGFRα/β, VEGFR1-3, and FGFR1-3.^443^ Dasatinib is a multitarget inhibitor that targets PDGFR α/β, BCR-ABL, YES, and c-Kit and is approved by the FDA for the treatment of chronic granulocytic leukemia.^443,1119^ Midostaurin (PKC412) blocks PDGFR, Kit, VEGFR2, and PKCα, and is primarily used in the treatment of AML with FLT-3 mutations.^443,1120,1121^ Ripretinib (DCC-2618) inhibits PDGFRα/β, VEGFR2, and Kit activity and is approved for the treatment of adult patients with advanced GIST.^443,1122^ Sitravatinib (MGCD516) and masitinib (AB1010) broadly target the PDGFR family.^443^ Axitinib is also a multitarget inhibitor targeting PDGFR, VEGFR1-3, and c-Kit, and is being used to treat patients with advanced RCC.^443,1123^ Pazopanib is an inhibitor that targets PDGFR, VEGFR, and c-Kit, and is useful in the treatment of RCC and soft tissue sarcoma.^443,1124^ Crenolanib (CP-868596) inhibits PDGFRα/β, FLT-3, and c-Kit and is a highly selective inhibitor of PDGFRβ. Moreover, lenvatinib and avapritinib are both oral inhibitors that target PDGFRα and c-Kit.^443^ Although a large number of TKIs targeting PDGFR have been approved for clinical practice, their low specificity and activity against PDGFR make accuracy evaluation of anti-PDGFR effect difficult. Moreover, the side effects and increased toxicity of PDGFR inhibitors induced by multitargets or in combination with other anticancer drugs are big issues and cannot be ignored. It is worthwhile to explore the structure-function relationship of PDGF inhibitors in order to discover PDGF/PDGFR inhibitors with high specificity and selectivity in the future. The nanotechnology may provide new perspectives for PDGF/PDGFR-targeted therapy by enhancing target specificity and drug delivery accuracy.^443^

Compared with extensive progress that has been made in TKIs targeting PDGFR, the progress on mAbs targeting PDGFR is limited. Olaratumab (IMC-3G3), a high-affinity human anti-PDGFRα mAb,^1125^ is currently in a phase I clinical trial for soft tissue sarcoma (NCT03126591). Tovetumab (MEDI-575) is also a human anti-PDGFRα mAb that is well tolerated and has a favorable pharmacokinetic profile in patients with advanced solid tumors.^443^ Gint4.T, a high-affinity RNA aptamer that specifically binds to PDGFRβ,^1126^ inhibits TNBC lung metastases^1127^ and glioma.^443^ The high specificity of mAbs offers a significant opportunity to selectively target PEGFR resulting in specific therapeutic effects, which warrant further investigation.

##### Targeting PDGF

Compared with the extensive studies targeting PDGFR, only a few studies are reporting PDGF targeting therapy. MOR8457 is a high-affinity PDGF mAb^1128^ that selectively binds PDGF-BB and masks its receptor PDGFRβ-binding epitope, thereby blocking receptor dimerization and tyrosine kinase activation, ultimately effectively preventing PDGF-BB-induced cell proliferation.^443^ 6B3, a highly selective mAb, blocks PDGF-CC-induced PDGFRα phosphorylation and activation.^1129^ AX102 is a high-affinity single-stranded DNA aptamer that blocks PDGF-B and prevents activation of downstream proliferative signals.^443,1130^ E10030 is a PEG-modified aptamer that specifically binds to and restrains the function of PDGF-B.^425^

In summary, antiangiogenic drugs offer hope for antitumor therapy, but their resistance remains a pressing clinical issue. There are various reasons for antiangiogenic drug resistance. It has been revealed that lack of VEGF or VEGFR in certain metastatic tumors leads to poor efficacy in VEGF-targeted therapy, e.g., blocking the VEGFR2 pathway inhibits the growth of human RCC RBM1-IT4 cells implanted in the kidney but not in the bone of nude mice.^1131^ Tumor cells maintain sustained growth through existing blood vessels in organs with rich vascular systems (e.g., lungs), thus developing drug resistance.^1132^ Moreover, The abnormal induction of various proangiogenic factors such as bFGF,^406^ circulating PlGF, VEGF,^1133^ and FGF,^1134^ can cause acquired resistance to antiangiogenic therapy.^406^ In addition, the vascular dependence of tumor cells is heterogeneous and variable. Tumors with p53 mutation are less dependent on vascular supply and are more resistant to antiangiogenic drug treatment.^1135^ Therefore, an in-depth investigation into the mechanisms of antiangiogenic drug resistance and tumor heterogeneity is a promising strategy for tumor treatment.

### Targeting resisting cell death

#### Autophagy inhibitors/inducers

The multiple steps of the autophagic pathway present many opportunities for the development of targeted autophagy inhibitors, which currently include small molecule autophagy inducers and autophagy inhibitors, the latter consisting of inhibitors of autophagy initiation, autophagosome maturation, and lysosomal activity, hijacking the autophagosome and lysosome for targeted protein degradation^1136,1137^ (Table 4).

**Table 4:** The FDA-approved and clinically developed autophagy inhibitors/ inducers in cancer therapies

Drug	Highest phase	Indications	Identifier	Status
Rapamycin	IV	Non-hodgkin’s lymphoma	NCT01180049	Completed
	IV	Angiomyolipoma	NCT01217125	Completed
	IV	Refractory solid Tumors	NCT02688881	Completed
	IV	Hemangioendothelioma of liver	NCT04406870	Not yet recruiting
Everolimus (RAD001)	Approved	Progressive, well-differentiated non-functional, neuroendocrine tumors of gastrointestinal or lung origin with unresectable, locally advanced or metastatic disease	Novartis	/
	Approved	Advanced renal cell carcinoma following one prior antiangiogenic therapy	Novartis	/
	Approved	Advanced hormone receptor-positive, HER2-negative breast cancer in postmenopausal women	Novartis	
AZD8055	I	Glioblastoma multiforme, anaplastic astrocytoma, anaplastic oligodendroglioma, malignant glioma, brainstem glioma	NCT01316809	Completed
	I	Cancer, solid tumors, advanced solid malignancies	NCT00973076	Completed
	I	Solid tumors	NCT00731263	Completed
	I	Advanced hepatocellular carcinoma, cancer	NCT00999882	Completed
Chloroquine (CQ)	Approved	Prophylactic treatment of malaria	Bayer	/
	II	Astrocytoma, grade IV, glioblastoma	NCT02432417	Not yet recruiting
Hydroxychloroquine (HCQ)	Approved	Chronic, discoid or systemic lupus erythematosus and acute or chronic rheumatoid arthritis	/	/
	II	Advanced cancer, pancreatic cancer	NCT04386057	Recruiting
	II	Melanoma	NCT04464759	Recruiting
	II	Breast cancer	NCT04841148	Recruiting
	II	Hepatocellular cancer	NCT03037437	Recruiting
	II	Breast cancer	NCT04523857	Recruiting
	II	Metastatic colorectal cancer	NCT05843188	Recruiting

Source: All the information is derived from ClinicalTrials.gov (https://www.clinicaltrials.gov) and the United States Food and Drug Administration.gov (https://www.fda.gov/)

The first category of autophagy inducers is small molecule compounds which act primarily by directly inhibiting mTORC1 or activating AMPK. The cellular energy state sensor AMP can inhibit biosynthesis in response to energy stress by suppressing mTORC1. Thus, inhibition of mTORC1 blocks phosphorylation of ATG13, ULK1, and ULK2 in the ULK1 complex, and promotes AMPK activation or RAPTOR phosphorylation, thereby increasing autophagic flux.^1136,1138^ Moreover, rapalogs induce autophagy by forming a complex with FK506-binding protein (FKBP12), acting as a metamorphic inhibitor of mTORC1.^1136,1139^ Rapamycin, Torin-1, and AZD8055 can selectively inhibit mTORC1 kinase activity.^1136,1140^ In addition, the sodium voltage-gated channel blocker carbamazepine and the L-type calcium channel blocker felodipine are mTORC1 nondependent autophagy inducers and have been approved by the FDA.^1136^ The covalent acrylamide-based autophagy inducer EN6 indirectly induces mTORC1 inactivation and increases lysosomal acidification, leading to enhanced autophagic flow.^1136^ Additionally, disaccharide trehaloses SMER-28 and BRD5631 can induce autophagy in an mTORC1-independent manner.^1141,1142^ The natural product OSW-1 induces autophagy by blocking oxysterol-binding protein to inhibit cholesterol transport to lysosomes, thereby inhibiting mTORC1.^1143,1144^ Mucolipin 1 (TRPML1), an activator of the transient receptor potential cation channel, induces autophagosome biogenesis.^1136,1145^

The second category is the inhibitors of autophagy initiation. Autophagy inhibitors targeting PI3K and ULK1 can inhibit autophagy by preventing autophagosome formation. The pan-PI3K inhibitors 3-methyladenine and wortmannin are widely used in autophagy studies.^1136^ VPS34 is an important component of the class III PI3K complex. Autophinib and cinchona alkaloid-derived azaquindole-1 are both potent inhibitors of VPS34,^1146,1147^ and SAR405 and VPS34-IN1 are better selective VPS34 inhibitors.^1136^ ULK inhibitors include MRT68921 and SBI-0206965,^1148,1149^ which selectively inhibit ULK1 and ULK2. In addition, the inhibitor ULK-101 is a more selective and active ULK1 inhibitor, and is currently considered to be the most promising ULK1 inhibitory tool compound.^1136^ The cysteine protease autophagin-1 (ATG4B), a key protein for autophagosome formation and maturation, cleaves the C-terminus of LC3B and then binds to PE via ATG3. The development of ATG4B inhibitors is an effective way to inhibit autophagy. The styrrylquinoline-derived ATG4B inhibitor LV-320 has cysteine protease selectivity.^1136,1150^ The fluoromethylketone-based peptidomimetic FMK-9a, an irreversible covalent inhibitor of ATG4B, effectively inhibits ATG4B protein hydrolysis activity. In addition, altering the lipid composition of the nascent phagophore is also a new strategy for the development of autophagy inhibitors. The cholesterol transport protein GRAMD1A can effectively inhibit autophagosome biogenesis.^1136^

The third category is inhibitors of autophagosome maturation and lysosomal activity. The calcium channel TRPML1 is essential for autophagosome formation, and its small molecule inhibitor ML-SI3 can inhibit autophagic flow.^1145,1151^ In addition, inhibition of autophagosome-lysosome fusion by enhancing enhancement of 20S proteasome activity is an effective strategy to reduce autophagic flux.^1152^ The small molecule TCH-165 activates the 20S proteasome and specifically degrades important autophagosome-lysosome fusion regulators. Targeting autophagic terminal lysosomal activity is an effective method to inhibit autophagic flow.^1136^ These inhibitors inhibit lysosomal acidification by inhibiting v-ATPase or by directly increasing lysosomal pH and promoting lysosomal hydrolase inactivation. v-ATPase is a multisubunit proton pump responsible for maintaining low lysosomal pH. Natural products have been a rich source of highly potent v-ATPase inhibitors, including the macrolide antibiotics bafilomycin A1, concanamycin, benzolactone enamides salicylihalamide A, and lobatamide.^1136,1153,1154^ Lys01 is a tenfold more active autophagy inhibitor than hydroxychloroquine (HCQ), and its water-soluble salt Lys05 effectively promotes lysosomal deacidification and inhibits the proliferation of multiple tumor cell lines in vitro and the growth of tumor xenograft models in vivo.^1155^ Other lysosomal inhibitors, such as quinacrine, VATG-027, and VATG-032, also showed antitumor activity. VATG-027 is a potent inhibitor of autophagy with high cytotoxicity.^1156^ To date, the lysosomotropic agents chloroquine (CQ) and HCQ have been the main clinically applied autophagy inhibitors and are commonly used to alleviate acute and chronic inflammatory diseases, although a variety of autophagy inhibitors have been developed.^1136,1157^ They block the fusion of autophagosomes with lysosomal fusion to block organelle and protein degradation processes, thereby inhibiting nutrient recycling.^460^ CQ can enter the lysosome as a freely diffusing lysosomotropic agent and is deprotonated and trapped inside as a diacidic base in the lysosome.^1156,1158^ By sequestering the free hydrogen ions required to maintain an acidic pH, CQ increases the basicity of the lysosome, which renders pH-dependent lysosomal hydrolases and proteases, blocks lysosomal turnover, and inhibits the final stage of autophagy.^1156^ CQ and HCQ have been extensively studied as safety autophagy distributors for the treatment of various cancers, including breast cancer, melanoma, lung cancer, multiple myeloma, glioma, kidney cancer, prostate cancer, CRC, and other advanced solid tumors.^1156,1159–1161^

The fourth category is the degradation of the autophagosome and lysosome for targeted proteins. PROTACs have been applied to selectively target the degradation of autophagosomes or lysosomes and have promising applications.^1136^

In conclusion, there are various action mechanisms of autophagy inhibitors, which increase tumor chemotherapy drug sensitivity and inhibit tumor cell proliferation and metastasis.^1159^ Small molecule autophagy inhibitors remain excellent tools for autophagy-targeted therapy with the advantages of easy administration, rapid onset of action, and mostly reversible.^1136^ Given the nonautophagic targeted effects of most current autophagic targets^1162^ and the existence of mitochondrial autophagy pathways, the development of highly selective autophagy inhibitors is crucial.^1136^

#### Antiapoptotic therapy

Controlling cancer growth by promoting apoptosis is an effective antitumor strategy, and various apoptosis-based targeted therapies for tumors have been developed. Currently, the main focus on inhibitor development is targeting antiapoptotic family members. The inhibitors against BCL-2 are the most extensively studied, which include oligonucleotides that target BCL-2 expression, and proapoptotic BH3 mimetics that bind to antiapoptotic BCL-2 members (Table 5).^463^

**Table 5:** The FDA-approved and clinically developed antiapoptotic inhibitors in cancer therapies

Targets	Drug	Highest Phase	Indications	Company/Identifier	Status
BCL-2	PNT-2258	II	Non-hodgkin’s lymphoma	NCT01733238	Completed
		II	Diffuse large B-cell lymphoma	NCT02226965	Completed
Dual BCL-2 and BCL-X_L_ inhibitors	Navitoclax (ABT-263)	II	Small cell lung cancer, Small cell lung carcinoma	NCT00445198	Completed
		II	Chronic lymphocytic leukemia	NCT01557777	Completed
		II	Metastatic malignant solid neoplasm, recurrent lung small cell carcinoma	NCT03366103	Terminated
		II	Refractory acute lymphoblastic leukemia, relapsed acute lymphoblastic leukemia	NCT05192889	Recruiting
		II	Metastatic malignant solid neoplasm, refractory malignant solid neoplasm, unresectable malignant solid neoplasm	NCT02079740	Active, not recruiting
		II	Malignant solid neoplasm, melanoma	NCT01989585	Active, not recruiting
		II	Chronic lymphocytic leukemia	NCT01087151	Completed
		II	Platinum-resistant or Refractory ovarian cancer	NCT02591095	Completed
		I/II	Chronic lymphocytic leukemia	NCT00481091	Completed
		I/II	Chronic lymphoid leukemia, follicular lymphoma, lymphoid malignancies, mantle cell lymphoma, non-hodgkin’s lymphoma, peripheral T-cell lymphoma	NCT00406809	Completed
		II	Prostate cancer	NCT01828476	Terminated
	Palcitoclax (APG-1252)	II	Small cell lung cancer	NCT04210037	Terminated
	ABT-737	/	Ovarian cancer	NCT01440504	Completed
	AZD0466	II	Hematological malignancies	NCT04865419	Recruiting
Selective BCL-2 inhibitors	Venetoclax (ABT-199)	Approved	Chronic lymphocytic leukemia and acute myeloid leukemia	AbbVie Inc. and Genentech Inc	/
	S55746 (S-055746, BCL201)	I	B-cell non-hodgkin lymphoma, chronic lymphocytic leukemia, multiple myeloma	NCT02920697	Completed
		I	Follicular lymphoma, mantle cell lymphoma	NCT02603445	Completed
		I	Acute myeloid leukemia, myelodysplastic syndrome	NCT02920541	Completed
	Lisaftoclax (APG-2575)	II	Chronic lymphocytic leukemia, small lymphocytic lymphoma	NCT05147467	Recruiting
		II	Multiple myeloma, amyloidosis	NCT04942067	Recruiting
		II	Breast cancer solid tumor, adult	NCT04946864	Recruiting
		II	Multiple myeloma	NCT04674514	Recruiting
		II	Chronic lymphocytic leukemia, small lymphocytic lymphoma	NCT04494503	Recruiting
		II	Relapsed/refractory acute myeloid leukemia, myeloid malignancy	NCT04501120	Recruiting
		I/II	Acute myeloid leukemia	NCT04964518	Recruiting
BCL-X_L_ inhibitors	ABBV-155	I	Advanced solid tumors	NCT03595059	Active, not recruiting

Source: All the information is derived from ClinicalTrials.gov (https://www.clinicaltrials.gov) and the United States Food and Drug Administration.gov (https://www.fda.gov/)

PNT-2258, a 24-base, single-stranded, chemically unmodified phosphodiester DNA oligonucleotide encapsulated in a specialized liposome, can target the regulatory region upstream of the BCL-2 gene.^1163^ Navitoclax (ABT-263) is the second-generation, potent, and orally bioavailable Bad-like BH3 mimetic with an oral bioavailability of 20 to 50% in preclinical models. ABT-263 disrupts the interaction of BCL-2/BC-X_L_ with pro-death proteins and induces BAX translocation, cytochrome c release, and ultimately apoptosis.^463,1164^ Navitoclax inhibits growth in multiple preclinical tumor models^1165^ and is currently being evaluated in combination with the PARP inhibitor olaparib for the treatment of TNBC and ovarian cancer in a phase I clinical trial (NCT05358639). Palcitoclax (APG-1252) is a dual BCL-2 and BCL-X_L_ inhibitor with safe and well-tolerated properties for treating patients with metastatic solid tumors.^1166^ ABT-737 is the first small molecule designed to selectively bind the hydrophobic pocket within BCL-2, BC-X_L_, and BCL-W, which is not bioavailable for oral administration. ABT-737 displaces BIM from BCL-2’s BH3-binding pocket, subsequently activates BAX and induces mitochondrial permeability, ultimately leading to apoptosis.^463,475,1167^ ABT-737 has antitumor activity against hematologic and solid tumors, including CLL, lymphoma, and SCLC.^475,1168,1169^ In addition, ABT-737 exerts synergistic cytotoxicity with chemotherapy and radiotherapy.^475^ AZD0466, a novel BH3-mimetic inhibitor targeting BCL-X_L_ and BCL-2, has potent antitumor activity in preclinical models of malignant pleural mesothelioma.^1170^

Venetoclax (ABT-199), a selective inhibitor of BCL-2, has been approved by the FDA for the treatment of CLL and AML.^1171^ Venetoclax inhibits the proliferation of BCL-2 overexpressing small lymphocytic lymphoma.^463^ Venetoclax in combination with the FLT-3 TKIs gilteritinib or sorafenib synergistically inhibited FLT-3/ITD mutant AML proliferation and promoted apoptosis.^1172^ The toxic reactions of venetoclax include mild diarrhea (52%), upper respiratory tract infection (48%), nausea (47%), and grade 3 or 4 neutropenia (41%).^1173^ S55746 (S-055746, BCL201) is an orally selective BCL-2 inhibitor and can effectively impair hematological tumor growth.^1174^ Lisaftoclax (APG-2575), a selective oral BCL-2 inhibitor, demonstrates potent antitumor activity in preclinical models of hematologic malignancy.^1175^

ABBV-155 is a first-in-class selective BCL-X_L_ inhibitor. ABBV-155 monotherapy or in combination with paclitaxel or docetaxel is currently in a phase I clinical trial in advanced solid tumors to assess its safety and preliminary activity (NCT03595059). In addition, as the BAX serine 184 regulatory site is responsible for subcellular localization and insertion into mitochondrial membranes, the agonists targeting BAX have been developed for cancer treatment. The small molecule BAX agonists SMBA1, SMBA2, and SMBA3 selectively bind to BAX and inhibit S184 phosphorylation, thereby promoting BAX insertion into mitochondrial membranes and the formation of BAX oligomers, and inducing conformational changes in BAX, ultimately leading to cytochrome c release and apoptosis.^1176^ Other BAX-activating compounds, such as BAM-7 and BTSA1, also have antitumor activity in glioblastoma and AML cells.^463,1177^ Meanwhile, some other preclinical inhibitors are also under investigation.^463^

Overall, apoptosis resistance is a hallmark of human cancers. The abnormal expression of antiapoptotic proteins and the downregulation or mutation of proapoptotic proteins promote the acquisition of apoptosis resistance in tumors.^491^ Tumor-targeted therapy inducing apoptosis is an effective approach to overcome apoptosis resistance and open up new directions for cancer treatment strategies.

#### Necroptosis-inducing anticancer agents

Various compounds and anticancer drugs with various mechanisms of action are capable of inducing necrosis in cancer cells, which include chemotherapeutic agents, natural compounds, and classical necrosis inducers.^578,1178^

Chemotherapeutic agents for necrosis have been widely developed in recent years. Necrostatin-1 is a small molecular alkaloid that was first considered an inhibitor of necrotic cell death in 2005^1179^ and was subsequently identified as a specific inhibitor of RIPK1. Necrostatin-1 blocks RIPK1 kinase activity by interacting with an essential structure for the death domain receptor engagement T loop and blocking RIPK1 kinase activity.^1180^ In addition, necrostatin-1 analogs were also effective in inducing necrotic cell death.^1181,1182^ BI2536, a small molecule inhibitor of mitotic kinase polo-like kinase 1, induces necroptotic cell death in prostate cancer cells.^1183^

Moreover, natural compounds also hold an essential place in necrosis-based cancer therapy. Shikonin, a naturally occurring naphthoquinone, triggers necrotizing cell death, circumvents drug transporter proteins, and antiapoptotic BCL-2 protein-mediated apoptosis resistance.^1184,1185^ Shikonin inhibits glioma cells,^1186^ primary osteosarcoma, and pulmonary metastatic osteosarcoma^1187^ by inducing necrosis. Shikonin analogs such as deoxyshikonin, acetylshikonin, isobutyrylshikonin, beta-dimethylacrylshikonin, isovalerylshikonin, and alpha-methyl-n butylshikonin are able to induce necrosis, thus overcoming tumor resistances which are mediated by the resistance factors such as P-gp, BCL-2 and BCL-X_L_, MRP1, and BCRP1.^1188^ Obatoclax (GX15-070) is an indole bipyrrole compound antagonizing BCL-2, BCL-X_L_, BCL-W, and MCL1, which triggers necrotizing cell death by promoting necrosomes on autophagosomal membranes. Obatoclax induces nonapoptotic forms of cell death in rhabdomyosarcoma cells.^1189^ In addition, polyphenon E(R), a natural product of green tea extract, induces endoplasmic reticulum stress and causes necrotic death of prostate cancer cells.^1190^ Staurosporines, isolated from the bacterium Streptomyces staurosporeus in 1977, are protein kinase inducers of the intrinsic apoptotic pathway and trigger necrosis in the leukemia cell line U937 when cystatin proteases are impaired.^1191,1192^ FTY720, a sphingolipid analog that mimics ceramide, induces necroptotic cell death by modulating lipid signaling in glioblastoma cells.^1193,1194^ 5′-Benzylglycinyl-amiloride (UCD38B) induces mitochondrial swelling, endoplasmic reticulum expansion, and nuclear condensation, and induces a nonapoptotic form of cell necrosis in glioma cells.^1195^

In addition to the above observations, other cell necrosis inducers are found to induce necrotic cell death via various mechanisms, such as selenosemicarbazone metal complexes,^1196^ protein disulfide isomerase PDIA6,^1197^ death receptor ligand TRAIL,^1198^ and mitochondrial activator of caspases mimetics (Smac).^1184^ Moreover, necrocytes can initiate adaptive immunity and recruit macrophages through activation of NF-κB, thereby activating immune cells and enhancing immunotherapy efficacy. The combined use of checkpoint inhibitors and necrocyte vaccines significantly improves the clinical outcomes of cancer patients.^566,1184^

### Targeting invasion and metastasis

There are still limitations and tough challenges in the targeted treatment of tumor metastasis. Tumor cells have already spread to the blood, bone marrow, and distant organs by the time some cancer patients are first diagnosed with tumors.^165,1199^ Therefore, antimetastatic therapies need to take into account not only cells that metastasize from the primary tumor site, but also the inhibition of cancer cells that have already spread. Currently, strategies for preventing metastasis have been demonstrated preclinically (Fig. 5). However, drug development has been hindered due to poor trial design and therapeutic strategies.^600^ Encouragingly, potent and specific MMP inhibitors are being developed that may further improve efficacy and attenuate toxicity.^1200^ In addition, targeted HGF/c-MET inhibitors are bringing light to tumor treatment.^677^ In conclusion, more than 90% of cancer mortality is now attributed to metastasis, and the prospect of targeted tumor metastasis therapy is unlimited.^165^

#### MMP inhibitors

Since cancer cells require MMPs to degrade collagen to promote cell metastasis, the first generation of MMP inhibitors is structural analogs of collagen, among which the first compound belongs to the hydroxamic acid zinc-binding group.^715,1201^ Developed by British Biotech, batimastat (BB-94) is a potent pan-MMP inhibitor that acts by chelating zinc ions at the active site of each MMP enzyme. As the first anti-MMP drug to be tested in clinical trials,^604^ batimastat effectively inhibits breast cancer, ovarian cancer, and CRC tumor growth and metastasis in vivo, although its poor solubility in water hampers its further development.^604,1202,1203^ The subsequent development of marimastat (BB-2516) is an orally available second-generation synthetic MMP inhibitor.^604^ Marimasta has been tested in phase III clinical trials to evaluate its effectiveness in patients with SCLC (NCT00003011), stage III NSCLC (NCT00002911), and metastatic breast cancer (NCT00003010), but no results have been disclosed. In addition, the bryostatins are naturally occurring macrocyclic actone products that inhibit MMPs by modulating upstream regulators of MMPs.^604^ Tanomastat (BAY12-9566), a bryostatin compound developed by Bayer, is a nonpeptide biphenyl MMP inhibitor that is effective against a wide range of tumors.^1204,1205^ However, a phase III randomized trial of tanomastat as maintenance therapy in patients with advanced ovarian cancer responsive to primary surgery and paclitaxel/platinum-containing chemotherapy has shown that tanomastat is generally well tolerated but had no impact on PFS or OS.^1206^ Other MMPs inhibitors such as COL-3 (NSC-683551),^1207^ neovastat (AE-941),^1208^ prinomastat (AG3340),^1209^ BMS-275291,^1210^ and metastat(COL-3),^1211^ have been evaluated in clinical trials (Table 6).

**Table 6:** The typical and clinically developed MMPs inhibitors in cancer therapy

Drug	Highest phase	Indications	Identifier	Status
COL-3 (NSC-683551)	I	Lymphoma, melanoma, neoplasm metastasis, renal cell carcinoma	NCT00001683	Completed
	I	Unspecified adult solid tumor, protocol specific	NCT00003721	Completed
Neovastat (AE-941)	III	Kidney cancer	NCT00005995	Completed
	III	Adenocarcinoma of the lung, adenosquamous cell lung cancer, large-cell lung cancer, squamous cell lung cancer, stage IIIA non-small cell lung cancer, stage IIIB non-small cell lung cancer	NCT00005838	Completed
Prinomastat (AG3340)	III	Lung cancer	NCT00004199	Completed
	III	Prostate cancer	NCT00003343	Completed
	II	Brain and central nervous system tumors	NCT00004200	Completed
Marimastat (BB-2516)	III	Lung cancer	NCT00003011	Completed
	III	Lung cancer	NCT00002911	Completed
	III	Breast cancer	NCT00003010	Completed
BMS-275291	II/III	Lung cancer	NCT00006229	Completed
Metastat(COL-3)	I/II	Brain and central nervous system tumors	NCT00004147	Completed

Source: All the information is derived from ClinicalTrials.gov (https://www.clinicaltrials.gov)

In conclusion, MMPs are a large family with different functions in tumor cells.^1212^ Depending on cell and tissue localization, disease type, etc., MMPs can be used as both drug targets and anti-targets.^715^ Therefore, given the cell or tumor specificity of MMPs, it is important to explore in-depth the contribution of MMPs in tumor progression and metastasis in various tumor types, which will facilitate the rational development of specific MMP inhibitors.^604^

#### HGF/c-Met inhibitors

There is an increasing number of studies on HGF/c-MET-targeted therapies, which mainly include anti-HGF/c-MET mAbs and small molecule inhibitors targeting the structural domain of c-MET kinase **(**Fig. 5 and Table 7). The mechanism of action of HGF/c-MET inhibitors is mainly neutralization or competition with HGF to inhibit receptor dimerization or induce c-MET degradation.^677^

**Table 7:** The clinically developed HGF/c-MET inhibitors

Type	Drug	Targets	Highest phase	Indications	Identifier	Status
Anti-c-MET mAbs	Emibetuzumab(LY2875358)	c-MET	II	Carcinoma, non-small-cell lung	NCT01900652	Completed
			II	Advanced cancer, gastric adenocarcinoma, gastroesophageal junction adenocarcinoma, hepatocellular cancer, renal cell carcinoma, non-small cell lung cancer	NCT02082210	Completed
	ABT-700	c-MET	I	Advanced solid tumors	NCT01472016	Completed
	Onartuzumab	c-MET	III	Solid tumor	NCT02488330	Completed
			III	Non-squamous non-small cell lung cancer	NCT01456325	Completed
			III	Gastric cancer	NCT01662869	Completed
			III	Non-squamous non-small cell lung cancer	NCT01887886	Completed
			III	Non-small cell lung cancer	NCT02031744	Completed
	LY2875358	c-MET	II	Gastric cancer	NCT01874938	Completed
			II	Non-small cell lung cancer	NCT01897480	Active, not recruiting
			II	Non-small cell lung cancer	NCT01900652	Completed
			I/II	Advanced cancer, gastric adenocarcinoma, gastroesophageal junction adenocarcinoma, hepatocellular cancer, renal cell carcinoma, non-small cell lung cancer	NCT02082210	Completed
Anti-HGF mAbs	Rilotumumab	HGF	III	Gastric cancer	NCT02137343	Terminated
			III	Gastric cancer	NCT01697072	Terminated
			III	Recurrent squamous cell lung carcinoma, stage IV squamous cell lung carcinoma	NCT02926638	Terminated
			II	Recurrent fallopian tube carcinoma, recurrent ovarian carcinoma, recurrent primary peritoneal carcinoma	NCT01039207	Completed
	Ficlatuzumab	HGF	II	Resistant, recurrent or metastatic head/neck squamous cell carcinoma	NCT03422536	Completed
			II	Non-small cell lung cancer	NCT02318368	Terminated
	YYB-101	HGF	Ib/IIa	Colorectal cancer	NCT04368507	Completed
Nonselective TKI (ATP-competitive)	Capmatinib	c-MET	IV	Non-small cell lung carcinoma	NCT05110196	Recruiting
	Tepotinib	c-MET	II	Non-small cell lung cancer	NCT03940703	Active, not recruiting
			II	Solid tumor, MET exon 14 skipping mutation, MET amplification	NCT04647838	Recruiting
			II	Advanced (Stage IIIB/IV) non-small cell lung cancer with MET exon 14 skipping alterations or MET amplification lung adenocarcinoma stage IIIB/IV	NCT02864992	Active, not recruiting
			II	Colorectal neoplasms	NCT04515394	Terminated
			II	Recurrent lung non-small cell carcinoma, stage IV lung cancer	NCT06031688	Not yet recruiting
			II	Gastric cancer, gastroesophageal-junction cancer	NCT05439993	Recruiting
			I/II	Advanced non-small cell lung cancer with MET mutations	NCT04739358	Recruiting
			I/II	Non-small cell lung cancer	NCT01982955	Completed
			I/II	Hepatocellular carcinoma	NCT02115373	Completed
			I/II	Hepatocellular carcinoma	NCT01988493	Completed
			II	Breast cancer, gastrointestinal cancer, non-small cell lung cancer, other cancer	NCT04591431	Active, not recruiting
	AMG-337	c-MET	II	Stomach neoplasms	NCT02016534	Terminated
			II	Solid tumor	NCT03147976	Withdrawn
			II	Clear cell sarcoma	NCT03132155	Terminated
			I/II	Stomach neoplasms	NCT02096666	Completed
Nonselective TKI (allosteric)	Tivantinib	c-MET	III	Hepatocellular carcinoma	NCT01755767	Completed
			III	Non-squamous, non-small cell lung cancer	NCT01244191	Terminated
			III	Non-small cell lung cancer	NCT01377376	Terminated
			III	Liver cancer	NCT02029157	Completed
Nonselective TKI (ATP-competitive)	Crizotinib	c-MET, ALK, RON, AXL, TIE2, ROS1	IV	Non-small cell lung cancer, anaplastic large-cell lymphoma, inflammatory myofibroblastic tumor	NCT05160922	Recruiting
			IV	Anaplastic lymphoma kinase or ROS1-positive non-small cell lung cancer	NCT03672643	Active, not recruiting
			IV	Systemic anaplastic large-cell lymphoma	NCT02487316	Withdrawn
	Cabozantinib	c-MET, c-RET, VEGFR1-3, c-Kit, FLT-3, TIE2, TRKB, AXL	IV	Hepatocellular carcinoma	NCT03963206	Completed
			IV	Medullary thyroid cancer	NCT01896479	Active, not recruiting
	Foretinib	c-MET, VEGFR2, RON, ERK, AKT, PDGFRβ, c-Kit, TIE2	II	Recurrent breast cancer	NCT01147484	Completed
			II	Neoplasms, head and neck	NCT00725764	Completed
			II	Carcinoma, renal cell	NCT00726323	Completed
			II	Neoplasms, gastrointestinal tract	NCT00725712	Completed
			I/II	Breast cancer	NCT01138384	Completed
			I/II	Lung cancer	NCT01068587	Completed
	Glesatinib	c-MET, AXL	II	Non-small cell lung cancer	NCT02954991	Completed
	Golvatinib	c-MET, VEGFR2, RON, Eph, c-Kit	I/II	Advanced solid tumors	NCT01433991	Terminated
			I/II	Platinum-resistant squamous cell carcinoma of the head and neck	NCT01332266	Completed
	Merestinib	c-MET, MST1R, FLT-3, AXL, MERTK, TEK, ROS1, NTRK1/2/3, DDR1/2, MKNK1/2	II	Carcinoma, non-small-cell lung, solid tumor	NCT02920996	Active, not recruiting
			II	Biliary tract cancer, metastatic cancer, advanced cancer	NCT02711553	Active, not recruiting

Source: All the information is derived from ClinicalTrials.gov (https://www.clinicaltrials.gov)

##### c-MET inhibitors

RTK is a cell surface receptor that binds to growth factor ligands such as HGF, VEGF, EGF, etc., and activates downstream signaling pathways. A number of small molecule TKI have been developed that selectively and nonselectively inhibit the catalytic activity of c-MET.^1213^ Significantly, two c-MET inhibitors, tepotinib developed by EMD Serono, and capmatinib developed by Novartis, have been approved by the FDA. Tepotinib inhibits MET kinase activity with an IC_50_ value of 1.7 nM and showed high selectivity for MET in screening against >400 kinases.^1214^ On February 3, 2021, the FDA granted accelerated approval to tepotinib for adult patients with metastatic NSCLC harboring MET exon 14 skipping alterations.^1215^ Capmatinib, an ATP-competitive, highly potent (IC_50_ value of 0.13 nM) and selective MET inhibitor, was granted regular approval by the FDA for the treatment of adult patients with metastatic NSCLC whose tumors have MET exon 14 skipping mutations in May 2020.^1216^ In addition, crizotinib (PF-02341066) is an orally bioavailable TKI that competitively inhibits the ATP-binding site of tyrosine kinases and inhibits c-MET, ALK, AXL and TIE2 activity.^679^ Crizotinib effectively inhibits c-MET phosphorylation and c-MET-dependent proliferation, migration, or invasion of tumor cells.^679,1217^ Notably, crizotinib has been approved for patients with ALK-positive and ROS1-positive metastatic NSCLC.^677^ Cabozantinib (XL184) is an orally available TKI that targets a variety of kinases including MET, VEGFR2, RET, FLT-3, and KIT.^1218^ It has shown efficacy in patients with prostate cancer^1219^ and advanced RCC.^1220,1221^ Foretinib (GSK1363089) is an oral and potent TKI targeting c-MET and VEGFR2. It binds to the ATP-binding pocket of the above kinases and makes kinase conformational changes. Foretinib has been tested in clinical trials in a variety of tumors including papillary renal cancer, gastric cancer, and head and neck cancer.^1222^ A phase II study of foretinib in adults with HNSCC revealed that 50.0% of participants have stable disease, and 21.4% have progressive disease (NCT00725764). Other phase II clinical trials of foretinib in solid tumors (NCT00742131) and breast cancer (NCT01138384) have been completed, but no results have been posted yet. Moreover, a phase II study of foretinib in adults with gastric cancer demonstrated that the serious adverse events of foretinib include abdominal pain (6.25%), dehydration (6.25%), malignant neoplasm progression (4.17%)(NCT00725712). Tivantinib (ARQ197) is a highly selective, non-ATP competitive inhibitor of c-MET.^1223^ Tivantinib inhibits the autophosphorylation of c-MET in many cancer cells and is highly selective for inactive or non-phosphorylated forms of c-MET, thus effectively blocking the activation of c-MET downstream effectors such as RAS, MAPK, and STAT3, ultimately inhibiting tumor proliferation, invasion and metastasis.^1222,1224^ Other nonselective c-MET inhibitors include glesatinib (MGCD-265), golvatinib (E-7050) and merestinib (LY-2801653), while selective c-MET inhibitors include tepatinib (EMD-1214063), AMG-337 and capatinib (INC-280), which are approved for clinical use or undergoing clinical study.

##### Anti-c-MET antibodies

Onartuzumab, an *Escherichia coli*-derived, humanized mAb against c-MET, can block the high-affinity binding of HGFα chain but not HGFβ chain to c-MET.^1225^ Preclinical studies found that onartuzumab effectively inhibits human glioblastoma and pancreatic cancer cell growth although further development of onartuzumab has been halted.^1226^ Emibetuzumab (LY2875358) is a humanized anti-c-MET bivalent antibody that effectively promotes internalization and degradation of c-MET, thereby blocking HGF-c-MET binding and HGF-induced c-MET phosphorylation.^1227^ Emibetuzumab combined with erlotinib is in a phase II clinical trial to evaluate their efficiency as a first-line treatment for patients with metastatic NSCLC with activated EGFR mutations (NCT01897480). LY3164530 is a bispecific anti-EGFR/c-MET antibody produced by fusing an anti-EGFR single-chain variable fragment to the N-terminal end of the emibetuzumab heavy chain.^1226^ LY3164530 disrupts signaling by binding and internalizing c-MET and EGFR.^1226^ Its phase I clinical trial in patients with advanced or metastatic cancer has been completed. However, significant toxicities associated with EGFR inhibition and the lack of a potential predictive biomarker limit its future development.^1228^ Amivantamab (JNJ-61186372) is a bispecific EGFR/c-MET antibody that binds the extracellular structural domain of each receptor, thereby avoiding resistance at the TKI binding site.^1229^ Amivantamab effectively inhibits tumors in various contexts, including tumors with T790M second-site resistance mutation in EGFR, c-MET pathway activation,^1230^ and EGFR exon 20 insertion driver mutations.^1229^ Amivantamab is currently being administered in monotherapy or combination with various drugs for cancer treatment in 15 clinical trials (NCT04077463, NCT02609776, NCT05845671, and NCT05653427). Encouragingly, amivantamab has been approved for marketing for the treatment of patients with metastatic NSCLC with EGFR exon 20 insertion mutations and platinum-based chemotherapy resistance (NCT04599712).

SAIT301 is a monoclonal humanized antibody developed by Samsung that promotes c-MET degradation.^1226,1231^ SAIT301 inhibits nasopharyngeal cell invasion and migration by downregulating EGR-1 via the degradation of c-MET.^1232^ Its clinical phase I trial in c-MET-overexpressed metastatic CRC has been completed, and the most common adverse effects were decreased appetite (50.0%), hypophosphatemia, fatigue, and dizziness (25.0%), diarrhea, and dyspnea (18.8%).^1233^ ABT-700 (h224G11) is a humanized bivalent mAb that inhibits c-MET dimerization and activation. A phase I clinical trial of ABT-700 in subjects with advanced solid tumors containing MET amplification or c-MET overexpression (NCT01472016) has been completed. ARGX-111 is an afucosylated IgG1 antibody that competitively binds c-MET, inhibits c-MET activity, and downregulates c-MET expression on the cell surface.^1234^ A clinical phase I trial of ARGX-111 in patients with advanced cancer overexpressing c-MET has been completed (NCT02055066). In addition. DN30 is a monovalent chimeric Fab that induces the cleavage of the extracellular portion of c-MET, leading to the shedding of its ectodomain.^1235,1236^ DN30 inhibits tumor growth in human gastric cancer, lung cancer, and glioblastoma.^1226^

##### Anti- HGF antibodies

Rilotumumab (AMG-102), an anti-HGF mAb binding to HGFβ chain structural domain, specifically blocks the activation of c-MET.^1237^ In particular, rilotumumab selectively alters the mature HGF, but shows no effect on the proteolytic activation process of pro-HGF.^1238^ To date, rilotumumab alone or in combination with other anticancer drugs, such as antiangiogenic agents, EGFR inhibitors, and chemotherapeutic agents, has been studied in clinical trials in patients with various solid tumors such as prostate cancer, kidney cancer, and advanced NSCLC.^677,1239^ Ficlatuzumab (AV-299), a humanized anti-HGF antibody, has been studied in clinical trials as a monotherapy or in combination with chemotherapeutic agents in patients with advanced pancreatic cancer (NCT03316599) and HNSCC (NCT02277197). YYB-101 is a humanized HGF antibody which inhibits c-MET activation by binding to the HGFα chain.^1226^ A clinical phase I trial of YYB-101 in patients with refractory advanced solid tumors (NCT02499224) has shown that YYB-101 exhibited favorable safety and efficacy in patients with refractory solid tumors. A clinical phase Ib/ IIa trial of YYB10 in combination with irinotecan in patients with metastatic or recurrent CRC (NCT04368507) has been completed, but no results are currently available.

##### Antibody mimetic engineered protein against HGF

MP0250, an ankyrin repeat protein capable of neutralizing VEGF and HGF, effectively inhibits multiple myeloma-mediated osteolysis and myeloma cell invasion.^1226^ Meanwhile, MP0250 can effectively improve bortezomib efficacy without increasing toxicity, suggesting that MP0250 combined with cytotoxic therapy may be a promising therapeutic approach.^1240^ A phase II clinical evaluation of MP0250 in combination with bortezomib and dexamethasone in patients with multiple myeloma (NCT03136653) has been completed, although the result has not yet been disclosed.

##### Competitive analogs of HGF

NK4 is a synthetic intramolecular fragment of HGF, originally purified as a fragment from elastase-digested HGF samples.^1241^ NK4 contains the HGF α-chain N-terminal hairpin domain and 4 kringle domains (K1–K4),^1242^ and lacks the 16 amino acids of the HGF C-terminus.^1226^ NK4 inhibits c-MET phosphorylation and activation by competing with HGF for binding to MET. NK4 effectively inhibits neovascularization, growth, invasion, and metastasis of many tumor cells.^1243,1244^

The HGF/c-MET pathway serves a critical role in cancer and is an attractive therapeutic target for cancer therapy. Over the past decade, great efforts have been devoted to the development of selectively c-MET inhibitors. Although small molecule c-MET inhibitors and antibody-based drugs have shown meaningful clinical efficacy, the challenges of resistance and side effects remain to be addressed. The c-MET amplification and overexpression, c-MET mutations, the activation of parallel signaling pathways, and the induction of HGF secretion are associated with acquired resistance after initial response to HGF/c-MET inhibitors. Therefore, how to overcome the acquired resistance as well as improve the safety of c-MET inhibitors needs to be solved urgently.^1245^ Moreover, stratifying patients appropriately based on the discovery of biomarkers may help identify the subgroups of patients who can benefit from anti- HGF/c-MET therapy.

### Targeting DDR pathways

Multiple DDRs related small molecule inhibitors have been approved for clinical use or are under clinical investigation, including PARP inhibitors, ATM inhibitors, ATR inhibitors, and CHK1 inhibitors (Table 8). Herein we mainly focus on the inhibitors of PARP, ATM, ATR, and CHK1.

**Table 8:** The clinically developed DDR inhibitors in cancer therapies

Type	Drug	Highest phase	Indications	Identifier	Status
ATM inhibitor	AZD0156	I	Advanced solid tumors	NCT02588105	Completed
	AZD1390	I	Recurrent glioblastoma multiforme, primary glioblastoma multiforme, brain neoplasms, malignant, leptomeningeal disease	NCT03423628	Recruiting
		I	Glioblastoma, glioma, glioblastoma multiforme, glioma, malignant	NCT05182905	Recruiting
		I	Solid tumor, metastatic solid tumor, solid carcinoma, solid tumor, adult, metastatic tumor, metastatic cancer	NCT05678010	Recruiting
		I	soft tissue sarcoma adult	NCT05116254	Recruiting
		I	non-small cell lung cancer	NCT04550104	Recruiting
	M4076	I	Advanced solid tumors	NCT04882917	Completed
		I	Metastatic or locally advanced unresectable solid tumors	NCT05396833	Recruiting
ATM and DNA-PKcs inhibitor	XRD-0394	I	Metastasis, locally advanced solid tumor, recurrent cancer	NCT05002140	Active, not recruiting
ATR inhibitor	ART0380	II	Advanced solid tumor, recurrent endometrial cancer, metastatic cancer	NCT05798611	Recruiting
		II	Advanced cancer, metastatic cancer, ovarian cancer, primary peritoneal cancer, fallopian tube cancer	NCT04657068	Recruiting
	ATRN-119	II	Advanced solid tumor	NCT04905914	Recruiting
	BAY1895344	I	Advanced solid tumor	NCT04095273	Completed
		I	Advanced solid tumor, non-hodgkin’s lymphoma, mantle cell lymphoma	NCT03188965	Recruiting
		I	Advanced solid tumors (excluding prostate cancer), ovarian cancer	NCT04267939	Recruiting
	Berzosertib (VX-970, M6620, VE-822)	II	Small cell lung cancer	NCT04768296	Completed
		II	Small cell lung cancer, small cell cancer, advanced solid tumor, high grade neuroendocrine cancers	NCT04802174	Recruiting
		II	small cell lung cancer, advanced solid tumors	NCT04826341	Recruiting
		II	Lung non-small cell squamous carcinoma, stage IV lung cancer	NCT04216316	Recruiting
		II	Leiomyosarcoma, adult	NCT04807816	Recruiting
		I	Advanced solid tumor	NCT05246111	Completed
		II	Castration-resistant prostate carcinoma, metastatic prostate carcinoma, stage IV prostate cancer	NCT03517969	Active, not recruiting
		II	Ovarian serous tumor, recurrent fallopian tube carcinoma, recurrent ovarian carcinoma, recurrent primary peritoneal carcinoma	NCT02595892	Active, not recruiting
		II	Bladder small cell neuroendocrine carcinoma, extensive stage lung small cell carcinoma, extrapulmonary small cell neuroendocrine carcinoma, limited stage lung small cell carcinoma, platinum-resistant lung small cell carcinoma, platinum-sensitive lung small cell carcinoma, prostate small cell neuroendocrine carcinoma, recurrent lung small cell carcinoma	NCT03896503	Active, not recruiting
		II	Metastatic malignant solid neoplasm, refractory malignant solid neoplasm, unresectable malignant solid neoplasm	NCT04266912	Active, not recruiting
		II	Metastatic bladder urothelial carcinoma, metastatic renal pelvis and ureter urothelial carcinoma, metastatic ureter urothelial carcinoma, stage IV bladder urothelial carcinoma	NCT02567409	Active, not recruiting
	Ceralasertib (AZD6738)	III	Advanced or metastatic non-small cell lung cancer	NCT05450692	Recruiting
	IMP9064	I	Solid tumor, advanced solid tumor	NCT05269316	Recruiting
	M4344	II	Ovarian cancer recurrent	NCT04149145	Withdrawn (never opened)
		I	Solid tumor, advanced solid tumor	NCT02278250	Completed
	RP-3500	II	Advanced solid tumor, adult	NCT04972110	Recruiting
		II	Solid tumor, metastatic cancer	NCT05566574	Recruiting
		I	Advanced solid tumor	NCT04855656	Recruiting
		II	Advanced solid tumor	NCT04497116	Recruiting
CHK1 inhibitor	Prexasertib (LY2606368)	II	Small cell lung cancer	NCT02735980	Completed
		II	Ovarian cancer	NCT03414047	Completed
		II	Triple-negative breast cancer	NCT04032080	Completed
		II	Advanced cancers	NCT02873975	Completed
		II	Desmoplastic small round cell tumor, rhabdomyosarcoma	NCT04095221	Active, not recruiting
		II	Platinum-resistant ovarian cancer, endometrial adenocarcinoma, urothelial carcinoma	NCT05548296	Recruiting
	SRA737	II	Advanced solid tumors or non-hodgkin’s lymphoma	NCT02797964	Completed
		II	Advanced solid tumors	NCT02797977	Completed
	LY2880070	II	Ewing sarcoma, Ewing-like sarcoma	NCT05275426	Recruiting
		II	Solid tumors, colorectal cancer, breast cancer, ovarian cancer, colon cancer, rectal cancer, neoplasms, endometrial cancer, soft tissue sarcoma, triple-negative breast cancer, pancreatic cancer	NCT02632448	Recruiting
		II	Castration-resistant prostate carcinoma, metastatic malignant neoplasm in the lymph nodes, metastatic prostate carcinoma, stage IV prostate cancer	NCT04071236	Recruiting
		II	Locally advanced rectal cancer	NCT03770689	Completed
		II	Cholangiocarcinoma, gallbladder carcinoma, malignant solid neoplasm	NCT04068194	Suspended

Source: All the information is derived from ClinicalTrials.gov (https://www.clinicaltrials.gov)

#### PARP inhibitors

PARP inhibitors are selective for targeting tumors deficient in the HR DNA repair factor BRCA1 or BRCA2 (BRCA1/2)^741^ or compromised HR.^733^ Six PARP inhibitors are currently approved for the clinical treatment of cancer patients including the specific subgroups with BRCA1/2 mutation: olaparib, rucaparib, niraparib, talazoparib, fuzuloparib, and pamiparib^733^ (Table 9). Olaparib is the first PARP inhibitor introduced into clinical practice.^1246^ In 2014, olaparib was approved for the treatment of patients with BRCA1/2-mutated metastatic ovarian cancer who had received three or more prior lines of chemotherapy. Subsequently, in 2016, rucaparib, a second PARP inhibitor, was authorized for the treatment of patients with advanced-stage ovarian cancers harboring deleterious BRCA1/2 mutations who had received two or more prior lines of chemotherapy. In 2019, niraparib was approved for the treatment of patients with HR-deficient advanced-stage ovarian cancers who had received three or more prior chemotherapy regimens.^1246^ Recently, in March 2022, olaparib was approved by the FDA for the adjuvant treatment of patients with hereditary BRCA1/2 mutations and HER2- high-risk early breast cancer^1247^ as well as for the maintenance treatment of patients with BRCA1/2-mutated ovarian cancers who are in complete or partial remission after platinum-based chemotherapy.^1248^ Moreover, PARP inhibitors are also used in patients with germline or somatic BRCA1/2-mutated ovarian cancer as maintenance therapy (olaparib)^1249^ or post-chemotherapy therapy (olaparib and rucaparib).^1250^ The FDA has approved olaparib and talazoparib for the treatment of advanced or metastatic HER2- breast cancer patients carrying deleterious germline BRCA1/2 mutations.^1251,1252^ Olaparib is also used for maintenance therapy in patients with germline BRCA1/2-mutated metastatic pancreatic cancer.^1253^ Meanwhile, rucaparib has been applied for second-line treatment of patients with metastatic castration-resistant prostate cancer with germline or somatic BRCA1/2 mutations.^1254^ Significantly, PARP inhibitors have been approved as first-line systemic therapies for patients with ovarian cancer.^1246^ However, acquired resistance to PARP inhibition is still an urgent question, which usually results from three types of mechanisms: drug target-related effects including the upregulation of drug efflux pumps or mutations of PARP or functionally related proteins; restoration of BRCA1/2 function leading to restoration of HR; or loss of DNA end-protection and/or restoration of replication fork stability.^1246^ Therefore, targeted strategies to overcome resistance to PARP inhibitors remain to be explored, and the identification of vulnerabilities of PARP inhibitor-resistant tumors is still challenging. Illustrating the properties of HR-deficient cancers will rationalize the treatment strategies to overcome resistance and improve the survival of patients.^1246^

**Table 9:** The FDA-approved PARP inhibitors in cancer therapies

Drug	Company	Indications	FDA approvals
Olaparib (Lynparza)	AstraZeneca	Ovarian (2014)	Olaparib capsules in patients with BRCA1/2 mutant advanced-stage ovarian cancers who have received ≥3 types of chemotherapies
		Ovarian (2017)	Maintenance therapy for advanced -ovarian cancer patients with PR or CR to platinum-based chemotherapy
		Ovarian (2018)	First-line maintenance therapy for patients with BRCA1/2 mutant advanced-stage ovarian cancers
		Breast (2018)	Patients with BRCA1/2 mutant HER2-negative metastatic breast cancer who have been treated with chemotherapy
		Breast (2022)	Patients with BRCA1/2 mutant HER2-negative high-risk early breast cancer who have been treated with adjuvant chemotherapy
		Pancreatic (2019)	Adult patients with germline BRCA-mutated metastatic pancreatic adenocarcinoma
		Prostate (2020)	Adult patients with HRR gene mutated metastatic castration-resistant prostate cancer
Rucaparib (Rubraca)	Clovis Oncology	Ovarian (2016)	Patients with BRCA1/2-mutant ovarian cancer refractory to ≥ prior lines of treatment
		Ovarian (2018)	Maintenance treatment of patients with recurrent ovarian cancer
		Prostate (2020)	BRCA-mutated metastatic castration-resistant prostate cancer
Niraparib	Tesaro	Ovarian (2019)	Patients with HR deficiency -positive, advanced ovarian cancer
		Ovarian (2020)	First-line maintenance treatment of patients with advanced ovarian cancer
Talazoparib	Pfizer	Breast (2018)	Patients with germline BRCA-mutated, HER2-negative locally advanced or metastatic breast cancer

Source: All the information is derived from the United States Food and Drug Administration.gov (https://www.fda.gov/)

#### ATM inhibitors

ATM is the apical DDR kinase that coordinates DSB repair, and a variety of compounds have been developed for selective inhibition of ATM.^733^ AZD0156 is a potent, selective, and orally active inhibitor of ATM,^1255^ and a phase I clinical trial of AZD0156 (NCT02588105) for safety and preliminary efficacy in advanced solid tumors has been completed, but no results have been posted. AZD1390, belonging to the same potent series as AZD0156, is an exquisitely potent, highly selective, and orally bioavailable ATM inhibitor.^1256^ AZD1390 effectively sensitizes the brain metastasis of breast cancers with DDR mutation to radiation therapy.^1257^ Multiple clinical trials of AZD1390 for cancer treatment are ongoing. M4076, an ATP-competitive ATM inhibitor with an IC_50_ value <1 nM, inhibits tumor cell growth by blocking DSB repair and enhances the sensitivity of tumor cells to radiation therapy both in vitro and in vivo.^1258,1259^ A phase I clinical trial of M4076 in advanced solid tumors is active (NCT04882917). The dual ATM and DNA-PK inhibitor XRD-0394 is a novel, potent, and orally active dual inhibitor.^733^ A phase I clinical trial of XRD-0394 for the treatment of metastatic locally advanced solid tumors and recurrent cancer is recruiting, but no data are yet publicly available (NCT05002140).

Given the important role of ATM in DSB signaling and repair, ATM inhibition combination therapy is currently an attractive strategy for cancer therapy in various clinical trials. ATM inhibitors enhance the anticancer activity of DNA damage agents such as topoisomerase inhibitors^1260^ and PARP inhibitor.^1261^ Collectively, ATM-targeted therapy has a promising potential in cancer therapy.

#### ATR inhibitors

ATR kinase maintains accurate DNA replication by regulating the DNA replication initiation and the process of replication forks, supporting that ATR is an important target for cancer therapy. To date, several ATR inhibitors have been developed.^733,1262^ Ceralasertib (AZD6738) is a selective and potent ATR inhibitor with good solubility, bioavailability, and pharmacokinetic properties.^1263^ Phase II clinical trials of ceralasertib in patients with osteosarcoma (NCT04417062) and advanced solid tumors (NCT04564027) are undergoing. Berzosertib (VX-970) is a highly potent, selective, and intravenous ATR inhibitor with an IC_50_ value of 19 nM.^1264^ A phase II clinical trial of berzosertib in patients with NSCLC (NCT04216316) is ongoing. BAY1895344, another novel potent and selective ATR inhibitor, exhibits strong monotherapy efficacy in cancers, and synergistic activity in combination with DNA damage therapies.^1265^ A phase II clinical trial of BAY1895344 in patients with recurrent solid tumors (NCT05071209) is in progress. Other ATR inhibitors, such as ART0380, ATRN-119, IMP9064, M4344, and RP-3500, are also in clinical trials. Studies also have found synergistic antitumor effects of ATR inhibitors with immunotherapy and other anticancer drugs.^733^ ATR inhibitors in combination with PARP inhibitors are used in the treatment of tumors with BRCA1/2 mutations.^733,1266^ Exploiting combination therapy based on ATR inhibitors may be a promising strategy for cancer therapy.

#### CHK1 inhibitors

CHK1, a downstream effector of ATR, is activated by DDR, and its inhibitors effectively suppress the proliferation of cancer cells with high levels of replication stress.^1267^ Some CHK1 inhibitors have been evaluated or are currently under evaluation in clinical trials, especially in combination with DNA damaging agents such as gemcitabine, cisplatin, and camptothecin.^733,1268^ LY2603618 is the first selective and potent CHK1 inhibitor.^1269^ However, the phase II evaluations of LY2603618 in combination with pemetrexed in patients with advanced NSCLC revealed no significant clinical activity and increased risk of thromboembolic events, which hindered its further development (NCT00988858, NCT01139775).^1270,1271^ MK-8776 (SCH900776) is a selective CHK1 inhibitor that induces cell death when it combines with antimetabolite drugs, such as hydroxyurea, gemcitabine, or pemetrexed in xenograft models.^1269^ However, clinical trials of MK-8776 combined with gemcitabine or cytarabine in patients with solid tumors or hematological malignancies have been completed and the results have shown no positive efficacy but some adverse events such as mucositis, nausea, and prolonged QT interval (NCT01870596, NCT00779584). LY2880070 is an oral, selective competitive CHK1 inhibitor,^1272^ and phase II studies in patients with solid tumors (NCT02632448) and Ewing sarcoma (NCT05275426) are in progress. In addition, LY2606368 (prexasertib) is a CHK1/2 dual inhibitor,^1269,1273^ and a phase I/II study to evaluate the efficacy and safety of LY2606368 in combination with irinotecan and temozolomide in participants with desmoplastic small round cell tumors and rhabdomyosarcoma (NCT04095221) is ongoing. SRA737, an orally bioavailable and selective CHK1 inhibitor, exhibits preclinical activity in MYC-amplified models of neuroblastoma and lymphoma.^1274^ Its phase II clinical trials in patients with advanced solid tumors or NHL (NCT02797964) and in patients with advanced solid tumors (NCT02797977) have been completed. The results have shown that SRA737 is well tolerated. However, further clinical development of SRA737 needs to be performed in combination therapy due to its poor monotherapy activity.^1274^ AZD7762, another ATP-competitive CHK1/2 dual inhibitor, suppresses the CHK1-mediated phosphorylation of CDC25C with an IC_50_ value of 5 nM. However, phase I clinical trials of AZD7762 have been terminated due to its cardiac toxicity and adverse effects.^1269,1275^

In summary, although CHK1 inhibitors are beneficial in preclinical studies, their clinical benefit remains to be confirmed. Studies have found that p53 mutation status may be a key factor in affecting the cell sensitivity to CHK1 inhibitors, suggesting that biomarkers affecting the efficacy of CHK1 inhibitors need to be identified.^1269^ Moreover, the development of novel CHK1 inhibitors with reduced toxicity is meaningful in the near future.

### Targeting tumor inflammation pathways

Inflammation is considered to be one of the key characteristics of tumor initiation, progression, invasion, metastasis, and treatment resistance.^299^ The drugs modulating tumor inflammation pathways mainly include nonspecific agents, such as nonsteroidal anti-inflammatory drugs (NSAIDs), statins, and corticosteroids, and targeted drugs, such as neutralizing antibodies, small molecule inhibitors, and recombinant cytokines^1276^ (Table 10). NSAIDs, including aspirin, celecoxib, and ibuprofen, mainly exhibit anticancer efficiency by inhibiting COX activity and prostaglandin synthesis. NSAIDs have been discovered to reduce cancer mortality, and have shown good therapeutic and preventive effects in cancer patients with CRC, breast cancer, prostate cancer, and head and neck cancer.^1277^ Aspirin, one of the most widely used and typical anti-inflammatory drugs, has been applied as a broad-spectrum cancer-preventive agent. Phase III clinical trials of aspirin for the treatment of patients with gastric cancer (NCT04214990) and CRC (NCT02467582) are underway. Celecoxib, a COX-2 inhibitor, reveals anticancer activity in CRC, breast cancer, prostate cancer, and head and neck cancer.^1278^ A phase IV trial of celecoxib as adjuvant therapy to chemotherapy in subjects with metastasis CRC (NCT03645187) is ongoing. However, long-term treatment with NSAIDs can lead to side effects including mucosal lesions, bleeding, and peptic ulcers. Therefore, balancing the benefits of taking NSAIDs for the prevention and treatment of cancers is essential.^1276^ Statins, consisting of a series of compounds like rosuvastatin, can reduce blood cholesterol concentration by inhibiting the 3-hydroxy-3-methylglutaryl coenzyme A (HMG-CoA) reductase. Statins exert a significant role in antiangiogenic and anti-inflammatory therapy in preclinical studies. However, their clinical benefits remain to be further confirmed.^1279^ Corticosteroids, such as dexamethasone which is usually used as anti-inflammatory drugs for various chronic inflammatory diseases, are found to improve the efficacy of chemotherapy for glioma, breast cancer, lung cancer, and CRC in preclinical studies.^1280^ The strategies that specifically target inflammation pathways mainly include neutralizing antibodies, small molecule inhibitors, and recombinant cytokines.^1276^ As chronic inflammation cytokine IL-6 plays a pivotal role in cancer progression, the IL-6 antibody may exert therapeutic efficacy and benefit to cancer patients. Tocilizumab, a recombinant humanized mAb against the human IL-6 receptor, specifically binds to soluble and membrane-bound IL-6 receptor and inhibits signal transduction. Multiple clinical trials of tocilizumab for the treatment of patients with refractory AML (NCT04000698), NHL (NCT05171647), diffuse large B-cell lymphoma (NCT04408638), and relapsed or refractory follicular lymphoma (NCT04712097) are ongoing. In addition, siltuximab, a mAb against the human IL-6 receptor, was approved by the FDA for treating patients with multicentric Castleman disease on April 22, 2014. Its most common adverse events include pruritus, increased weight, rash, hyperuricemia, and upper respiratory tract infection.^1281^ To date, clinical trials of siltuximab are being conducted to evaluate the efficacy for the prevention of CAR-T cell related cytokine release syndrome in patients with NHL (NCT05665725), for the treatment of patients with metastatic pancreatic cancer (NCT04191421), large granular lymphocytic leukemia (NCT05316116), and multiple myeloma (NCT03315026).

**Table 10:** The clinically anti-inflammatory inhibitors in cancer therapies

Drug	Target	Highest phase	Indications	Identifier	Status
Aspirin	COX-1/2	III	Gastric cancer	NCT04214990	Recruiting
		III	Colon cancer	NCT02467582	Active, not recruiting
Celecoxib	COX-2	IV	Colon cancer stage	NCT03645187	Recruiting
		IV	Hepatocellular carcinoma	NCT02961998	Completed
		IV	Bile duct cancer, pancreatic cancer	NCT01111591	Unknown
		IV	Colorectal cancer	NCT00473980	Completed
Rosuvastatin	HMG-CoA	IV	Prostate cancer metastatic	NCT04776889	Completed
Dexamethasone	Undefined	IV	Metastatic prostate cancer	NCT03432949	Recruiting
		IV	Cancer	NCT02815319	Completed
		IV	Early-stage breast cancer	NCT03348696	Completed
		IV	Pancreatic cancer	NCT04025840	Recruiting
		IV	Lung cancer	NCT02275702	Completed
		IV	Multiple myeloma	NCT01731886	Completed
		IV	Ovarian cancer	NCT00817479	Completed
		IV	Nasal and nasal-type NK/T-cell lymphoma	NCT01501149	Unknown
		IV	Relapsed refractory multiple myeloma	NCT03934684	Active, not recruiting
		IV	Peripheral T cell lymphoma	NCT03071822	Unknown
		IV	Hemophagocytic syndrome T/NK-cell lymphoma	NCT04999878	Recruiting
		Not Applicable	Mammary cancer	NCT05408676	Completed
		IV	Primary CNS lymphoma	NCT01960192	Unknown
		IV	PH+ acute lymphoblastic Leukemia	NCT02690922	Unknown
Tocilizumab	IL-6R-specific antibody	III	Refractory acute myeloid leukemia Refractory acute lymphoblastic leukemia	NCT04000698	Unknown
		III	Non-hodgkin lymphoma	NCT05171647	Recruiting
		III	Diffuse large B-cell lymphoma	NCT04408638	Recruiting
		III	Relapsed or refractory follicular lymphoma	NCT04712097	Recruiting
Siltuximab	anti-IL-6 antibody	II	Lymphoma, non-Hodgkin, multiple myeloma acute lymphoblastic leukemia	NCT04975555	Recruiting
		II	Multiple myeloma AL amyloidosis	NCT03315026	Active, not recruiting
		II	High-risk smoldering multiple myeloma	NCT01484275	Completed
		II	Multiple myeloma	NCT00402181	Completed
		II	Prostate cancer	NCT00433446	Completed
		II	Myeloma	NCT01531998	Completed
		II	Carcinoma, renal cell	NCT00265135	Completed
		II	Ovarian neoplasms, pancreatic neoplasms, colorectal neoplasms, head and neck neoplasms, lung neoplasms	NCT00841191	Completed
		II	Metastatic pancreatic adenocarcinoma, stage IV pancreatic cancer AJCC v8	NCT04191421	Completed
Itacitinib	CXCR4	IV	Lymphoma	NCT05510544	Recruiting
		IV	Non-hodgkin’s lymphoma	NCT01164475	Completed
Ruxolitinib	JAK1/2	IV	Hemophagocytic syndrome, T/NK-cell lymphoma	NCT04999878	Recruiting
Pacritinib	JAK2	II	T-Cell neoplasm lymphoproliferative disorders	NCT04858256	Recruiting
		II	Prostate cancer	NCT04635059	Recruiting
		II	Breast cancer	NCT04520269	Unknown
Bortezomib	NF-κB	IV	Multiple myeloma	NCT02268890	Completed

Source: All the information is derived from the United States Food and Drug Administration.gov (https://www.clinicaltrials.gov)

The significant role of the Janus kinase/signal transducers and activators of transcription (JAK/STAT) signaling pathway in cancer suggests targeting this pathway as a potential anticancer strategy.^1276^ Inhibition of the JAK/STAT signaling pathway has been demonstrated to downregulate cellular proliferation and survival, decrease stem cell properties and inflammatory response, suppress invasion and metastasis, ameliorate immunosuppress in malignant tumors.^1282^ Ruxolitinib is a first-in-class, potent, ATP-competitive, and small molecule JAK1/2 inhibitor (IC_50_ = 3.3 nM for JAK1, 2.8 nM for JAK2) developed by Incyte Corp.^1283^ Following its approval by the FDA for the treatment of diseases, such as multiple sclerosis and vitiligo, the potential of ruxolitinib in cancer therapy has garnered widespread interest. Several clinical trials of ruxolitinib in patients with pancreatic cancer, breast cancer, relapsed or refractory or post myeloproliferative AML, HNSCC, or NSCLC were either terminated or completed with results suggesting insufficient efficacy to justify further investigation. There are still multiple clinical trials undergoing to evaluate ruxolitinib monotherapy or in combination with decitabine for the treatment of accelerated/blast phase myeloproliferative neoplasms (NCT04282187), with trametinib for CRC and pancreatic adenocarcinoma (NCT04303403), with paclitaxel and carboplatin for stage III-IV epithelial ovarian and primary peritoneal cancer (NCT02713386), and with preoperative chemotherapy for triple-negative inflammatory breast cancer (NCT02876302), etc. Pacritinib, a potent inhibitor of JAK2 and FLT-3 with the IC_50_ values of 23 and 22 nM, respectively, was granted accelerated approval by the FDA on February for the treatment of adult patients with intermediate or high-risk primary or secondary myelofibrosis.^1284^ Although some clinical trials of pacritinib, such as those for the treatment of myeloproliferative neoplasmsrefractory CRC or AML, have been affected by increased side effects. Several clinical trials are still undergoing, including pacritinib in combination decitabine or the treatment of accelerated/blast phase myeloproliferative neoplasms (NCT04282187), prostate cancer (NCT04635059), relapsed/refractory T-cell lymphoproliferative neoplasms (NCT04858256). Moreover, other JAK inhibitors such as itacitinib, STAT3 inhibitors like OPB-31121, and TTI-101, and STAT3 antisense oligonucleotide danvatirsen (AZD9150) have been evaluated or are under investigation for their anticancer potential. It is important to note that the clinical studies targeting the JAK/STAT pathway have revealed the complexity of this approach and have underscored the necessity for in-depth investigation to combat cancer more effectively.

Inhibition of NF-κB is also an effective way to slow down tumor development and induce apoptosis in cancer cells. However, NF-κB deficiency can lead to severe immunodeficiency and long-term inhibition of NF-κB causes serious side effects, suggesting that an appropriate dosage regimen and administration time will facilitate NF-κB targeted therapy in the clinic.^764^ Moreover, multiple other inhibitors such as antibodies, cytokines and chemokines inhibitors, and inhibitors of inflammatory transcription factors, are in clinical trials and the results are eagerly anticipated.

### Targeting tumor cell metabolism

The precedent of targeting the metabolism of cancer cells was created by Sydney Farber and colleagues in the 1940s, who successfully used antifolate agents such as aminopterin to induce remission in childhood ALL.^1285^ This discovery led to the development of chemotherapy drugs including methotrexate, 5-FU, gemcitabine, and pemetrexed which are widely used to treat various types of cancers by targeting one-carbon metabolic pathways and their downstream effectors, such as nucleotide metabolism. However, these drugs exhibit many deleterious side effects due to their nonspecific effect and the importance of one-carbon pathways in healthy cells.^1286^

Sixty years after Sidney Farber introduced antifolates for the treatment of childhood ALL, aberrations in cancer metabolism attracted much attention and have been extensively studied, although nearly one century has passed since Otto Warburg discovered aerobic glycolysis in cancer cells in the 1920s. However, therapeutic progress in targeting cancer metabolism remains limited and only a few metabolism-based drugs have been developed, and entered clinical trials for cancer therapy.^1286^

#### Targeting glycolysis

As glucose supplies the major source of energy, carbon intermediates and NADH for biosynthesis, targeted inhibition of glucose uptake and utilization in cancer cells is a promising therapeutic strategy^1287^ (Fig. 6). Multiple inhibitors against glycolytic enzymes and glycolytic product transporter proteins have been studied, such as GLUT1 inhibitors (STF-31, glutor, and BAY-876), HK2 inhibitors (2-deoxyglucose, benitrobenrazide), PKM2 inhibitors (TEPP-46 and mitapivat), LDHA inhibitors (GNE-140, NCI-006, and GSK28387808A), and MCT-1 inhibitors (AZD3965).^1286–1288^ Although these inhibitors have exhibited potent anticancer efficiency in various cancers both in vitro and in vivo preclinically, only a few of them have entered clinical trials, and no clinical success has been achieved thus far due to limited efficacy and toxicity. For example, the MCT-1 inhibitor AZD3965 blocks lactate-mediated tumor progression and has significant anticancer effects alone or combined with metformin. However, a phase I clinical trial in patients with advanced cancers including diffuse large B-cell lymphoma (DLBCL) and Burkitt’s lymphoma, has shown that a number of patients experienced dose-limiting AZD3965 related toxicities such as hematological, cardiac, and ophthalmic toxicities (NCT01791595).

#### Targeting amino acid metabolism

Similarly, amino acids, especially glutamine, participate in various cellular processes in cancer progression, which provide a major source of energy, cell component building blocks, and redox homeostasis, thereby providing a scientific rationale for targeting their metabolism for cancer treatment.^1289^ Anticancer drug candidates against glutamine metabolism and closely linking metabolic networks, such as glutamine transporter SLC1A5, glutaminase (GLS), and aminotransferase, have shown promising effects in cancer treatment (Fig. 6). The amino acid analog l-g-glutamyl-p-nitroanilide, the originally discovered compound V-9302, and specific synthetic mAbs (i.e., KM4008 and KM4012) were developed for the inhibition of SLC1A5. Although they suppressed glutamine-dependent growth of cancer cells to some extent, none of them entered clinical trials, for their specificity, efficiency, and safety profile need to be further evaluated and optimized. In addition to targeting glutamine transporters, targeting GLS, which transforms glutamine into glutamate in the mitochondria, is a notable drug development strategy. Among GLS1 inhibitors, telaglenastat (CB-839), a derivative of the allosteric inhibitor BPTES, has attracted much attention.^1290,1291^ CB-839 has been assessed in more than 10 completed clinical trials alone for the treatment of hematological and solid tumors, or in combination with everolimus for RCC (NCT03163667), talazoparib or palbociclib for solid tumors (NCT03875313, NCT03965845), paclitaxel for TNBC (NCT03057600), and azacitidine for myelodysplastic syndrome (NCT03047993). Disappointingly, CB-839 did not prove efficacious in the above clinical trials or some results were not disclosed. Moreover, some clinical trials of CB-839 alone or in combination with anticancer drugs are ongoing, such as with capecitabine for PIK3CA mutant CRC (NCT02861300), with temozolomide for IDH-mediated diffuse astrocytoma (NCT03528642), with carfilzomib and dexamethasone for recurrent or refractory multiple myeloma (NCT03798678), and with chemoradiation for advanced cervical cancer (NCT05521997). Sirpiglenastat (DRP-104), a glutamine analog that broadly targets glutamine metabolism, is under early-phase clinical trials for examining its efficacy as a single agent or in combination with immune checkpoint inhibitors for advanced cancer (NCT04471415 and NCT06027086).^1292^ The results from the above trials are still pending.

#### Targeting fatty acid metabolism

Cancer cells rely on de novo fatty acid synthesis for proliferation; thus, cancer cells are expected to be vulnerable to the inhibition of fatty acid synthetic enzymes. Inhibitors targeting fatty acid synthase, a key enzyme for de novo fatty acid synthesis, have been developed^1293^ (Fig. 6). Candidates such as TVB316 and TVB2640 have been demonstrated to be effective and less toxic than their predecessors, and more than 10 clinical trials of TVB2640 for the treatment of NSCLC, prostate cancer, and HER2-positive metastatic breast cancer are underway (NCT03808558, NCT03179904, and NCT05743621). The field is anxiously awaiting the results of these studies, decades after fatty acid synthase was identified as a potential cancer therapeutic target. ATP-citrate lyase (ACLY), a key enzyme for fatty acid chain elongation, converts citrate acetyl-CoA into the cytosol. The ACLY inhibitor bempedoic acid was approved by the FDA in 2020 as a lipid-lowering drug.^1294^ Furthermore, a series of allosteric ACLY inhibitors with low (nanomolar) competitive inhibitory activity were discovered, such as the allosteric inhibitor NDI-091143, which binds to homotetramer ACLY, shows potent inhibition and is competitive with citrate and noncompetitive with ATP.^1295^ PF-05221304, an orally administered inhibitor of acetyl-CoA carboxylases (ACC1 and ACC2), is currently undergoing clinical studies (NCT03248882) in nonalcoholic fatty liver disease with fibrosis. Its potential in cancer therapy needs to be further evaluated. ND-646, another allosteric inhibitor of ACC1 and ACC2, reduces tumor growth in NSCLC subcutaneous xenografts, suggesting potential avenues for therapeutic application.^1291^

#### Targeting mitochondria metabolism

The pivotal role of mitochondria as metabolic and biosynthetic organelles makes them attractive anticancer targets (Fig. 6). Although this approach thus far has been limited by toxicity due to difficulties in identifying specific compounds targeting metabolic enzymes, several compounds or candidates targeting the tricarboxylic acid (TCA) cycle and oxidative phosphorylation are currently in clinical trials for the treatment of both solid and hematological tumors.^1296^ The first successful antimetabolite drug came from targeting TCA. IDH catalyzes the oxidative carboxylation of isocitrate to produce α-KG, whereas its mutations result in the gain of function, converting α-KG to the oncometabolite 2-HG.^1297^ In 2017, the FDA approved enasidenib, a first-in-class IDH2 mutation inhibitor developed by Celegene, for the treatment of recurrent or refractory AML with IDH2 mutation. Subsequently, ivosidenib, developed by Agios Pharmaceuticals against IDH1 mutations, was approved by the FDA for the treatment of AML and cholangiocarcinoma with IDH1 mutation.^1298^ Moreover, AG-881 is undergoing clinical trials for the treatment of AML-carrying IDH2 or IDH1/2 mutations (NCT02492737). Similarly, CPI-613, targeting both the α-KG dehydrogenase complex and pyruvate dehydrogenase, is in phase I/II clinical trials for leukemias, lymphomas, and SCLC (NCT03699319 and NCT03793140). In addition to the TCA cycle, the electron transport chain, also known as the respiratory chain which consists of four complexes (CI–IV), is the main target for drug development. Metformin, the most well-known inhibitor of complex I, is approved for type-2 diabetes and has been found to exhibit anticancer effects against various cancers in preclinical studies and clinical trials. In contrast to metformin which is a nonspecific complex I inhibitor, IACS-010759 is a specific inhibitor of complex I that has undergone clinical trials for AML and advanced cancers. However, its toxicity has hindered its further development.^1299^ Rotenone and deguelin also inhibit complex I, while their neurotoxic effects are prominent. Antimycin A is an inhibitor of complex III commonly used in experimental research, while resveratrol has enrolled in clinical trials for different types of cancer. Complex IV can be inhibited by doxorubicin, a DNA intercalating chemotherapeutic drug, and the porphyrin photosensitizer photofrin, which is approved for esophageal cancer and NSCLC. No promising inhibitors have been reported to date for Complex V, except for oligomycin, which is only suitable for experimental use. Employing mitochondrial uncouplers is an alternative approach to impair the function of the electron transport chain. Niclosamide is in phase I/II clinical trials for prostate and colon cancer, while nitazoxanide is in phase II for different forms of advanced cancers.^1296^

#### Targeting one-carbon and nucleotide metabolism

In addition to the metabolism mentioned above, other metabolic processes also perform significant roles in tumors, such as one-carbon metabolism and nucleotide metabolism which have close connections. One-carbon metabolism provides one carbon unit in the form of methyl groups to several metabolic pathways and is responsible for the synthesis of methionine, serine/glycine, purine, and pyrimidine.^1300^ After a landmark study by Farber and colleagues revealed that the folate antagonist aminopterin induced remission in children with ALL, a series of classical inhibitors in this field, including methotrexate, pemetrexed, gemcitabine, and 5-FU, were used as frontline chemotherapy for a diverse range of cancers.^1301^ After that, multiple attempts were made to develop targeted molecules. MTHFD2 inhibitors LY345899 and DS18561882, have shown anticancer activity both in vitro and in vivo.^1302^ PHGDH catalyzes the transformation of the glycolytic intermediate 3-PG into 3-phosphohydroxy pyruvate, and its allosteric inhibitors, such as CBR-5884, NCT-503, α-ketothioamide derivatives, and compound b36, and orthosteric inhibitors which are indole derivatives such as BI-4916, inhibit PHGDH activity and moderately suppress cancer cell proliferation preclinically.^1303^ SHMT catalyzes the conversion of serine and tetrahydrofolate into glycine and 5,10-methylene- tetrahydrofolate, thus providing one carbon unit for nucleotide synthesis. Optimization has generated several experimental dual SHMT1/2 inhibitors, including SHIN1 and SHIN2, which have revealed some extent of anticancer effects preclinically.^1304,1305^ Human dihydroorotate dehydrogenase (hDHODH) is the fourth and rate-limiting enzyme of de novo pyrimidine synthesis, and inhibition of hDHODH is an effective strategy for the treatment of cancers. To date, classical DHODH inhibitors, such as leflunomide and teriflunomide, and several novel hDHODH inhibitors, such as brequinar, ASLAN003, BAY2402234, AG-636, PTC299, and JNJ-74856665 (NCT04609826), have been evaluated in clinical trials to investigate their safety and antitumor efficacy.^1306^

#### Targeting dietary interventions

Dietary interventions alone or in combination with various anticancer strategies have become promising tools for cancer therapy, including preventing tumorigenesis, delaying tumor growth, and improving the effectiveness of existing cancer treatments.^1307^ Dietary interventions potentially improve tumor therapy in several ways, such as eliminating specific nutrients that tumors use as fuel and building blocks, potentiating other forms of therapy including chemotherapy, radiotherapy, and targeted therapy by depriving tumors of nutrients, enhancing the antitumor immune response by modulating the growth factors or altering the systemic immune system.^1308^ Dietary interventions come in various forms, such as the restriction of energy or macronutrients, defined by timing such as intermittent fasting regimens.^1308^ Fasting mimicking diet and intermittent fasting sensitize anticancer medicines. For example, the combination of metformin and intermittent fasting is effective at targeting the metabolic plasticity of cancer. Understanding the interactions between cancer and diet is crucial for establishing diet as a line of treatment. Elucidating altered drug efficacy under a differential metabolic context will be important for future enhancing the dietary interventions in specific cancer therapies due to the heterogeneous nature of cancers and host metabolisms.^1309,1310^

Although extensive efforts have been made to develop targeted therapy against cancer metabolism, few have achieved clinical success, and metabolism-targeted therapy is still challenging for the following reasons. First, metabolism plays a crucial role in all kinds of cells including tumor cells, cells in the TME, such as immune cells, macrophages, cancer-related fibroblasts and other stromal cells, and normal cells. Moreover, extensive interactions of metabolites in these cells exist. These factors make strong antitumor activity and low toxicity by regulating metabolism extremely difficult. Second, both metabolite enzymes and metabolites possess unclassical functions such as acting as kinases or second messengers in addition to acting as enzymes and metabolites. Thus, inhibiting enzyme function may exhibit only moderate anticancer efficiency. Third, cancer cells reprogram their metabolism very quickly after various stimuli by increasing metabolic flexibility, uptake of extracellular metabolites via compensatory transporters and macropinocytosis, and upregulation of nutrient stress-response proteins. Blockage of one pathway by targeting a key enzyme could result in the activation of another metabolic hub. In this regard, targeting cancer metabolism must be based on a thorough understanding of how metabolic pathways affect the whole metabolic status of cancer hubs, which could promote the successful development of anticancer drugs targeting metabolism.

### ICIs-based immunotherapy

Since Tasuku Honjo’s group at Kyoto University discovered PD-1 in 1992,^806^ and Allison’s team at MD Anderson Cancer Center reported that blocking CTLA-4 by its antibody could increase the antitumor activity of T cells and inhibit tumor growth in 1996,^1311^ immunotherapy has been considered as a breakthrough in clinical cancer treatment due to the promising efficacy.^1312^ Immune checkpoint inhibitor (ICI) therapies, including anti-CTLA-4, anti-PD-1, and anti-PD-L1 therapies, have revolutionized the systemic treatments for advanced hematological and solid tumors in the area of antitumor immunotherapy. Compared with chemotherapy and targeted therapies, ICIs induce unprecedented improvements in response rate and better survival rate in partial patients, even after cessation of treatment.^1313,1314^

Ipilimumab, the antibody against CTLA-4, was the first ICI approved by the FDA in 2011, which is a milestone in cancer immunotherapy. Ipilimumab successfully hindered cancer progression in patients with refractory metastatic melanoma.^1315^ Tremelimumab is another human IgG2 CTLA-4 antibody against HCC and was approved by the FDA in 2022.^1316^

#### PD-1/PD-L inhibitors

To date, PD-1/L1 inhibitors are the most widely applied ICIs, which undoubtedly changed the paradigm of cancer therapy. They have shown clinical efficacy against many different solid and hematologic malignancies. The binding of PD-L1 (initially identified as B7-H1) to its receptor PD-1 inhibits T-cell migration, proliferation, and secretion of cytotoxic mediators, thereby limiting tumor cell killing. Inhibitors of PD-1 and PD-L1 reverse T cell suppression by disrupting the PD-1 axis, thereby enhancing the endogenous antitumor immune response.^1312^ Until now, multiple PD-1/L1 inhibitors have been approved for commercialization in the US and China, which include pembrolizumab, nivolumab, dostarlimab, cemiplimab, sintilimab targeting PD-1, and atezolizumab, avelumab, and durvalumab targeting PD-L1 **(**Table 11**)**. These inhibitors are now widely used for the treatment of various cancers, including NSCLC, melanoma, uroepithelial carcinoma, HNSCC, CRC, HCC, and Hodgkin’s lymphoma.^1316^ Nivolumab (Opdivo) and pembrolizumab (Keytruda) are particularly extensively used in clinical therapy. Nivolumab, developed by Bristol Myers Squibb, is the first clinical anti-PD-1 antibody approved in 2015 for the treatment of advanced SCLC and metastatic squamous NSCLC. After that, pembrolizumab, developed by Merck & Co, was approved by the FDA for the first-line treatment of patients with metastatic NSCLC in 2016.^1312^ Different from nivolumab, the prescription of pembrolizumab requires confirmed PD-L1 overexpression on tumors. At the same time, atezolizumab (Tecentriq) by Roche was approved by the FDA in 2016 to treat patients with advanced and metastatic urothelial carcinoma. Another two new PD-L1 antibodies, durvalumab (Imfinzi) and avelumab (Bavencio), were approved in 2017. Furthermore, Innovent Biologics in China developed a PD-1 antibody named sintilimab which achieved good efficiency after neoadjuvant administration.^1317^ Dostarlimab (Jemperli) developed by GSK was approved in 2021 and used in patients with mismatch repair-deficient advanced solid tumors.^1318^

**Table 11:** The FDA-approved immune checkpoint inhibitors

Target	Drugs	Indications	Company
CTLA-4	Ipilimumab	Colorectal cancer, hepatocellular carcinoma, melanoma mesothelioma, non-small cell lung cancer, renal cell carcinoma	Bristol Myers Squibb
PD-1	Cemiplimab	Basal cell carcinoma, cervical squamous cell cancer, non-small cell lung cancer	Regeneron Pharmaceuticals
	Nivolumab	Colorectal cancer, esophageal squamous cell carcinoma, hepatocellular carcinoma, gastric cancer, hodgkin lymphoma, head and neck squamous cell carcinoma, melanoma, mesothelioma, non-small cell lung cancer, renal cell carcinoma, urothelial carcinoma	Bristol Myers Squibb
	Pembrolizumab	Breast cancer, cervical cancer, colorectal cancer, esophageal squamous cell cancer, endometrial carcinoma, esophageal carcinoma, gastric carcinoma, hepatocellular carcinoma, hodgkin lymphoma, non-small-cell lung cancer, melanoma, mesothelioma, Merkel cell carcinoma, non-small cell lung cancer, primary mediastinal large B-cell lymphoma, renal cell carcinoma, small-cell lung cancer, urothelial carcinoma, biliary tract cancer	Merck & Co
	Dostarlimab -gxly	Advanced or recurrent mismatch repair-deficient/microsatellite instability-high endometrial cancer, recurrent or advanced mismatch repair-deficient solid tumors	GlaxoSmithKline
PD-L1	Atezolizumab	Breast cancer, hepatocellular carcinoma, melanoma, non-small cell lung cancer, small-cell lung cancer, urothelial, alveolar soft part sarcoma, urothelial carcinoma	Genentech
	Avelumab	Merkel cell carcinoma, renal cell carcinoma, urothelial carcinoma	EMD Serono
	Durvalumab	Non-small cell lung cancer, small-cell lung cancer, urothelial carcinoma, biliary tract cancer, hepatocellular carcinoma	AstraZeneca UK Limited

Source: All the information is derived from the United States Food and Drug Administration.gov (https://www.fda.gov/)

There are currently three PD-L1 mAbs, atezolizumab, durvalumab, and avelumab, approved by the FDA for the treatment of NSCLC and merkel cell carcinoma. Atezolizumab, a humanized IgG1 mAb, abrogates antibody-dependent cytotoxicity and prevents depletion of PD-L1-expressing T cells.^1316^ Based on its favorable safety and efficacy profile, the FDA accelerated the approval of atezolizumab in May 2016 for the treatment of locally advanced or metastatic urothelial carcinoma treatment after the failure of cisplatin-containing chemotherapy, and subsequently approved for the treatment of advanced metastatic NSCLC during or following platinum-containing chemotherapy in October 2016.^803^ In addition, atezolizumab is the first ICI approved in combination with carboplatin and etoposide to treat advanced SCLC. Durvalumab is a fully human IgG1 mAb that binds PD-L1 with high affinity and specificity.^803^ Durvalumab obtained accelerated approval of the FDA in 2017 for the treatment of patients with advanced or metastatic urothelial carcinoma who have disease progression following platinum-containing chemotherapy, and for the treatment of patients with unresectable stage III NSCLC in 2018.^1319^ Avelumab is a fully human IgG1 mAb with a wild-type IgG1 crystallizable fragment (Fc) region, which enables avelumab to utilize both adaptive and innate immune mechanisms to suppress cancer cells.^1320^ Similarly, avelumab obtained accelerated approval for the treatment of patients with locally advanced or metastatic urothelial carcinoma in 2017, and subsequently approval for first-line treatment of patients with advanced RCC in combination with axitinib in 2019.^1320^ Compared with many oncology regimens, PD-1/PD-L1 blockade is associated with fewer adverse events including fatigue, diarrhea, and decreased appetite which are well tolerated. Moreover, there are still a large number of clinical trials undergoing to evaluate the therapeutic potential of the above inhibitors.

In addition to antibodies, novel strategies targeting PD-1/PD-L1 were developed. For example, AC-1, an antibody-based PROTAC termed AbTAC, simultaneously bound PD-L1 and E3 ligase RNF43 to degrade cell-surface PD-L1 via lysosomal degradation in different cell lines with high PD-L1 expression levels.^1321,1322^ Considering only a small fraction of cancer patients (lower than 50%) respond to PD-1/L1 inhibitors which are far from satisfactory, immune combination therapy which may improve the efficacy and expand the beneficiary population attracted much attention. The combinations of immune checkpoint blockade and costimulatory receptor activation, such as PD-L1 × 4-1BB (MCLA-145) and PD-1 × ICOS (XmAb23104), are under clinical investigation (NCT03922204, NCT03752398). Monovalent trispecific antibody NM21-1480 (αPD-L1, α4-1BB and αHSA) and GNC-038, a tetra-specific IgG-scFv conjugated antibody (αCD19/CD3/4-1BB/PD-L1) are in phase I clinical trials (NCT04442126 and NCT05192486). Checkpoint blockades incorporation with BsAbs achieved tumor-localized and TAA-dependent checkpoint blockage. For example, IBI315, an anti-PD-1 × HER2 developed by Innovent Bridge, is under phase I clinical study for patients with HER2-expressing advanced solid tumors (NCT04162327). Anti-PD-1 × CTLA-4 BsAbs, including AK104, MEDI5752, and MGD019, are expected to synergistically inhibit PD-1 and CTLA-4 double-positive lymphocytes, which are under clinical investigations (NCT06035224, NCT04522323, and NCT05293496).

#### Other ICIs

In addition to PD-1/PD-L1 and CTLA-4, novel immune checkpoints including lymphocyte activation gene-3 (LAG-3), T cell immunoglobulin and mucin-domain-containing 3 (TIM-3), and T cell immunoglobulin and ITIM domain (TIGIT) that mediate inhibitory signals through different mechanisms have been identified, and their inhibitors have been emerging for cancer immunotherapy.^1312^ The mAb drugs targeting these immune checkpoints transmissed inhibitory signals following ligand engagement, and their synergistic antitumor effect with PD-1/PD-L1 inhibitors were evaluated in preclinically and in multiple clinical trials (NCT03219268, NCT03708328, and NCT03440437).

Induced on CD4+ and CD8 + T cells under antigen stimulation, LAG-3 has become one of the most promising new targets of immune checkpoint blockage after PD-1 with great application prospects. Relatlimab is the most advanced mAb targeting LAG-3, which is under phase II/III clinical trial in unresectable or untreated metastatic melanoma in combination with nivolumab. The study resulted in a median PFS of 10.12 months in the combination group compared with 4.63 months in the monotherapy group, supporting its approval by the FDA for the treatment of metastatic melanoma combined with nivolumab. Relatlimab represents the third type of ICI to enter the market.^1316^ TM-3 is a T-cell surface inhibitor that is mainly expressed on CD4 + T helper cell 1 (Th1) and CD8 + CTL cells, and some innate immune cells including dendritic cells, NK cells, and macrophages. LY3321367, an anti-Tim-3 antibody, demonstrated good tolerability as monotherapy or in combination with an anti-PD-L1 antibody in phase I studies, and further clinical studies are needed to verify its efficacy and safety in larger cohorts of patients.^1323,1324^ TIGIT, a type I transmembrane protein which belongs to the immunoglobulin superfamily (IgSF), is expressed on T cells, regulatory T cells, memory T cells, and NK cells. Tiragolumab is the mAb targeting TIGIT which is currently under phase III clinical trial in extensive-stage SCLC in combination with atezolizumab (NCT04256421). In addition, mAbs targeting fibrinogen-like protein 1 (FGL1), a ligand for Lag-3 for NSCLC, nuclear receptor subfamily 2 group F member 6 (NR2F6), an intracellular IC molecule, and V-set immunoregulatory receptor (VISTA), an immunomodulatory protein expressed in lymphoid organs and bone marrow cells, are now being evaluated in phase I clinical studies for the treatment of solid tumors.^1312^

At present, approximately5000 registered clinical studies listed on the US trial registry site ClinicalTrials.gov are ongoing to evaluate the effectiveness of ICIs involving PD-1, PD-L1, or CTLA-4, both individually and in combinations against various hematological and solid tumors. Although ICIs have achieved great success in clinical treatment, some challenges still remain to be solved in this field. First, only parts of patients significantly benefit from ICI treatment. Thus, accurate prediction biomarkers by integrating multiple approaches to determine which patients are likely to benefit from ICIs is urgently needed. The combination and development of multiple functional approaches, including large-scale genomic sequencing, single-cell transcriptomic techniques, multi-omics, and computational immunogenomics, which integrate intratumour heterogeneity, tumor mutational burden, neoantigen expression, and immunogenicity, could improve the prediction of response to ICIs.^1325^ Second, although ICIs initially exhibited strong efficiency against tumor growth, patients still have relapse and/or develop acquired resistance. Combinational therapeutic strategies based on a deep understanding of the tumor and TME, and coordination of systemic and local intratumor immune responses enable to improve and maximize the potential benefit to more tumor patients.^1326^ Third, the development of novel cancer immunotherapy targets based on the mechanistic study can lead to the discovery of effective approaches, which in turn improve the efficacy of tumor immunotherapy. In summary, ICIs opened a new era of immunotherapy and changed the landscape of cancer treatment. They are promising treatment options although the response rate is far from satisfactory. Combination therapy and mechanism studies may improve efficacy, expand the beneficiary population, and further support immunotherapy as a mainstream cancer treatment alongside chemotherapy, radiotherapy, and surgery.

### Differentiation therapy

The concept of differentiation therapy originates from the fact that hormones or cytokines can promote differentiation in vitro and thus irreversibly alter the phenotype of cancer cells. Certain signaling molecules and drugs, such as retinoic acid, cAMP, sodium butyrate, and cytokines, can induce terminal ex vivo differentiation in AML, embryonic carcinomas, or neuroblastoma.^1327^

Differentiation therapy is a meaningful tumor-targeting strategy, and many inhibitors have been developed to induce differentiation. The combination of small molecule inhibitors all-trans retinoic acid and arsenic trioxide for the treatment of acute promyelocytic leukemia is a watershed for differentiation therapy. In addition, retinoic acid is used to treat solid tumors. For example, retinoic acid induces the differentiation of tumor-initiating cells in HCC, suppresses the expression of stem cell markers, and induces the expression of liver-specific genes, ultimately increasing the sensitivity of cisplatin therapy. The small molecule drug arsenic trioxide, approved by the FDA for leukemia treatment, has shown effectiveness in various hematological malignancies and solid tumors. The natural product oroxylin A, a bioactive flavonoid in Scutellaria baicalensis with strong anticancer effects and safety, can induce tumor cell differentiation. Oncostatin M, a glycoprotein belonging to the IL-6 family of cytokines, is involved in cell growth and development and can induce differentiation and inhibit the proliferation of HCC cells.^821^

Since most solid tumor oncogenic signaling pathways are far more genetically complex than the genetic basis of leukemia, the efficacy of solid tumor differentiation inducers has not yet reached that of hematologic malignancies. Conventional cancer therapy aims to kill rapidly proliferating tumor cells, which can damage normal cells and lead to serious side effects. In contrast, differentiation therapies have low cytotoxicity and are effective in combination with classical tumor-killing cytotoxic compounds. Differentiation therapy reduces malignancy and inhibits the aggressiveness of tumors. Tumor differentiation therapy has many benefits, including reversing the malignant phenotype of tumors, restoring normal cellular functions, enhancing the immunogenicity of tumor cells, and enhancing the therapeutic sensitivity of tumor cells to conventional tumor therapy and ICIs.^821^ Therefore, the induction of cancer cell differentiation is a valuable tumor treatment strategy, which warrants further study.

### Epigenetic reprogramming inhibitors

Epigenetic dysregulation in cancer has led to the exploration of epigenetic machinery as a promising target for drug development. Consequently, the field of developing epigenetic drugs, which target enzymes involved in regulating genome function through epigenetic mechanisms, has gained significant attention.^1328^ Currently, the focus of epigenetic drug development revolves around enzymes responsible for introducing (writers), recognizing (readers), and removing (erasers) epigenetic marks on DNA or core histones.^1329^ Inhibitors have been designed to target these enzymes, including DNMTs, and HMTs EZH2 and DOT1L as writers, HDM LSD1 and HDACs as erasers, and BET proteins as readers (Table 12).

**Table 12:** The FDA-approved and clinically developed epigenetic reprogramming inhibitor in cancer therapies

Type	Drug	Highest Phase	Indications	Company/Identifier	Status
DNMTi	Azacitidine (Vidaza)	Approved	Acute myeloid leukemia, chronic myelomonocytic leukemia, myelodysplastic syndromes	Celgene	/
	Decitabine (Dacogen)	Approved	Acute myeloid leukemia, chronic myelomonocytic leukemia, myelodysplastic syndromes	MGI Pharma & SuperGen	/
	Guadecitabine	III	Acute myeloid leukemia	NCT02920008	Completed
		III	Myelodysplastic syndromes, leukemia myelomonocytic chronic	NCT02907359	Completed
		III	Leukemia myeloid acute	NCT02348489	Completed
	NTX-301	I	Acute myeloid leukemia, myelodysplastic syndromes, chronic myelomonocytic leukemia	NCT04167917	Recruiting
	MG98	I	Unspecified adult solid tumor	NCT00003890	Completed
HDACi	Belinostat (Beleodaq)	Approved	Peripheral T-cell lymphoma	Spectrum Pharma	/
	Panobinostat (Farydak)	Approved	Multiple myeloma	Novartis	/
	Romidepsin (Istodax)	Approved	Cutaneous T cell lymphoma	Gloucester Pharma	/
	Vorinostat (Zolinza)	Approved	Cutaneous T cell lymphoma	Merk Sharp	
	Abexinostat	III	Renal cell carcinoma	NCT03592472	Recruiting
	ACY-241	I	Multiple myeloma	NCT02400242	Active, not recruiting
		I	Malignant melanoma	NCT02935790	Completed
		I	Advanced solid tumors	NCT02551185	Completed
		I	Non-small cell lung cancer	NCT02635061	Active, not recruiting
	AR-42	III	Neurofibromatosis type 2	NCT05130866	Recruiting
	CUDC-907	II	Relapsed and/or refractory diffuse large B-cell lymphoma including myc alterations	NCT02674750	Completed
		II	Thyroid neoplasms, poorly differentiated and undifferentiated thyroid cancer, differentiated thyroid cancer	NCT03002623	Terminated
		II	Prostate cancer	NCT02913131	Terminated
	CXD101	II	Colorectal neoplasms malignant	NCT03993626	Unknown
		II	Diffuse large B-cell lymphoma	NCT03873025	Withdrawn
		II	Hepatocellular carcinoma	NCT05873244	Recruiting
	Entinostat	III	Advanced breast cancer	NCT03538171	Unknown
		III	Breast adenocarcinoma, HER2/Neu negative locally advanced breast carcinoma, metastatic breast carcinoma, recurrent breast carcinoma	NCT02115282	Active, not recruiting
	Givinostat (ITF2357)	II	Multiple myeloma	NCT00792506	Terminated
		II	Chronic myeloproliferative neoplasms	NCT01761968	Active, not recruiting
		II	Hodgkin’s lymphoma	NCT00496431	Terminated
		II	Hodgkin’s lymphoma	NCT00792467	Completed
	Mocetinostat (MGCD0103)	II	Urothelial carcinoma	NCT02236195	Completed
		II	Lymphocytic leukemia chronic	NCT00431873	Completed
		II	Hodgkin’s lymphoma	NCT00358982	Terminated
		II	Lymphoma	NCT00359086	Completed
		II	Myelogenous leukemia acute, myelodysplastic syndromes	NCT00374296	Terminated
		II	Lymphoma relapsed and refractory, diffuse large B-cell lymphoma and follicular lymphoma	NCT02282358	Terminated
		II	Myelodysplastic syndrome, acute myelogenous leukemia	NCT00324220	Completed
	Resminostat (4SC-201)	II	Hepatocellular carcinoma	NCT00943449	Completed
		II	Advanced colorectal carcinoma	NCT01277406	Completed
		II	Hepatocellular carcinoma	NCT02400788	Completed
		II	Hodgkin’s lymphoma	NCT01037478	Completed
		II	Lymphoma	NCT02953301	Active, not recruiting
	Ricolinostat (ACY-1215)	II	Multiple myeloma	NCT01997840	Active, not recruiting
		II	Lymphoma, Lymphoid malignancies	NCT02091063	Completed
		II	Multiple myeloma	NCT01323751	Completed
EZH2i	Tazemetostat	Approved	Epithelioid sarcoma	Epizyme	/
	GSK126	I	Cancer, Neoplasms	NCT02082977	Terminated
	CPI-1205	II	Metastatic castration-resistant prostate cancer	NCT03480646	Unknown
	CPI-1205	I	Advanced solid tumors	NCT03525795	Terminated
	CPI-1205	I	B-cell lymphoma	NCT02395601	Terminated
DOT1Li	Pinometostat (EPZ-5676)-	II	Recurrent/refractory acute myeloid leukemia	NCT03701295	Completed
		II	Acute myeloid leukemia	NCT03724084	Terminated
LSD1i	ORY-1001	I	Small cell lung cancer	NCT02913443	Completed
		I	Acute myeloid leukemia	NCT05546580	Recruiting
	INCB059872	II	Solid tumors and hematologic malignancy	NCT02712905	Terminated
		II	Solid tumors	NCT02959437	Terminated
		II	Myeloproliferative neoplasms, myelodysplastic syndrome	NCT04061421	Recruiting
	IMG-7289	II	Acute myeloid leukemia	NCT02842827	Completed
		II	Myelofibrosis	NCT03136185	Completed
		II	Thrombocythemia	NCT04081220	Recruiting
		I	Acute myeloid leukemia	NCT05597306	Recruiting
		II	Extensive stage lung small cell carcinoma	NCT05191797	Recruiting
	GSK2879552	I	Carcinoma small cell	NCT02034123	Terminated
		I	Leukemia myelocytic acute	NCT02177812	Terminated
	CC-90011	I	Lymphoma	NCT02875223	Active, not recruiting
		I	Small cell lung carcinoma	NCT03850067	Active, not recruiting
		II	Neoplasms	NCT04350463	Active, not recruiting
		I	Prostatic neoplasms	NCT04628988	Completed
		I	Leukemia	NCT04748848	Terminated
	SP-2577	II	Ewing sarcoma	NCT05266196	Enrolling by invitation
		II	Recurrent chronic myelomonocytic leukemia	NCT04734990	Active, not recruiting
BETi	TEN‐010	I	Acute myeloid leukemia, myelodysplastic syndromes	NCT02308761	Completed
		I	Multiple myeloma	NCT03068351	Completed
		I	Solid tumors	NCT01987362	Completed
	GSK525762	II	Neoplasms	NCT01943851	Completed
		I	NUT midline carcinoma	NCT01587703	Completed
	OTX105	I	Acute myeloid leukemia	NCT01713582	Completed
		I	NUT midline carcinoma	NCT02259114	Completed
	CPI-0610	I	Multiple myeloma	NCT02157636	Completed
		I	Lymphoma	NCT01949883	Completed

Source: All the information is derived from ClinicalTrials.gov (https://www.clinicaltrials.gov) and the United States Food and Drug Administration.gov (https://www.fda.gov/)

#### DNMT inhibitors

DNMT inhibitors, also known as hypomethylating agents, have become effective epigenetic therapies for cancer due to the crucial role of DNMTs in DNA methylation. There are two main classes of DNMT inhibitors: nucleoside analog inhibitors that are incorporated into newly synthesized DNA and recognized by DNMTs, and nonnucleoside inhibitors that interfere with DNMT binding.^1330^ Nucleoside inhibitors, such as 5-azacitidine (Vidaza), 5-aza-2′-deoxycytidine (decitabine, Dacogen), and SGI-110 (guadecitabine), belong to the first class. These cytidine analogs irreversibly sequester DNMT proteins into DNA, resulting in global DNA hypomethylation and reactivation of silenced genes in cancers. Currently, 5-azacitidine and decitabine have been approved for treating AML, chronic myelomonocytic leukemia, and MDS.^1331^ However, these drugs have notable side effects, including cellular and clinical toxicity, as well as chemical instability.^1331^ Next-generation nucleoside analog DNMT inhibitors, such as guadecitabine, exhibit improved pharmacokinetic profiles with longer plasma half-life and lower peak plasma concentrations, leading to reduced toxicity. Guadecitabine has shown promising outcomes in clinical studies for AML treatment.^1332^ Another novel hypomethylating agent called NTX-301 has demonstrated superiority over conventional agents such as 5-azacitidine and decitabine in preclinical studies, which supports ongoing clinical development efforts.^1333^ Clinical trials with NTX-301 are currently underway (NCT04167917, NCT03366116, and NCT04851834).

Nonnucleoside DNMT inhibitors directly target the catalytic site of specific DNMT enzymes, unlike nucleoside analogs that are incorporated into DNA. Researchers have discovered reversible and selective nonnucleoside DNMT inhibitors, including RG108, SGI-1027, GSK3685032, DC-05, and CM-272.^1334^ Additionally, MG98 is an antisense oligodeoxynucleotide that targets the 3′UTR of DNMT1 mRNA to achieve DNA demethylation.^1335^ However, further preclinical and clinical studies are necessary to determine if their outcomes are more favorable than those of nucleoside analogs.

#### HDAC inhibitors

Many HDAC inhibitors target Zn^2+^ in the active site of HDACs to inhibit their enzymatic activity, leading to changes in chromatin structure and gene expression. Five HDAC inhibitors have been globally approved, including vorinostat (SAHA), romodepsin, belinostat, panobinostat (approved by the FDA), and chidamide (also known as tucidinostat, approved by the NMPA) for specific hematologic malignancies.^1336^ In 2006, the FDA approved the first HDAC inhibitor, vorinostat, for the clinical treatment of cutaneous T-cell lymphoma.^1337^ Additionally, the FDA has successively approved romidepsin, belinostat, and panobinostat as HDAC inhibitors for the treatment of peripheral T-cell lymphoma, T-cell lymphoma, and multiple myeloma, respectively.^1336^ Since these drugs are all pan-inhibitors of HDAC and may exhibit potential toxic side effects, the first selective HDAC subtype inhibitor, tucidinostat, has been approved for the treatment of recurrent or refractory PTCL and breast cancer.^1338^

Currently, available HDAC inhibitors are nonselective or pan-HDAC inhibitors, which have drawbacks such as poor efficacy on solid tumors, limited therapeutic efficacy, drug resistance, and toxicity.^1339^ Thus, developing HDAC inhibitors with better activity and higher selectivity is an important area of research. Three schemes for designing HDAC inhibitors include drug design based on zinc-binding groups, selective inhibitors targeting different subtypes of HDAC, and dual mechanism or multitarget HDAC inhibitors.^1340^ Many HDAC inhibitors have been synthesized based on these schemes, with more than ten entering clinical trials.^1341^ For example, abexinostat, a potent oral pan-HDAC inhibitor designed based on ZBG, is currently undergoing phase II clinical trials for recurrent/refractory DLBCL and follicular lymphoma as a single-drug treatment (NCT03936153 and NCT03934567). Additionally, the combination of abexinostat and pazopanib for locally advanced or metastatic RCC has entered the phase III trial (NCT03592472). Another instance is entinostat, a synthetic benzamide derivative HDAC inhibitor that selectively inhibits class I and IV HDAC enzymes.^1342^ Entinostat has been evaluated in several phase II trials in patients with breast cancer and has improved patient median OS (NCT00676663). Furthermore, the combination of entinostat and exemestane for advanced breast cancer has entered phase III clinical trials (NCT03538171 and NCT02115282).

#### HMT inhibitors

In addition to broad epigenetic reprogrammers such as DNMT inhibitors and HDAC inhibitors, targeted therapy has been developed for specific mutations in epigenome-modifying enzymes.^1343^ For instance, tazemetostat (EPZ-6438, E7438) is used for patients with EZH2 mutations. Tazemetostat is the only approved EZH2 inhibitor granted FDA approval in 2020 for treating epithelioid sarcoma and follicular lymphoma based on clinical trials (NCT01897571 and NCT02601950).^1344^ Ongoing clinical trials are assessing its efficacy against various hematologic malignancies and solid tumors (NCT05567679, NCT04179864, NCT05023655, NCT04705818, and NCT04846478). Studies have shown that loss of Bap1 in mice can increase EZH2 expression and H3K27me3 levels. Moreover, mesothelioma cells with BAP1 mutations have shown sensitivity to EZH2 inhibition, leading to clinical trials investigating the use of tazemetostat in treating malignant mesothelioma with BAP1 inactivation (NCT02860286).^1345^

GSK126 is a potent and highly selective EZH2 inhibitor that targets both wild-type EZH2 and Y641 mutant EZH2.^1346^ However, a phase I clinical trial assessing its safety, pharmacokinetics, pharmacodynamics, and clinical activity in patients with relapsed or refractory DLBCL, other NHLs, multiple myeloma, and solid tumors was terminated. The results showed insufficient antitumor activity and a relatively short half-life, which limited effective exposure and did not support further clinical study (NCT02082977). On the other hand, CPI-1205 is an orally bioavailable EZH2 inhibitor with a unique indole-based structure different from pyridine-based compounds such as GSK126 and tazemetostat.^1347^ Preclinical studies have demonstrated that CPI-1205 significantly inhibits tumor growth in DLBCL xenograft models, with good oral bioavailability and an acceptable safety profile in rats and dogs. Currently, CPI-1205 is undergoing evaluation in a phase I clinical trial in patients with B-cell lymphoma (NCT02395601), advanced solid tumors (NCT03525795), and metastatic castration-resistant prostate cancer (CRPC) (NCT03480646).

DOT1L, the sole identified H3K79 methyltransferase, has been targeted for cancer treatment, especially in acute leukemias with MLL gene rearrangements.^1348^ Pinometostat, the first clinical inhibitor of DOT1L, exhibits improved potency, longer plasma half-life, enhanced selectivity, and efficacy in reducing leukemic cell proliferation. Phase I clinical trials have been conducted for MLL-rearranged leukemia using pinometostat. Although well tolerated, pinometostat requires continuous IV infusion due to rapid clearance and shows modest clinical effectiveness (NCT02141828 and NCT01684150).^1349,1350^

#### HDM inhibitors

Preclinical studies have shown LSD1 inhibitor-dependent differentiation and growth inhibition, which has led to the initiation of several clinical trials to assess its efficacy. These trials include iadademstat (ORY-1001), INCB059872, IMG-7289, GSK2879552, seclidemstat, and pulrodemstat (CC-90011).^1351^

Oryzon Genomics developed ORY-1001 in 2012, which uses TCP as the lead compound and is currently being studied in clinical trials for AML and SCLC (NCT05546580 and NCT02913443).^1352^ INCB059872, an irreversible LSD1 inhibitor reported by Lee et al. in 2016, has undergone five clinical trials to test its safety and efficacy in treating solid tumors and hematologic malignancies (NCT02712905, NCT02959437, NCT03132324, NCT03514407, and NCT04061421). While one phase I study was terminated due to sickle cell disease risks, other trials have shown potential in treating various cancers. IMG-7289 (Biomedestat), developed by Imago BioSciences in 2018, is currently undergoing clinical trials for treating AML and MDS (NCT02842827), bone marrow fibrosis (NCT03136185) and essential thrombocythemia (NCT04081220). GSK2879552, another irreversible LSD1 inhibitor, has shown antitumor activity in AML and SCLC but had to be discontinued in three clinical trials due to unfavorable risk-benefit profiles (NCT02034123, NCT02177812, and NCT02929498).

Two reversible LSD1 inhibitors, CC-90011 and SP-2577 (Seclidemstat), have undergone preclinical studies and clinical trials, showing promising results for treating different types of tumors. Celgene developed CC-90011, the first reversible LSD1 inhibitor, for clinical trials targeting relapsed or refractory solid tumors and NHL (NCT02875223), as well as advanced solid and hematological tumors (NCT03850067, NCT04350463, NCT04628988, and NCT04748848). Salarius Pharmaceuticals developed SP-2577, another LSD1 reversible inhibitor, which showed a manageable safety profile in a phase I clinical trial for advanced solid tumors (NCT03895684). Additionally, clinical trials have been initiated to investigate the combination of SP-2577 with topotecan, cyclophosphamide (NCT03600649), and azacytidine (NCT04734990) for cancer treatment.

#### BET inhibitors

In contrast to targeting the enzymatic domains responsible for modifying epigenetic marks, an alternative strategy focuses on inhibiting the proteins that recognize these modifications by disrupting protein–protein interactions. A prime example of this innovative approach is BET inhibitors, which disrupt the interaction between bromodomains and acetylated lysine residues. This disruption interferes with the recruitment of transcriptional machinery to specific gene loci.^1353^

Initial small molecule BET inhibitors such as JQ1 have revealed the oncogenic role of BET proteins and their impact on oncogene expression, leading to observed antitumorigenic effects in preclinical models. However, their clinical application has been hindered by poor pharmacokinetics, short half-life, and low oral bioavailability.^1354^ TEN-010 (RO6870810), a JQ1 derivative with improved pharmacological properties, has undergone clinical trials for AML, MDS, and solid tumors (NCT02308761 and NCT01987362). Other BET inhibitors have also advanced to clinical trials. For instance, GSK525762, evaluated in multiple studies for hematologic malignancies and solid tumors, has shown encouraging results, especially in NUT midline carcinoma, AML, and TNBC patients (NCT01943851 and NCT01587703). Further research is needed to establish its efficacy and safety. OTX015, another BET inhibitor, has demonstrated favorable antitumor activity, particularly in combination therapies, in clinical evaluations for various hematologic malignancies and solid tumors (NCT01713582 and NCT02259114). CPI-0610, a selective BET inhibitor, has entered the clinical trial for hematologic malignancies such as myelofibrosis and lymphoma to evaluate its safety, tolerability, and potential efficacy as monotherapy and combination therapy (NCT01949883). Early studies have shown promising activity, but some patients experienced adverse events such as thrombocytopenia and moderate diarrhea (NCT02157636).

In addition, efforts have been made to design PROTACs and molecular glues that can degrade BET proteins by utilizing the intracellular ubiquitin proteasome system.^1343^ A clinical trial is currently underway for the FHD-609 degrader in the treatment of synovial sarcoma (NCT04965753), and the CFT8634 degrader has recently entered phase I/II clinical trials for synovial sarcoma and SMARCB1-null solid tumors (NCT05355753).

#### IDH inhibitors

Mutations in isocitrate dehydrogenases IDH1 and IDH2, commonly found in lower-grade gliomas as well as in AML and other malignancies, result in neomorphic enzyme activity, leading to increased production of 2-HG from α-KG.^1355^ 2-HG serves as a competitive inhibitor of various α-KG-dependent dioxygenases, such as the Jumonji-C domain family of histone demethylases and the TET family of DNA demethylases, disrupting the global methylation landscape and promoting cancer development by impairing cellular differentiation.^1356^ Consequently, mutant IDH has emerged as an appealing therapeutic target, leading to the development of several IDH inhibitors aimed at counteracting the effects of 2-HG.

Enasidenib, the first FDA-approved IDH2-mutant inhibitor, received approval in 2017 after positive results from a single-arm trial on relapsed or refractory AML patients with IDH2 mutations (NCT01915498). Similarly, based on favorable outcomes observed in a clinical trial (NCT02074839), the FDA approved ivosidenib, an IDH1-mutant inhibitor, for relapsed or refractory AML patients with IDH1 mutations.^1357^ More recently, the combination of ivosidenib and azacitidine received FDA approval for newly diagnosed, IDH1-mutated AML following a phase III trial (NCT03173248).^1358^ In 2021, ivosidenib was also approved by the FDA for advanced cholangiocarcinoma with IDH1 mutation after a phase III clinical trial (NCT02989857).^1359^ These examples highlight the success of biomarker-driven approaches in treating cancers with epigenetic alterations, emphasizing the importance of considering chromatin changes when evaluating drug targets that indirectly modulate chromatin.

Another strategy to target IDH1 genetic alterations in gliomas is the development of vaccines. The most common IDH1 mutation found in gliomas is the Arg132 mutation, resulting in the production of a tumor-specific neoantigen called IDH1(R132H). Several IDH1(R132H)-specific peptide vaccines are currently undergoing testing as monotherapy or in combination with other therapies (NCT02454634, NCT03893903, NCT02193347, and NCT02771301).^1360^

Despite the theoretical significance and rationale of epigenetic therapy, there are still several issues that need to be discussed and resolved. The first issue relates to selectivity, specifically how to selectively target widely expressed epigenetic regulators. Epigenetic events are generally present in both normal and cancer cells. However, certain cancers rely on specific epigenetic changes and are sensitive to their regulation. It is crucial to identify the most critical epigenetic changes in different types of cancers. The second issue is the differential susceptibility of hematologic malignancies and solid tumors to epigenetic intervention. While significant progress has been made in epigenetic therapy for hematologic malignancies, solid tumors have been less responsive. The complexity of the oncogenome and inherent cellular differences between hematologic malignancies and solid tumors may contribute to this variation in efficacy. Understanding these biological principles is essential for expanding the application of epigenetic therapy to solid tumors. Moreover, the heterogeneity and plasticity of human cancer highlight the importance of personalized and precise epigenetic therapies. Precision medicine approaches, such as using high-throughput epigenomics sequencing technology, can help create genome and epigenome maps of individual patient’s tumor cells. These maps can then be used for drug sensitivity testing and screening, enabling optimized treatments tailored to each patient.

### Targeting tumor microbiomes

Targeted interference with gut and tissue-resident microbiota or microorganism-derived products-based therapies are effective ways to target tumors^854^ (Table 13). Several strategies are applied to target gut and tumor microorganisms, including fecal microbial transplantation (FMT), single strains or designer consortia-based targeted microbial strategies, diet-based and prebiotic, probiotic, and postbiotic-based interventions, as well as targeted antibiotic approaches.^854^ FMT, a promising way to modulate the gut microbiome, acts by transplanting the entire gut microbial complement from a donor such as a healthy individual into a recipient such as a patient with cancer. The early phase I study of FMT revealed that it is beneficial to treat steroid-refractory GI tract graft-versus-host disease, which is a complication of hematopoietic stem cell transplantation to treat leukemia.^854,1361^ While FMT methods transplant the entire donor microbiota, the specific transplantation of single microbial species or designer microbial consortia to improve treatment is needed in certain circumstances. For example, CBM588, a formulation that includes a strain of *Clostridium butyricum*, improves the PFS of patients with RCC in combination with immune checkpoint blockade therapy.^854,1362^ Moreover, numerous dietary strategies, such as long-term caloric restriction, short-term starvation, and ketogenic diets, have shown the potential to enhance the efficacy of immunotherapy.^854^ The dietary interventions have been widely applied in cancer therapy for target gut and tumor microorganism. However, lack of rigorous standard procedures and poor association between diet and clinical effects have hindered their clinical application.^854^

**Table 13:** The typical and clinically developed microbiota inhibitor

Drug	Highest Phase	Indications	Identifier	Status
Bismuth colloidal pectin granules quadruple therapy	IV	Gastric cancer, helicobacter pylori infection	NCT04660123	Completed
	IV	Gastric cancer, helicobacter Pylori infection	NCT04209933	Completed
Itraconazole	IV	Hematologic neoplasms	NCT02895529	Terminated

Source: All the information is derived from ClinicalTrials.gov (https://www.clinicaltrials.gov)

In addition, the probiotic and postbiotic-based interventions and targeted antibiotic approaches also play significant roles in targeting gut and tumor microorganisms.^854^ A study has found that oral probiotic candidate DTA81 is effective in preventing the development of early CRC.^853,1363^ In addition, *H. pylori* infection is an important oncogenic factor in gastric cancer and gastric mucosa-associated lymphoid tissue lymphoma.^1364^ Combination therapy of antisecretory proton pump inhibitors with the antibiotics amoxicillin, levofloxacin, clarithromycin, and metronidazole is the standard protocol for *H. pylori* eradication.^853,1364^ In addition, phytomedicines and probiotics are also used to treat *H. pylori* infections.^1365^ Bismuth collisional tuberculosis has been used in clinical trials to treat the eradication rate of *H. pylori* infection in cancer patients, as well as to evaluate the improvement of symptoms and the incidence of adverse reactions.^853^ Itraconazole is a broad-spectrum triazole antifungal agent with favorable pharmacodynamic and pharmacokinetic profiles and is used for the prevention or treatment of systemic fungal infections.^1366,1367^ Itraconazole inhibits cell proliferation, invasion, and migration of oral OSCC cells by suppressing the Hedgehog pathway-induced cell cycle arrest and apoptosis.^1368^ Moreover, itraconazole can inhibit the proliferation and growth of CRC cells by promoting autophagy and apoptosis, and it is an effective treatment for CRC.^1369^ In summary, the microbiota is an important driver of cancer, and targeting the microbiota in the gut is meaningful in precision cancer care. However, the field requires further elucidation of the specific mechanisms by which microorganisms impact cancer processes. Shortly, targeting microbiota in the gut may emerge as a promising tool for cancer care.

### Therapeutic strategies for targeting cellular senescence

Induction of tumor cell senescence has been demonstrated as one of the underlying mechanisms by which cancer therapies such as radiation, chemotherapy, and targeted therapy exert their antitumor activity. Paradoxically, lingering senescent cells (SnCs) in tumor tissues fuel tumor progression, relapse, and metastasis partly through the expression of the SASP.^865^ Based on the above observations, targeted therapeutics inducing tumor cell senescence followed by senolytics to selectively clear newly induced SnCs, which is called “one-two punch” cancer therapy, represent an emerging and promising new strategy in cancer treatment. Moreover, the application of senomorphic drugs which reduce the production and secretion of SASP factors has attracted attention in cancer therapy.^1370,1371^

#### Therapy-induced senescence (TIS)

The currently available targeted therapeutics for inducing senescence include blocking the cell cycle, triggering DNA damage, manipulating epigenetic modulators, and regulating tyrosine kinases. As cell cycle arrest is a hallmark of senescent cells, drugs that inhibit CDK or enhance levels of CDK inhibitor proteins are currently being used in senescence-inducing cancer therapy.^1372^ In particular, CDK4/6 inhibitors such as palbociclib, abemaciclib and ribociclib, which are approved by the FDA for the treatment of advanced breast cancer, are able to induce senescence in various cancer cells.^864^ PF-06873600, a triple CDK2/4/6 inhibitor, is a potent senescence inducer in various cancer models, and is ongoing in breast cancer in combination with endocrine therapy.^1373^ AURKs and PLKs, which are serine/threonine kinases essential for cell mitosis, are potential targets for senescence-inducing therapy.^1370^ Multiple PLK1 inhibitors, such as BI-6727, and AURK inhibitors, such as alisertib, are currently undergoing clinical investigation (NCT02273388 and NCT06095505).

Triggering DNA damage is another strategy to induce senescence. For example, PARP inhibitors, including veliparib and olaparib, induce a reversible senescent phenotype caused by BCL-X_L_ mediated resistance to apoptosis in ovarian cancer, breast cancer, and prostate cancer. Inhibition of DNA replication through small molecule inhibition of the kinase CDC7 using XL413 or TAK-931 leads to senescence induction in liver cancers.^1374^ Inhibition of the telomerase complex has been identified as inducing replicative senescence in anticancer therapy. Imetelstat, GX301, and BIBR1532, potent telomerase inhibitors, effectively induce senescence and suppress cancer cell proliferation in preclinical or clinical trials.^1370,1375^

Another approach to induce senescence is by modulating the epigenome of cancer cells. Decitabine, a DNMT inhibitor, and vorinostat, an HDAC inhibitor, upregulate the expression of multiple tumor suppressor genes, such as *CDKN2A* and *TP53*, thus inducing cellular senescence via these pathways in various cancer cells.^865,1376^

Moreover, numerous other drugs and antibodies can induce senescence in cancer cells. Tamoxifen, an estrogen receptor antagonist, and bicalutamide, an androgen receptor antagonist, can induce senescence in breast cancer or prostate cancer.^1377^ Trastuzumab and pertuzumab, which are antibodies targeting HER2, cause senescence in breast cancer. BRAF and MEK inhibitors, such as vemurafenib and trametinib, show great senescence-inducing effects in melanoma.^1378^

#### Senolytics

Although TIS contributes to antitumor effects and treatment outcomes, increasing evidence has demonstrated that the accumulation of SnCs can stimulate the relapse and metastasis of cancers. Thus, selective clearance of SnCs with senolytics will prevent tumor relapse and metastasis, overcome drug resistance, and minimize toxic side effects.

To date, drugs or compounds targeting the apoptosis modulator BCL-2/BCL-X_L_, PI3K-AKT-mTOR, BET, tyrosine kinases, and GLS have exhibited promising effects on the clearance of senescent cells.^865,1370^ Apoptosis resistance is a feature shared by both cancer and senescent cells; thus, blocking antiapoptotic proteins could selectively eliminate senescent cells. ABT-737 was one of the first senolytics selectively targeting BCL-2, BCL-X_L_, and BCL-W, thus removing SnCs by reactivating the apoptotic pathway.^475^ After that, Navitoclax (ABT-263), a Bcl-2/Bcl-xL inhibitor, and venetoclax (ABT-199), a BCL-2 inhibitor, were developed and used as adjuvant therapies with radiation to selectively eliminate TIS cells, and increase the survival of tumors, including glioblastoma, melanoma, and lung cancer cells.^1379,1380^ In addition, the PI3K/AKT inhibitors dasatinib and quercetin selectively kill senescent cells and reduce the secretion of proinflammatory cytokines.^862^ An mTOR inhibitor significantly reduced the tumor burden and increased survival in xenograft cancer models after treatment with a DNA-replication kinase CDC7 inhibitor, which induced senescent liver and lung cancer cells.^1374^ Another drug screen identified the BET family protein degrader as a senolytic drug and validated that ARV825, a PROTAC of BET, possesses strong senolytic activity.^1381^

Immune-targeted therapy may be an effective way to clear senescent cells.^862^ For example, chimeric antigen receptor T cells targeting the cell surface protein, urokinase-type plasminogen activator receptor were found to be effective in clearing senescent cells after mice with lung adenocarcinomas were exposed to MEK and CDK4/6 inhibitors to induce senescence.^1382^

#### Senomorphics

Preventing the development of SASP or improving SASP-related functions can reduce inflammation and cancer risk. Multiple signaling pathways are involved in regulating SASP function, including p38/MAPK, JAK/STAT, mTOR, NF-κB, and C/EBP-β.^1383^ The inhibitors that modulate the excretion of SASP are called senomorphics which potentially preserves the SASP-dependent protumor effects of senescent cells and exert a synergistic antitumor effect.^865^ For example, IL-6 mAb siltuximab, multiple signal inhibitor metformin, and the JAK inhibitor ruxolitinib reduced SASP, thereby reducing the protumor and damage induced by SASP.^865,1383^ In addition, the protein arginine methyltransferase (PRMT1) induces SASP at the promoter of proinflammatory genes. The PRMT1 inhibitor TC-E increases the apoptosis sensitivity of cancer cells by regulating the NF-κB pathway.^862^ Rapamycin, an inhibitor of mTOR, has been found to reduce SASP by inhibiting mTOR and to limit the growth-promoting effect of senescent bystander fibroblasts on prostate cancers.^1384,1385^

Although targets and several senolytics have been discovered and tested in preclinical or clinical settings, the development of senolytics is still a challenge for the following reasons. First, TIS is heterogeneous and context (e.g., tissue of origin, time after treatment) dependent. Therefore, a deeper understanding of tumor contexts is critical for the usage and development of novel drugs that induce senescence. Second, the different types of prosenescence drugs induce cellular senescence via different mechanisms, suggesting that different TIS cells may require different senolytics. Moreover, the characteristics of SnCs, and the physiological and pathogenic effects of SnCs have not been adequately identified. Thus, the discovery of novel senolytic targets, senolytics, and the optimization of prosenescence therapy and senolytic combinations need further investigation. Third, senescence-inducing drugs cause senescence not only in tumor tissues but also in the TME and normal tissues. Induction of senescence may induce immune escape which decreases the anticancer effect or causes unwanted side effects associated with lingering senescent cells. The selectivity of senescence-inducing should be improved. Last, as surveillance of SnCs is executed by cytokines and chemokines which are released in a time-dependent manner, the timing of senolytic intervention is crucial for the efficacy of the one-two punch strategy and to avoid side effects on normal tissues. Taken together, despite the various challenges, senotherapies is a promising strategy and is likely to be applied in the clinic in the future as our understanding of senescence improves.

## Conclusion and perspectives

“It is far more important to know what sort of person the disease has than what sort of disease the person has”, which is put forward by Hippocrates and precisely interprets the trend of cancer management. Current cancer management aims to combine molecular data with traditional clinical information, such as symptoms, personal history, and histology, to tailor medical care with the most benefit and minimize risk. Meaningfully, multifarious biomarkers are conducive to monitoring and predicting the therapeutic response of precision and personalized medicine in clinical practice,^1386^ which highlights the pivotal position of tumor biomarkers in cancer therapy.

Tumor biomarkers, biologically indicating pathogenic processes or pharmaceutical responses to therapeutic interventions,^145^ consist of six different types of biomarkers that are biomarkers of early detection, diagnosis, prognosis, prediction, therapeutic target, and surrogate end point.^145^ The biomarkers of early detection are essential in screening patients with cancers at an early stage. The diagnostic biomarkers mainly contribute to the identification of the presence and characteristics of cancers. The prognostic biomarkers indicate the disease outcome of patients to achieve individualized management, and the predictive biomarkers are utilized to demonstrate the treatment response of patients, thereby identifying the best therapy. Thus, prognostic biomarkers can identify patients who are at high-risk of cancer. The predictive biomarkers suggest the patients that can benefit from a specific therapy.^114^ In addition, biomarkers of therapeutic targets can identify the molecular targets of novel therapies. Moreover, surrogate endpoint-related biomarkers are used as substitutes for clinical endpoints or to assess clinical benefits, such as posttherapy PSA changes to evaluate drugs in the clinic.^145^ Herein, we mainly summarize the development history, detection methods, and classification of tumor biomarkers, thereby illustrating the crucial roles of tumor biomarkers in cancer screening, diagnosis, treatment, prognosis, and targeted therapy.

Although tumor biomarkers are increasingly critical in cancer precision medicine, the biomarkers surviving from discovery to clinical trials are small in number.^1386^ Some challenges are still urgently being solved in the future. First, the characteristics and concentration of tumor biomarkers are influenced by different biologic factors, such as posttherapy, host heterogeneity, age and the presence of other diseases among different individuals, false positive biomarkers generated by other physiologic or pathologic processes, and exogenous interfering substances, i.e., foods, drugs, and natural alternative therapies. Second, it is necessary and urgent to explore novel tools or technologies that could discover novel and accurate biomarkers for the detection of preneoplastic neoplasia, micrometastatic spread, and states of early or aggressive cancer recurrence. Third, limitations in analytical sensitivity and specificity still exist. Clinical detection and measurement assays of biomarkers require sufficient sensitivity and improved specificity. Standard procedures, clear guidelines, and quality control schemes are essential to ensure accuracy and reproducibility for biomarker development.^145^ Fourth, the high risk of false positives arises when identifying a biomarker from thousands of molecules. Thus, the cost of massive screening multiple times and the increased risk of false positives of overdiagnosis need to be balanced. Consistent adherence to publishing guidelines of tumor biomarkers can improve transparency and better judge the quality of putative biomarker identification.^1386^ Artificial intelligence offers an intriguing opportunity in large-scale screens of available data and to develop novel tumor biomarkers.^1387^ Finally, a rational combination of various biomarkers could improve the efficiency and accuracy of the application of biomarkers. Extensive research along with the use of new technologies needs to be performed.

In conclusion, we see great enthusiasm in tumor biomarker discovery and application from the large number of studies in the late century. Subsequently, the continuous research and development of innovative tumor biomarkers and the continuous development of novel detection technologies will make it possible for sensitive and specific tumor biomarkers to be gradually applied in clinical practice, and make early screening, diagnosis, treatment, and prognosis assessment of tumors a reality.
